# An Integrated Genomic Approach for Rapid Delineation of Candidate Genes Regulating Agro-Morphological Traits in Chickpea

**DOI:** 10.1093/dnares/dsu031

**Published:** 2014-10-21

**Authors:** Maneesha S. Saxena, Deepak Bajaj, Shouvik Das, Alice Kujur, Vinod Kumar, Mohar Singh, Kailash C. Bansal, Akhilesh K. Tyagi, Swarup K. Parida

**Affiliations:** 1 National Institute of Plant Genome Research (NIPGR), Aruna Asaf Ali Marg, New Delhi 110067, India; 2 National Research Centre on Plant Biotechnology (NRCPB), New Delhi 110012, India; 3 National Bureau of Plant Genetic Resources (NBPGR), New Delhi 110012, India

**Keywords:** chickpea, SSR, SNP, QTLs, transcription factor, wild

## Abstract

The identification and fine mapping of robust quantitative trait loci (QTLs)/genes governing important agro-morphological traits in chickpea still lacks systematic efforts at a genome-wide scale involving wild *Cicer* accessions. In this context, an 834 simple sequence repeat and single-nucleotide polymorphism marker-based high-density genetic linkage map between cultivated and wild parental accessions (*Cicer arietinum desi* cv. ICC 4958 and *Cicer reticulatum* wild cv. ICC 17160) was constructed. This inter-specific genetic map comprising eight linkage groups spanned a map length of 949.4 cM with an average inter-marker distance of 1.14 cM. Eleven novel major genomic regions harbouring 15 robust QTLs (15.6–39.8% *R*^2^ at 4.2–15.7 logarithm of odds) associated with four agro-morphological traits (100-seed weight, pod and branch number/plant and plant hairiness) were identified and mapped on chickpea chromosomes. Most of these QTLs showed positive additive gene effects with effective allelic contribution from ICC 4958, particularly for increasing seed weight (SW) and pod and branch number. One robust SW-influencing major QTL region (*qSW4.2*) has been narrowed down by combining QTL mapping with high-resolution QTL region-specific association analysis, differential expression profiling and gene haplotype-based association/LD mapping. This enabled to delineate a strong SW-regulating *ABI3VP1* transcription factor (TF) gene at trait-specific QTL interval and consequently identified favourable natural allelic variants and superior high seed weight-specific haplotypes in the upstream regulatory region of this gene showing increased transcript expression during seed development. The genes (TFs) harbouring diverse trait-regulating QTLs, once validated and fine-mapped by our developed rapid integrated genomic approach and through gene/QTL map-based cloning, can be utilized as potential candidates for marker-assisted genetic enhancement of chickpea.

## 1. Introduction

Chickpea [*Cicer arietinum* (L.)] belongs to the member of West Asian Neolithic crop assemblage which is associated with the origin and evolution of ancient agriculture in the central part of the Fertile Crescent ∼10,000–12,000 years ago.^1^ Chickpea is believed to have originated from areas of present-day South Eastern Turkey and Syria, where three wild annual *Cicer* species, namely *Cicer reticulatum*, *Cicer echinospermum* and *Cicer bijugum*, are found. The genus *Cicer* comprises 44 species, including 35 wild perennials, 8 wild annuals and 1 cultivated annual.^2,3^ All these annual *Cicer* species have an identical ploidy level and chromosome number (2*n* = 2*x* = 16)^4,5^ with an expected genome size of ∼740 Mb.^6^ The 44 annual and perennial *Cicer* species are grouped into three gene pools based on their crossability relationships with cultivated chickpea.^7^ The primary gene pool comprises cultivated *C. arietinum* species and its progenitor, *C. reticulatum*, which is freely crossable with cultivated chickpea (*desi* and *kabuli*) for a normal gene exchange.^8^ The secondary gene pool consists of *C. echinospermum*, a species which is crossable with cultivated chickpea, but hybrids result in reduced fertility. The tertiary gene pool consists of remaining 6 annual and 35 perennial species that are not readily crossable with cultivated species, which requires some specialized techniques for their gene transfer in the cultivated background. As far as crossability of the wild species with cultivated gene pool is concerned, attempts were made to exploit the wild annual *Cicer* species for genetic base broadening, which suggests that *C. reticulatum* and *C. echinospermum* species can be inter-crossed with the cultivated *C. arietinum* species.^9–11^ Moreover, these annual wild species signify a valuable and potential source of diverse gene pool for improving yield and abiotic/biotic stress tolerance in cultivated chickpea.^12–17^ Many studies have introduced the genetically diverse wild relatives with beneficial traits (primarily belonging to primary and secondary gene pools) through inter-specific hybridization for enhancing the seed and pod yield potential and stress tolerance in chickpea cultivars.^13,14,16,18^ To accelerate such process of genetic enhancement in chickpea, the use of efficient and modern genomics-assisted breeding approaches like trait-associated genes/quantitative trait loci (QTLs) identification from wild accessions and their marker-aided introgression into cultivated gene pool are much required at present.

The discovery and impact of sequence-based robust simple sequence repeat (SSR) and single-nucleotide polymorphism (SNP) markers in view of their desirable genetic attributes (co-dominant inheritance, reproducibility, bi-/multi-allelic nature and abundant genomic distribution) have been well studied in chickpea^8,19–37^ for various large-scale genotyping applications including construction of high-resolution genome map and identification/mapping of genes/QTLs regulating important agronomic traits. Earlier, low-scale genotyping information (allelic variants) specifically of a limited number of SSR markers scanned from inter-specific bi-parental mapping population (*C. arietinum* × *C. reticulatum*) involving cultivated and wild accessions have been utilized to map QTLs controlling important traits of interest, including beta-carotene, seed weight, fusarium wilt and ascochyta blight resistance in chickpea.^8,38–41^ The latest draft genome sequences of *desi* and *kabuli* chickpea cultivars^42,43^ enabled to select a large number of genome-wide SSR and SNP markers from pseudomolecules of eight chromosomes based on their physical positions (bp) for uniform as well as high saturation genome and gene/QTL mapping in chickpea. No such systematic efforts have yet been made at a genome-wide scale involving wild *Cicer* accessions for identification and fine mapping of robust QTLs/genes controlling important agronomic traits in chickpea. Henceforth, novel allelic variants mined from wild accessions by high-throughput genotyping of genome-wide informative SSR and SNP markers in an advanced generation bi-parental mapping population could be useful to identify robust and major QTLs regulating important agro-morphological traits in chickpea through genetic/QTL mapping. The robust trait-influencing major QTLs identified and mapped on chickpea genome/chromosomes can be refined by combining QTL mapping with QTL region-specific high-resolution association analysis, differential expression profiling and gene haplotype-based association/linkage disequilibrium (LD) mapping to delineate trait regulatory genes, alleles and haplotypes at target QTL intervals in chickpea. The major advantages of such integrated approaches lies in narrowing down the long trait-specific QTL intervals to specific candidate genes regulating complex quantitative traits including low-phosphorous stress tolerance in soybean.^44^ Overall, the combinatorial genomic approach would facilitate the identification of more robust trait-regulatory functionally relevant molecular tags (QTLs, genes, alleles and superior haplotypes) from the diverse gene pools of wild accessions with potential known source of important agronomic traits (including abiotic and biotic stress tolerance) for genetic improvement of cultivated chickpea through marker-assisted introgression breeding and to develop high-yielding durable stress tolerant (climate resilient) chickpea cultivars.

Keeping all above in view, this study was undertaken to construct a high-density inter-specific genetic linkage map by high-throughput genotyping of 839 genomic and genic SSR and SNP markers (physically mapped across eight chickpea chromosomes) showing polymorphism between parental accessions (*C. arietinum desi* cv. ICC 4958 and *C. reticulatum* wild cv. ICC 17160) of a 229 F_7_ RIL mapping population (ICC 4958 × ICC 17160) using gel-based assay, fluorescent dye-labelled automated fragment analyzer and 34-plex matrix-assisted laser desorption ionization-time of flight (MALDI-TOF) mass array. The large-scale genotyping information of these markers in RILs was correlated/integrated with their replicated multi-location field phenotyping data to identify the novel genomic regions harbouring the major and robust QTLs associated with four agro-morphological traits through genetic/QTL mapping in chickpea. One of the strong seed weight-associated genomic region underlying robust QTL was targeted to delineate functionally relevant candidate genes, favourable natural allelic variants and superior haplotypes regulating seed weight by combining QTL mapping with QTL region-specific high-resolution association analysis, differential gene expression profiling and gene haplotype-based association/LD mapping in chickpea.

## 2. Materials and methods

### 2.1. Plant materials used for genomic DNA extraction

A 229 F_7_ RIL mapping population (advanced by single seed descent method) derived from the inter-specific crosses between two parental chickpea accessions (ICC 4958 × ICC 17160) was developed for identification and mapping of QTLs. ICC 4958 (developed by Jawaharlal Nehru Krishi Viswa Vidyalaya, Jabalpur, Madhya Pradesh, India) is a high branch number, pod number and 100-seed weight, and lightly pubescent plant hairiness type annual cultivated *desi* (*C. arietinum*) accession. In contrast, ICC 17160 (originated from Turkey) is a low branch number, pod number and 100-seed weight, and densely pubescent plant hairiness type annual wild (*C. reticulatum*) accession. A seed weight-specific association panel comprising 244 chickpea accessions (including 167 *desi* and 77 *kabuli* accessions) constituted previously by Kujur *et al.*^37^ was selected (Supplementary Table S1) for the QTL region-specific trait association analysis. Additionally, 81 accessions belonging to five annual wild chickpea species, namely *C. reticulatum*,^16^ *C. echinospermum,*^8^ *C. judaicum,*^22^ *C. bijugum*^19^ and *C. pinnatifidum*^16^ (Supplementary Table S2), were used for gene haplotype-specific association/LD mapping and haplotype sharing-based gene evolution study. The genomic DNA was isolated from the young leaf samples of natural association panel (244 cultivated and 81 wild chickpea accessions) as well as 229 RILs and two parental accessions using QIAGEN DNeasy 96 Plant Kit (QIAGEN, USA) following the manufacturer's instructions.

### 2.2. High-throughput genotyping of SSR and SNP markers

A set of 496 genic and genomic SSR markers (Supplementary Table S3) and 384 transcription factor (TF) gene-derived SNP markers (Supplementary Table S4) were selected based on their genome-wide physical distribution on eight chickpea chromosomes (Supplementary Fig. 1). Four hundred and seventy SSR markers showing polymorphism between parental accessions (ICC 4958 and ICC 17160) were polymerase chain reaction (PCR) amplified and genotyped in 229 RIL mapping individuals using the 3.5% metaphor agarose gel and fluorescent dye-labelled automated fragment analyzer following Kujur *et al*.^36,37^ A set of 369 TF gene-derived SNP markers showing polymorphism between parents were further genotyped in RILs using Sequenom MALDI-TOF MassARRAY system (http://www.sequenom.com). The designing of MassARRAY multiplex iPLEX assay, amplification of synthesized pre-amplification primers with multiplex PCR assay, shrimp alkaline phosphate incubation, primer extension, resin clean up and mass spectrometry were performed following the manufacturer's instructions of Sequenom iPLEX Gold amplification kit (http://www.sequenom.com) and methods of Pandit *et al.*^45^ The SNP genotyping information generated in iPLEX spectrochip bio-arrays was analyzed in MassARRAY Typer 3.4 and allele-specific differences in mass between extension products were documented.

### 2.3. Evaluation of agronomic traits

The 229 RIL mapping individuals and two parental accessions (ICC 4958 and ICC 17160) were grown (planted in a single row with 35 × 10 cm spacing) in the field for two consecutive years (2011 and 2012) as per randomized block design. At least two replications were performed during crop season (minimum 12 ± 2°C and maximum 22 ± 3°C) at two diverse geographical locations of India (New Delhi: latitude 28.6°N and longitude 77.2°E and Palampur: 32.1°N and 76.5°E). The seed weight (SW) (by taking average weight of 100 matured seeds at 10% moisture content), number of pods (NP) (by counting average number of fully formed pods per plant at maturity), number of branches (NB) (average number of branches emerging per plant at time of harvest) and plant hairiness (PH) (determining presence/absence of hairs/trichomes at vegetative and maturity stages) were estimated by selecting 10–12 representative plants from each RILs. The mean, standard deviation, coefficient of variation (CV), frequency distribution and ANOVA of phenotyping data in mapping population were evaluated following Kujur *et al.*^36,37^ The broad-sense heritability (*H*^2^ = σ^2^g/σ^2^p) was measured by comparing the genotypic (σ^2^g) and phenotypic (σ^2^p) variance of each trait using the methods of Holland *et al.*^46^ The Pearson's correlation coefficient and its significance (*t*-test) among all pairwise trait combinations were estimated using SPSS v17.0 (http://www.spss.com/statistics). Moreover, 244 *desi* and *kabuli* chickpea accessions belonging to a SW-specific association panel and 81 wild chickpea accessions were grown in the field and phenotyped for SW following aforesaid methods of mapping population.

### 2.4. Construction of an inter-specific genetic linkage map and QTL mapping

The SSR and SNP marker genotyping data showing goodness of fit to the expected Mendelian 1:1 segregation ratio were utilized to construct an inter-specific genetic linkage map using MAPMAKER/EXP3.0^47^ and JoinMap 4.1 (http://www.kyazma.nl/index.php/mc.JoinMap^48^) at higher LOD (logarithm of odds) threshold (>4.0) with Kosambi mapping function. The markers were integrated into eight linkage groups (LGs) of an inter-specific genetic map based on their centimorgan (cM) genetic distance using the methods of Kujur *et al.*^36,37^ The LGs were designated (LG1–LG8) according to their corresponding marker physical positions (bp) on chromosomes as determined in our study.

The QTL mapping was performed by integrating the genotyping data of parental polymorphic 470 SSR and 369 SNP markers mapped on eight LGs with replicated multi-location/years field phenotyping data (PH, SW, NP and NB) of 229 RIL mapping individuals. The single marker analysis, interval mapping and composite interval mapping functions of window QTL cartographer WinQTLCart v2.5^49^ and MapQTL 6^50^ with LOD >4.0 at 1,000 permutations were considered significant (5% significance level) to identify and map the novel genomic regions harbouring the QTLs associated with traits under study in chickpea. The percentage of phenotypic variation explained (PVE) by QTLs (*R*^2^%) and their additive effect (evaluated by parental origin of favourable alleles) on traits at significant LOD (*P* ≤ 0.05) was determined. However, permutation test was unable to detect any such QTL when LOD threshold values for all traits under study were below 4.0 at 5% significance level. The QTLs with consistent PVE (>10%) and validated across multiple geographical locations as well as years/seasons were defined as ‘major’ and ‘robust’ QTLs.

### 2.5. Targeted multiplexed amplicon resequencing and association analysis

One selected strong SW-regulating major genomic region harbouring robust QTL was sequenced in parental accessions (ICC 4958 and ICC 17160) and five of each homozygous low and high seed weight RIL mapping individuals using the multiplexed amplicons sequencing protocol of TruSeq Custom Amplicon v1.5 in Illumina MiSeq next-generation sequencer (Illumina, USA). This genomic region underlying QTL was targeted to design and synthesize custom oligo probes using Design Studio software. All the probes were pooled into a custom amplicons tube to produce ∼600 amplicons (average 400 bp amplicon size) per reaction, and template library was generated using TruSeq Custom Amplicon Assay kit v1.5. Twelve sample-specific indices were added to each library by PCR using common primers from the TruSeq Amplicon Index kit. The uniquely tagged pooled amplicon libraries were normalized, generated clusters and sequenced by Illumina MiSeq platform (http://www.illumina.com/systems/miseq/system.ilmn). The sequenced amplicons and sequence variants were visualized using Illumina Amplicon Viewer. The high-quality and filtered amplicon sequence reads (>90% bases covered at 0.5× mean coverage) for each accession were mapped to reference *kabuli* genome.^43^ The SNPs were detected among accessions as per Agarwal *et al.*^51^ and Jain *et al.*^42^ The sequenced region was structurally and functionally annotated, and genes annotated in the target genomic region underlying SW QTL were predicted using *kabuli* genome annotation database.^43^

A set of 34 SSR and 192 SNP (showing polymorphism between ICC 4958 and ICC 17160, and differentiating low and high seed weight RILs) markers (mapped with an average of 1.5 kb uniform sequence intervals) were selected from different coding and non-coding sequence components of genes and intergenic regions annotated at SW QTL interval of interest. These markers were genotyped in 244 *desi* and *kabuli* chickpea accessions belonging to a SW-specific association panel^37^ using the gel-based assay, automated fragment analyzer and MALDI-TOF SNP genotyping assay. The genotyping information of markers at SW QTL interval, replicated multi-location field phenotyping (SW) and population structure (*K* = 2) data and kinship matrix of 244 accessions (association panel) were analyzed in general linear model (GLM) and mixed linear model (MLM) of TASSEL v2.1 adopting the detailed procedure of Kujur *et al.*^37^ Based on combined results of GLM and MLM, the markers showing strong association (*R*^2^, magnitude of significant correlation of markers with traits) with SW at significant cut-off value of *P* ≤ 10^−2^ were selected for further analysis.

### 2.6. Expression profiling

To infer the differential expression patterns of genes underlying a robust SW-associated major QTL interval, suitable gene-specific primer pairs were designed for expression profiling. The RNA was isolated from five different vegetative and reproductive tissues (shoot, root, leaf, flower bud and pod) and two seed developmental stages [early cell division phase during 10–20 days after podding (DAP) and late maturation phase during 21–30 days after DAP] of low and high seed weight mapping parents (ICC 4958 and ICC 17160) and two contrasting accessions (ICCX-810800, *desi* small, 100-seed weight 11 g and ICC 20268, *kabuli* large, 47 g). The isolated RNA was amplified using the gene-based primers along with internal control elongation factor 1-alpha (*EF1α*) by semi-quantitative and real-time quantitative RT–PCR assays following Kujur *et al.*^36^ The expression level of genes estimated in diverse tissues and seed developmental stages of four chickpea accessions were compared with each other and along with control (vegetative leaf tissue of respective accessions) to identify the differentially regulated genes.

### 2.7. Molecular haplotyping

For high-resolution haplotype-based association/LD mapping, the fragments amplified covering the entire coding sequences (CDS) and 1-kb upstream regulatory region (URR) of one strong trait-associated TF gene (validated by QTL mapping, trait association mapping and differential expression profiling) in 244 cultivated *desi* and *kabuli* accessions (association panel) and 81 wild chickpea accessions (Supplementary Table S1 and S2) were cloned and sequenced following the study by Kujur *et al.*^36,37^ The generated high-quality gene sequences were aligned among accessions using CLUSTALW multiple sequence alignment tool in MEGA v4.0^52^ and mined the SNP loci. The genotyping information of SNPs mined in the TF gene among accessions was used to constitute haplotypes, and the SNP-based haplotype diversity and LD patterns within the gene were estimated. For association analysis, the SNP-based haplotype genotyping information in the TF gene was further correlated with SW trait of 244 cultivated and 81 wild chickpea accessions using the aforesaid methods. To access the potential of diverse haplotypes constituted in the TF gene for regulating seed weight, the differential expression profiling in two seed developmental stages of contrasting accessions representing low and high seed weight haplotype groups was performed using the gene haplotype-specific primers.

## 3. Results and discussion

### 3.1. Construction of a high-density inter-specific chickpea genetic linkage map

**Figure 1.**
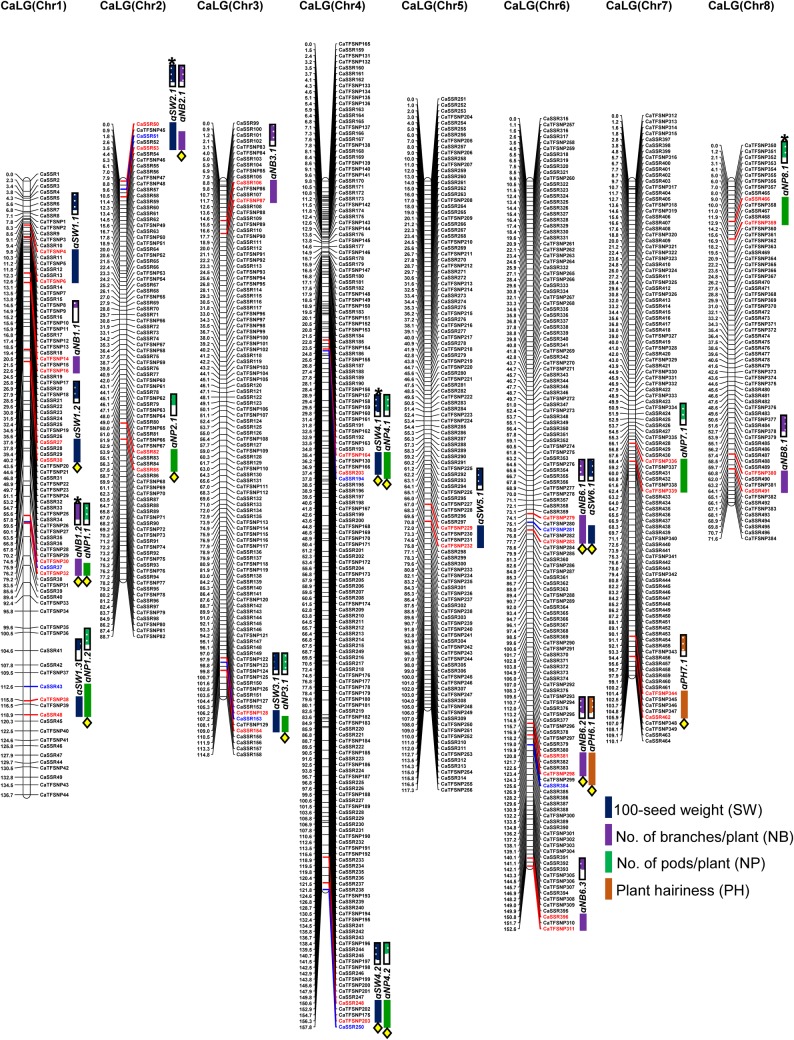
Nineteen genomic regions harbouring 27 significant QTLs (15.6–39.8% *R*^2^) associated with SW, NP, NB and PH were identified and mapped on eight LGs (LOD > 4.0 at *P* < 0.05) using a 229 RIL mapping population (ICC 4958 × ICC 17160) of chickpea. The genetic distance (cM) and identity of the marker loci integrated on the chromosomes are indicated on the left and right sides of the LGs, respectively. The 15 major and robust QTLs are marked with yellow boxes. The marker pairs flanking the QTLs are coloured with red and blue. Blue, violet, green and brown lines indicate the QTLs regulating SW, NB, NP and PH mapped on eight LGs. The direction of QTLs (additive effects) are designated with empty (ICC 17160-specific alleles) vs. filled (ICC 4958-specific alleles) boxes. *qSW2.1, qSW4.1, qNP8.1 and *qNB1.2* correspond to known QTLs from previous studies by Cobos *et al.*,^53,54^ Hossain *et al.*,^55^ Varshney *et al.*^56^ and Gowda *et al.*^57^ This figure appears in colour in the online version of *DNA Research*.

A set of 839 including 470 genic and genomic SSR markers (Supplementary Fig. S2) and 369 TF gene-derived SNP markers (Supplementary Fig. S3) showing polymorphism between parental accessions were genotyped in 229 individuals of a RIL mapping population (ICC 4958 × ICC 17160) to construct an inter-specific genetic linkage map in chickpea. The linkage analysis using the markers showing significant Mendelian segregation ratio (1:1) mapped 834 markers (including 468 SSR and 366 SNP markers) on eight LGs (LG1–LG8) of chickpea (Fig. 1) according to their physical positions (bp) on respective chromosomes (Supplementary Fig. S1). The constructed genetic map comprising eight LGs spanned a total map length of 949.42 cM with an average inter-marker distance of 1.138 cM (Supplementary Table S5). The map length covered by each LG varied from 71.57 cM (62 markers) in LG8 to 157.80 cM (162 markers) in LG4. The most saturated LG was LG4 (average inter-marker distance of 0.974 cM), whereas LG1 was least saturated (1.647 cM) (Supplementary Table S5). Assuming ∼740 Mb genome size of chickpea, the 834 marker-based genetic linkage map had mean marker density of one marker per 887.3 kb. Interestingly, 76 major hot-spot regions were identified on eight LGs of genetic map in which more than two markers were mapped and clustered within 1 cM genetic distance.

The average inter-marker distance estimated (1.138 cM) was lower than that reported in most of the intra- and inter-specific chickpea genetic linkage maps (1.7–8.01 cM)^8,25,29,32,33^ although higher compared with the two latest available high-density inter-specific chickpea genetic maps (0.65 cM,^31^ 0.59 cM^34^) constructed with SSR, SNP and diversity array technology markers. Nevertheless, the new map constructed has an adequate marker map density to be utilized as a reference for construction of an integrated chickpea genetic and physical map,^58^ including identification and mapping of major QTLs regulating important agro-morphological traits in chickpea.

### 3.2. Identification and mapping of QTLs associated with agro-morphological traits

A significant difference of three yield-contributing SW (14–32 g with 87% H^2^), NP (48–142 g with 78% H^2^) and NB (15–31 with 72% H^2^) quantitative traits (Supplementary Table S6) and one abiotic and biotic stress tolerant PH trait in 229 RIL mapping individuals and two parental accessions across 2 yrs based on ANOVA was observed. Bi-directional transgressive segregation of NB and NP beyond that of parental accessions (Supplementary Fig. S4) was observed in RILs. However, the trait segregation of SW in RILs was not beyond that of parental accession ICC 4958. The normal frequency distribution (Supplementary Fig. S4) of NB, NP and SW in RILs and parental accessions indicated the involvement of multiple genes for target trait regulation. It further suggests the utility of developed RIL mapping population (ICC 4958 × ICC 17160) for QTL identification. CV varied from 0.132 for NB to 0.201 in NP. The estimation of correlation coefficient among three pairwise combinations of quantitative traits revealed significant (*P* < 0.001) higher positive correlation between NB and NP (*r* = 0.86) and negative correlation between NP and SW (−0.27) (Supplementary Table S7).

The QTL analysis identified 19 genomic regions harbouring 27 significant QTLs (LOD: 2.0–16.2) associated with SW, NP, NB and PH in chickpea (Fig. 1 and Table 1). The proportion of phenotypic variance explained (PVE) by individual QTLs varied from 1.3 to 31.3% *R*^2^. The average *R*^2^ for all 27 QTLs was 38.7%. Fifteen QTLs revealed major effect on traits individually with *R*^2^ of >10% each. Sixteen QTLs governing multiple traits were mapped on the eight different genomic regions of LGs. The clustering of QTLs associated with multiple quantitative agro-morphological traits (SW, NP and NB) over a single major genomic region possibly suggests pleiotropy and/or local LD (closely linked genes) effects on trait-regulating complex genetic inheritance pattern.^59,60^ The remaining 11 QTLs were only associated with single trait and mapped on 11 different genomic regions of LGs (Fig. 1). Maximum number of QTLs (5 QTLs) were mapped on LG1, followed by LG6 (3 QTLs) and minimum (1 QTL) on LG5. The genomic regions (0.3 cM on LG1 to 7.2 cM on LG4) harbouring the 27 SW, NP, NB and PH QTLs covered by 84 SSR and SNP markers were mapped on eight LGs. Remarkably, 11 major genomic regions harbouring 15 robust QTLs were validated across two geographical locations as well as years/seasons (Fig. 1). These robust QTLs distributed over six LGs (0.3–7.2 cM) showed consistent PVE (10.7–31.3% *R*^2^) at higher LOD (5.2–16.2). Three major genomic regions harbouring three robust QTLs (7.9–16.2 LOD) mapped on LG1, LG4 and LG6 (0.3–7.2 cM) of these were strongly associated (≥20% *R*^2^) with SW and PH (Fig. 1 and Table 1). The 12 genomic regions with 12 QTLs (1.3–9.5% *R*^2^) were either validated in one geographical location/season and/or had <10% *R*^2^.

**Table 1.** Significant QTLs governing four agro-morphological traits identified using a RIL mapping population

LGs	QTLs identity^a^	Marker intervals with genetic positions (cM)	LOD		Phenotypic variation explained (PVE, *R*^2^%) by QTLs		Additive effect	Selected potential TF genes at target QTL intervals
			New Delhi	Palampur	New Delhi	Palampur		
LG1	*qSW1.1*	CaTFSNP4 (9.8)–CaTFSNP6 (12.6)	4.2	2.7	5.9	2.0	2.8	–
	*qNB1.1*	CaTFSNP14 (20.5)–CaTFSNP16 (22.6)	3.3	2.5	6.5	4.2	−2.3	–
	*qSW1.2*	CaSSR27 (37.5)–CaSSR30 (40.2)	10.5	11.2	21.6	20.9	4.1	*DUF1635* *NAC* *SBP*
	*qNB1.2^b^*	CaTFSNP30 (74.5)–CaTFSNP32 (76.2)	9.2	8.5	16.9	17.2	3.7	*bHLH* (basic helix-loop-helix) *MADS* [MCM (mini-chromosome-maintenance) AGAMOUS-DEFICIENS-SRF (serum response factor)]
	*qNP1.1*	CaTFSNP37 (75.9)–CaTFSNP32 (76.2)	8.9	8.3	13. 5	14.4	2.8	
	*qSW1.3*	CaTFSNP38 (115.8)–CaSSR48 (118.9)	5.1	2.8	3.5	1.3	1.4/1.6	AP2-EREBP (ethylene-responsive element-binding protein)
	*qNP1.2*	CaSSR43 (112.6)–CaSSR48 (118.9)	5.4	5.2	11.6	12.4	2.5	*BES1* (bri1-ethyl methanesulfonate suppressor 1)/*BZR1* (brassinazole resistant 1) homolog 4 (*BEH4*)
LG2	*qSW2.1^b^*	CaSSR50 (0.0)–CaSSR53 (3.5)	4.7	3.8	8.9	9.5	4.3	LOB (lateral organ boundaries) domain-containing protein 41 (*LBD41*)
	*qNB2.1*	CaSSR51 (1.8)–CaSSR53 (3.5)	8.2	6.8	16.6	16.2	3.2	
	*qNP2.1*	CaSSR82 (53.9)–CaSSR85 (57.5)	9.7	9.4	13.2	12.4	1.4/1.8	ERF/AP2 (ethylene response factor)
LG3	*qNB3.1*	CaSSR106 (8.8)–CaTFSNP87 (11.7)	4.4	3.7	9.2	8.9	2.4	*ABI3VP1*
	*qSW3.1*	CaTFSNP128 (106.2)–CaSSR154 (109.0)	5.2	3.4	8.1	7.9	1.9	—
	*qNP3.1*	CaSSR153 (107.2)–CaSSR154 (109.0)	5.4	5.3	10.7	11.7	2.1	—
LG4	*qSW4.1^b^*	CaTFSNP164 (35.4)–CaSSR203 (37.4)	9.5	9.8	12.7	13.8	3.8	*bZIP* (basic region leucine zipper) AP2-EREBP *EDA31* (embryo sac development arrest 31)
	*qNP4.1*	CaTFSNP164 (35.4)–CaSSR194 (37.8)	8.8	7.5	15.7	16.3	2.9	
	*qSW4.2*	CaTFSNP248 (150.6)–CaTFSNP203 (156.3)	15.7	16.2	31.3	30.6	5.2	*ABI3VP1* *ARF* (auxin response factor)
	*qNP4.2*	CaTFSNP248 (150.6)–CaSSR250 (157.8)	8.1	7.0	19.9	18.8	2.5/3.1	
LG5	*qSW5.1*	CaTFSNP229 (72.1)–CaTFSNP232 (75.8)	4.6	2.5	2.1	6.6	3.2	—
LG6	*qNB6.1*	CaTFSNP279 (74.1)–CaTFSNP283 (77.7)	9.2	7.8	14.7	16.0	4.5	*NAC*
	*qSW6.1*	CaTFSNP281 (76.0)–CaTFSNP283 (77.7)	4.5	2.7	4.8	4.5	3.6	
	*qNB6.2*	CaSSR381 (120.8)–CaTFSNP298 (123.4)	7.1	6.9	19.9	18.0	3.7	*HAM3* (hairy meristem 3)
	*qPH6.1*	CaSSR381 (120.8)–CaSSR384 (125.6)	8.5	7.9	24.9	29.8	—	
	*qNB6.3*	CaSSR396 (150.8)–CaTFSNP311 (152.6)	4.1	2.6	9.2	9.3	2.1/2.6	C2C2 (coiled-coils)-GATA
LG7	*qNP7.1*	CaTFSNP336 (58.4)–CaTFSNP339 (62.4)	4.7	2.3	7.9	1.5	3.3	—
	*qPH7.1*	CaTFSNP344 (101.4)–CaSSR462 (105.9)	6.0	7.1	20.5	21.9	—	—
LG8	*qNP8.1^b^*	CaSSR466 (8.9)–CaTFSNP359 (12.9)	4.4	2.0	6.1	2.1	2.7	—
	*qNB8.1*	CaTFSNP380 (60.8)–CaSSR491 (64.1)	3.7	3.0	2.0	6.1	3.5	—

^a^*qSW1.1* (QTL for 100-seed weight on chromosome 1 and QTL number 1), *qNB1.1* (QTL for number of branches/plant on chromosome 1 and QTL number 1), *qNP1.1* (QTL for number of pods/plant on chromosome 1 and QTL number 1) and *qPH6.1* (QTL for plant hairiness on chromosome 6 and QTL number 1). CaSSR (*Cicer arietinum* SSR) and CaTFSNP (*Cicer arietinum* transcription factor gene-derived SNP), and details regarding these markers are provided in the Supplementary Tables S3 and S4.

*^b^*Known QTLs from previous studies by Cobos *et al.*,^53,54^ Hossain *et al.*,^55^ Varshney *et al.*,^56^ and Gowda *et al.*,^57^

Considering the individual trait, nine including three major and robust QTLs associated with SW (1.3–31.3% *R*^2^) were identified (Fig. 1 and Table 1). These nine QTLs covered by 38 SSR and SNP markers were mapped on six LGs (1.7 cm on LG6 to 5.7 cM on LG4). All these SW QTLs showed mostly the positive additive gene effects indicating the effective contributions of ICC 4958 alleles at these loci for increasing seed weight (Table 1). For NP, eight including six major and robust QTLs explaining 1.5–19.9% *R*^2^ were identified. These eight QTLs covered by 34 markers were mapped on six LGs (0.3 cM on LG1 to 7.2 cM on LG4) (Fig. 1 and Table 1). These QTLs showed positive additive gene effects for increasing pod number with large allelic contributions either from ICC 4958 or from both ICC 4958 and ICC 17160. For NB, eight including four major and robust QTLs covered by 25 markers were mapped on five LGs (1.7 cM on LG1 and LG2 to 3.6 cM on LG6) with 2.0–19.9% *R*^2^ (Fig. 1 and Table 1). Except all, one NB QTL had negative additive gene effect indicating contributions of ICC 17160 alleles at this loci on decreasing branch number. For PH, two robust and major QTLs mapped on LG6 and LG7 showing 20.5–29.8% *R*^2^ were identified (Fig. 1 and Table 1). These QTL regions (4.5 cM on LG7 to 4.8 cM on LG6) covered by 11 SSR and SNP markers showed positive additive gene effects for increasing hairiness with allelic contributions from ICC 17160. All the three yield-contributing traits (SW, NP and NB) under study showed complex quantitative genetic inheritance patterns in an inter-specific chickpea mapping population. Consequently, it was quite obvious to identify and map multiple robust and major QTLs/genes regulating these traits in chickpea. Although the agro-morphological trait PH appears as a qualitative trait in our study, its phenotype (presence/absence of hairs) is easily affected by environmental cues. This signifies PH as a quantitative trait rather than a qualitative trait. Despite identifying multiple PH-governing loci, this study could detect only two robust QTLs associated with PH. A high-resolution mapping of QTLs controlling PH trait is required for quantitative dissection of this complex trait in chickpea.

To validate the identified QTLs, the genomic regions harbouring SW, NP, NB and PH QTLs were compared with earlier QTL mapping studies (∼50 QTLs controlling diverse agronomic traits) involving different inter-/intra-specific chickpea mapping population.^22,53–57,61–63^ For comparison, the markers linked/flanking these known SW, NP and NB QTLs were genotyped in 229 RIL individuals and parents of an inter-specific mapping population (ICC 4958 × ICC 17160). Four SSR markers (TA110, GAA47, TA72, TS45 and STMS13) showing polymorphism between parents (ICC 4958 and ICC 17160) were genetically mapped into the marker intervals of QTLs (*qSW2.1, qSW4.1, qNP8.1* and *qNB1.2*) on LG2 (TA110), LG4 (GAA47 and TA72), LG8 (TS45) and LG1 (STMS13) as identified in our study. Therefore, two SW and one of each NP and NB QTLs associated with five anchor markers documented by Cobos *et al.*,^53,54^ Hossain *et al.*,^55^ Varshney *et al.*^56^ and Gowda *et al.*^57^ are the same as those identified by our study using a different inter-specific mapping population. Apart from these four known QTLs, none of the identified QTLs showed correspondence with earlier reported QTLs based on their genetic positions on eight chickpea LGs. This indicates that 23 QTLs governing four agro-morphological traits identified by us are novel and showed population-specific genomic distribution in chickpea. Henceforth, these trait-regulating QTLs, once successfully validated in diverse genetic backgrounds, can be utilized for marker-assisted trait improvement of chickpea. The novel as well as robust and major QTLs governing four agro-morphological traits mapped on eight chromosomes were covered by different candidate gene (TFs)-derived SSR and SNP markers (Supplementary Tables S3 and S4). These markers carrying genes including TFs have useful implication for regulating variety of transcriptional activity during developmental process in plants, including *Arabidopsis* and legumes.^64–70^ For instance, the SSR marker (CaSSR381) containing GRAS family TF gene *HAM3* (Hairy meristem 3) found in one strong PH-influencing robust QTL (*qPH6.1*) interval of chickpea supports the previous reports on its unique feature and involvement in differentiation of epidermis with trichomes (enhanced hairs) on the vegetative shoot apical meristem (SAM) of *Arabidopsis*.^71,72^ The development of multicellular glandular plant hairs (trichomes), which usually secrete oxalic acids and malic acids, is known to impart abiotic and biotic stress tolerance in *Cicer* accessions.^73,74^ Henceforth, the *HAM3* TF gene identified in a strong PH-associated QTL region (24.9–29.8% *R*^2^ at 7.9–8.5 LOD) is a potential candidate, which can be transferred to cultivated chickpea for their marker-assisted genetic improvement for abiotic and biotic stress tolerance. One of the strong SW-influencing robust and major QTL region (*qSW1.2*) mapped (20.9–21.6% *R*^2^ at 10.5–11.2 LOD) on LG1 (37.5–40.2 cM) was covered by SSR markers derived from three TF genes, namely *DUF1635* (domain of unknown function), *NAC* (no apical meristem-*Arabidopsis* transcription activation factor-cup shaped cotyledon) and *SBP* (squamosa promoter-binding protein). These marker-associated TF genes underlying the SW QTLs have recently got validated by integrating trait association analysis with QTL mapping (*desi* ICCX-810800 × *kabuli* ICC 20268), transcript profiling and LD-based marker haplotyping, and ascertained as potential candidates for regulating 100-seed weight in chickpea.^36,37^ The validation of these TF gene-derived SSR markers at SW-influencing QTL region in two of our independent studies using two different intra-specific (ICCX-810800 × ICC 20268) and inter-specific (ICC 4958 × ICC 17160) mapping populations suggests the robustness and significance of identified TF genes underlying QTLs for controlling seed weight in chickpea. Henceforth, the genes localized at the trait-regulating QTL intervals, once validated through fine mapping and map-based gene isolation, could act as the potential candidates for marker-assisted genetic enhancement of chickpea.

### 3.3. Target QTL region-specific trait association mapping

**Figure 2.**
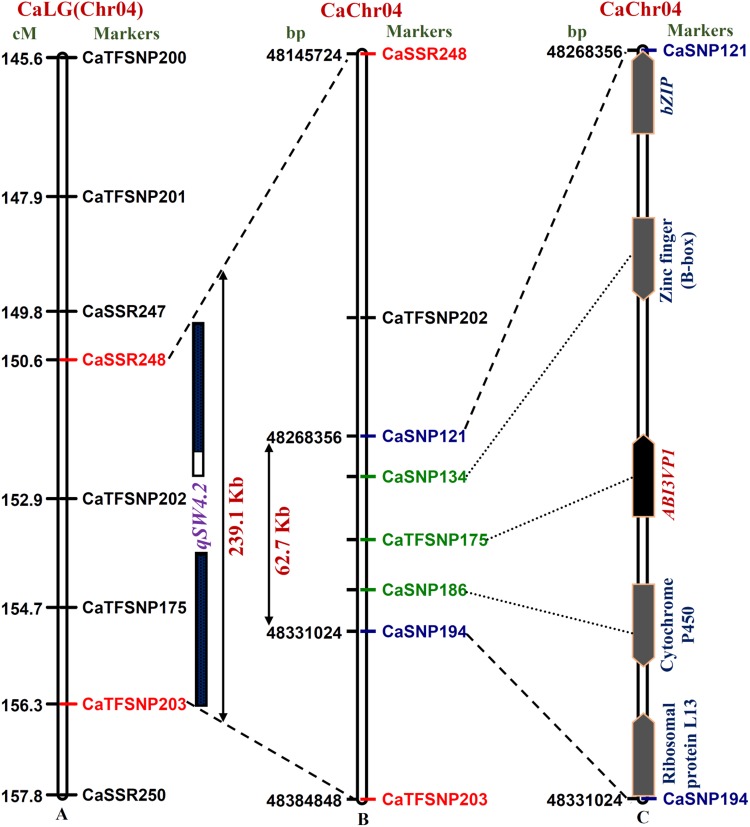
Integration of genetic (A) and physical (B) map of target genomic region harbouring one robust SW-regulating major QTL (*qSW4.2*) identified and mapped QTL on 239.1 kb sequence interval (indicated by red-coloured flanking marker pairs) of chickpea chromosome 4. This QTL was further delimited to an ∼62.7 kb sequenced region on chromosome 4 by integrating traditional QTL mapping with QTL region-specific association analysis (B). Five protein-coding candidate genes annotated within 62.7 kb sequenced interval between markers CaSNP121 and CaSNP194 (marked with blue colour), of which *ABI3VP1* TF gene showed strong association with SW in chickpea (C). The genetic (cM)/physical (bp) distance and identity of the markers mapped on the chromosomes are indicated on the left and right sides of the chromosomes, respectively. The green-coloured markers are derived from the chickpea genes annotated at 62.7 kb QTL (*qSW4.2*) interval. The markers genetically and physically mapped on chickpea chromosome 4 are coloured with black. This figure appears in colour in the online version of *DNA Research*.

To enhance the resolution and fine mapping, one strong (QTL with highest *R*^2^ 30.6–31.3% at 15.7–16.2 LOD) SW-associated major and robust QTL (*qSW4.2*) interval [CaSSR248 (150.6 cM)-CaTFSNP203 (156.3 cM)] was selected based on recombination between two markers and genetic constitution of low and high SW RIL mapping individuals. The major genomic region (48,145.7–48,384.8 kb spanning 239.1 kb on chromosome 4) harbouring such robust QTL was defined (Fig. 2 and Supplementary Table S8) by integrating its genetic linkage map information (recombination of markers in each RILs) with that of physical map of *kabuli* genome (as constructed in our study). This target 239.1 kb *qSW4.2* QTL region was sequenced in low and high seed weight parental accessions (ICC 4958 and ICC 17160) and five of each homozygous individuals of a RIL mapping population using the TruSeq Custom Amplicon v1.5 of Illumina MiSeq next-generation sequencer. The comprehensive sequence analysis at target QTL interval among these 10 RIL mapping individuals and two parental accessions detected 34 SSR and 299 SNP markers (Supplementary Table S8).

The QTL region-specific association analysis by correlating the genotyping information of informative 34 SSR and 192 SNP markers (spanned with an average physical distance of 1.5 kb) in 244 association panel with their SW-specific phenotyping data (100 seed weight: 5.9–57.6 g) was performed. The integration of GLM and MLM analysis and by minimizing the confounding effect of population structure identified four SSR and five SNP markers localized at *qSW4.2* QTL region showing significant association with SW at *P* value ≤10^−3^. The significant percentage contribution of these marker loci to SW trait variation (*R*^2^) varied from 24 to 38.6%. The high-resolution haplotyping among nine SSR and SNP markers with SW enabled to identify three best haplotypes among three SNPs (CaSNP121, CaTFSNP175 and CaSNP194) showing strong association (36.7–47.3% *R*^2^ at 1–3 × 10^−5^ P) with SW in contrast to any other marker combinations. This haplotype region spanned a maximum of 62.7 kb (48268.3–48331.0 kb) physical distance between CaSNP121 and CaSNP194 markers at *qSW4.2* QTL interval.

The structural and functional annotation of this ∼62.7 kb sequenced QTL region with *kabuli* genome annotation database identified five protein-coding candidate genes (Supplementary Table S8; Fig. 2), which are known to be involved in controlling seed development including seed size/weight in crop plants.^37,69,75–78^ The detailed annotation of strong SW-associated haplotypes constituting three SNPs showed their localization in the CDS and URR of three candidate genes, namely *bZIP* (basic leucine zipper domain), *ABI3VP1* (abscisic acid insensitive 3-viviparous 1) and cytochrome P450 localized at *qSW4.2* QTL interval. One regulatory SNP (G/A) in the URR and another non-synonymous SNP (T/C) in the B3 functional domain of *ABI3VP1* TF gene of these revealed strong association (42.5–43.1% *R*^2^ at 1.8–2.0 × 10^−4^ P) with SW. The identification of trait-associated SSR and SNP markers, including non-synonymous SNP loci in different coding/functional domain and URR of three genes suggested their functional significance in establishing marker-trait linkages and identification of genes/QTLs regulating SW trait in chickpea. The expansion/contraction of SSR repeats and non-synonymous substitutions of SNP loci in the functional domain of genes encoding variable amino acid residues might create altered secondary structure of proteins and functional domain regions that possibly affects the DNA binding and transcriptional activity of target gene during seed development. Such possible transcriptional mechanism of trait regulation due to non-synonymous SNP substitutions and SSR repeat-unit variations has already been demonstrated in one of the high seed weight-associated *SBP* TF gene in chickpea^36^ and grain size (*GS3*^79^) and stress-responsive^80^ genes in rice. The expansion and contraction of SSR repeats and SNPs alteration in the URRs of genes also have implications in regulating expression and transcription of genes associated with many agronomic traits.^44,81–83^

### 3.4. Validation of SW-associated genes through expression profiling

To infer the differential expression pattern of five protein-coding genes annotated in the 62.7 kb strong SW-governing robust QTL interval (*qSW4.2*), the gene-based primers were amplified using the RNA isolated from five different vegetative and reproductive tissues and two seed developmental stages of four low and high seed weight chickpea accessions (ICC 4958, ICC 17160, ICCX-810800 and ICC 20268) through semi-quantitative and quantitative RT–PCR assays. Three strong SW-associated genes (*bZIP*, *ABI3VP1* and cytochrome P450) with SNPs (constituting haplotypes) identified based on association analysis of these showed seed-specific expression (Supplementary Fig. S5) compared with vegetative tissues of four accessions analysed. Among these three seed-specific expressed genes, the regulatory and non-synonymous SNPs carrying *ABI3VP1* TF gene constituting strong SW-associated haplotypes at *qSW4.2* QTL interval revealed up-regulated expression (>5-fold) in two seed developmental stages (compared with vegetative tissues) of four low and high seed weight chickpea accessions (Supplementary Fig. S5). We, therefore, selected this *ABI3VP1* TF gene localized at the major and robust SW-governing QTL interval (*qSW4.2*) as target candidate for understanding its significance in seed weight regulation through high-resolution gene haplotype-specific association/LD mapping in chickpea. The narrowing down of acid phosphatase gene-regulating low-phosphorous stress tolerance at major QTL interval by integrating QTL mapping with QTL region-specific association analysis and differential expression profiling has been demonstrated in soybean.^44^

### 3.5. High-resolution haplotype-based LD mapping in a strong SW-associated TF gene

**Figure 3.**
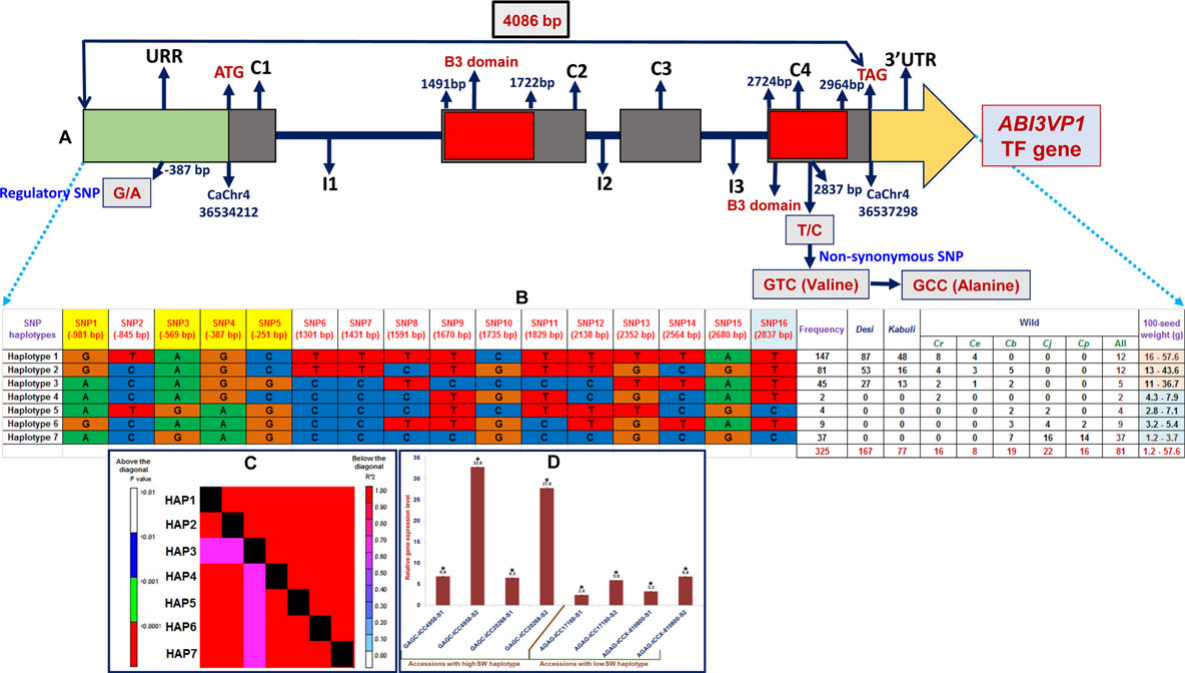
The molecular haplotyping, LD mapping and gene haplotype-specific association analysis in an *ABI3VP1* TF gene validating its strong association potential for SW in chickpea. The genotyping of 16 SNPs including one non-synonymous SNP (T/C) [encoding valine (GTC) to alanine (GCC)] in the B3 functional domain and five regulatory SNPs in the URR of this gene (A) among 244 cultivated and 81 wild chickpea accessions constituted seven haplotypes (B). Thirty-seven low seed weight (1.2–3.7 g) accessions represented by single haplotype group 7 (AGAG) and two other haplotypes (GAGC) consisting 24 accessions of high seed weight (13–57.6 g) in the TF gene showed strong association potential for high and low SW differentiation. The seven SNP haplotype-based genotyping information produced higher LD estimates (*r*^2^ > 0.60 and *P* < 0.0001) covering the entire 4,086 bp sequenced region of gene (C). The high (GAGC) and low (AGAG) seed weight-specific haplotypes constituted by four SNPs (shaded with yellow colour in B) in URR of TF gene are depicted (B). (D) The differential expression profiling of *ABI3VP1* TF gene in high and low seed weight accessions during seed development compared with the leaf. A superior favourable high seed weight-regulating haplotype (GAGC) with increased transcript expression was identified in the URR of TF gene (D). Each bars represent the mean (±standard error) of three independent biological replicates with two technical replicates for each sample used in quantitative RT–PCR assay. *Significant differences in expression of gene haplotypes at two seed developmental stages of low and high seed weight accessions compared with leaf (LSD-ANOVA significance test at *P* < 0.01). This figure appears in colour in the online version of *DNA Research*.

The sequencing of 4,086 bp cloned amplicon covering the whole CDS and 1 kb URR of a strong SW-regulating *ABI3VP1* TF gene (validated by QTL mapping, QTL region-specific association analysis and differential expression profiling) among 244 cultivated (SW-specific association panel) and 81 wild chickpea accessions identified 16 SNP loci (Fig. 3A, Supplementary Table S8). It includes one non-synonymous SNP loci (T/C) [encoding valine (GTC) to alanine (GCC)] in the B3 functional domain and five regulatory SNPs in the URR of this TF gene. The haplotype analysis in *ABI3VP1* TF gene combining the genotyping data of 16 SNPs constituted a maximum of seven haplotypes (with PIC varied from 0.68 to 0.97, mean: 0.63) among accessions (Fig. 3B). All these identified seven haplotypes in TF gene were present in five wild species, whereas three haplotypes were shared particularly by 167 *desi* and 77 *kabuli* chickpea accessions. It thus suggests that ancient human selection has played a major role in evolution of this SW-influencing TF gene during chickpea domestication.

The association analysis using the genotyping data of seven SNP-based haplotypes in the *ABI3VP1* TF gene with SW (1.2–57.6 g)-specific field phenotyping data of 325 chickpea accessions revealed its strong association with SW (46% *R*^2^ at 1.7 × 10^−6^ P). The haplotype-pair-based LD estimation produced a significant high degree of LD (*r*^2^ > 0.60 and *P* < 0.0001) across entire 4,086 bp sequenced TF gene (Fig. 3C), which increased its overall potential for trait association. Such gene haplotype-specific association in presence of high-resolution significant LD have utility to overcome the bi-allelic limitation of SNPs and for improving the efficiency of QTL mapping in crop plants.^44,84–86^ The 37 low seed weight (1.2–3.7 g) accessions represented by single haplotype group 7 (AGAG) in *ABI3VP1* TF gene differentiated distinctly from two other haplotypes (GAGC) consisting 24 accessions of high seed weight (13–57.6 g) (Fig. 3B) with higher phenotypic variance (47–49% *R*^2^ at <1.2 × 10^−5^ P). Therefore, strong association potential of this gene for SW in chickpea is expected. The differential expression profiling using these high (GAGC) and low (AGAG) seed weight-specific haplotypes constituted by four SNPs (−981, −569, −387 and −251 bp) in the URR of an *ABI3VP1* TF gene revealed significant up-regulated expression (average 4-fold), specifically in two seed developmental stages of chickpea accessions compared with leaf (Fig. 3D). It inferred that favourable natural allelic variants and superior haplotype (GAGC) constituted in the URR of an *ABI3VP1* TF gene involved in increased transcript expression and thus have significance in regulation of seed development and consequently seed weight in chickpea. A significant correlation between accessions containing high and low seed weight-specific haplotypes with their diverse transcript expression during seed development and varied seed weight characteristics was evident (Fig. 3D). The high seed weight-specific superior haplotype (GAGC) represented by haplotypes 1 and 2 were present in 228 (70.1%) cultivated *desi* and *kabuli* and wild (*C. reticulatum*, *C. echinospermum* and *C. bijugum*) chickpea accessions, whereas low seed weight-specific haplotype 7 (AGAG) was found exclusively in 37 wild (*C. bijugum*, *C. judaicum* and *C. pinnatifidum*) accessions (Fig. 3). It gave clues for strong selection on seed weight trait among chickpea accessions belonging to primary and secondary gene pools during chickpea breeding. Collectively, our QTL and association mapping gave clues that significant SNP alleles (G/A) and haplotypes (GAGC/AGAG) variations within *ABI3VP1* TF gene underlies the QTL (*qSW4.2*) for seed weight in chickpea. This essentially indicates that these SW-governing alleles of *qSW4.2* is widely distributed among the cultivated and wild chickpea accessions. Nevertheless, having compared our present and past QTL and association mapping reports on given agronomic traits,^22,36,37,53–57,61–63^ only one chickpea accession ICC 4958 was found to be the common parent being specifically utilized for QTL mapping studies. Therefore, most of the SW QTLs (seven QTLs except *qSW2.1* and *qSW4.1*) including *qSW4.2* identified by us are novel and population specific, and hence, they can be utilized for marker-assisted genetic improvement of chickpea for seed weight. Notably, *ABI3VP1* TF gene underlying *qSW4.2* QTL was localized with another QTL *qNP4.2* at the same marker interval [CaTFSNP248 (150.6 cM)-CaSSR250 (157.8 cM)] on LG4. Both these QTLs were found to have positive effects on SW and NP traits with major allelic contribution from the cultivated chickpea. The underlying reason could be the tight linkage of two agronomically important genes and/or pleotropic (multi-functional) effect of *ABI3VP1* TF gene on seed weight and pod number regulation at this target QTL interval.

The SNP marker-based haplotyping and haplotype sharing among accessions overall inferred understanding on possible evolutionary pathway of the *ABI3VP1* TF gene in cultivated and wild chickpea and consequence of its natural allelic/haplotype variation (specifically the superior haplotypes identified in URR of this gene) on seed weight-specific trait evolution during chickpea domestication. Henceforth, the seed weight is supposed to represent an important component of domestication trait in chickpea as documented earlier by Kujur *et al.*^36^ A strong trait association potential (validated by QTL mapping and QTL region-specific association analysis) of *ABI3VP1* TF gene with SW was evident from higher contribution of significant superior haplotypes identified in the URR of this gene with phenotypic variation (∼48%) and their seed-specific expression and pronounced increased transcript expression during seed development. It reemphasizes the functional significance of high seed weight-specific superior haplotype (GAGC) identified in the URR of *ABI3VP1* TF gene for understanding the target trait association and regulation in chickpea. The identified optimal superior haplotype of *ABI3VP1* TF gene will be helpful for accurate selection of chickpea accessions with higher seed weight and also enrich our understanding on gene regulatory networks underlying such complex quantitative seed weight trait in chickpea. The potential role of B3 functional domain containing *ABI3VP1* TF gene as key transcriptional regulator of seed development has been well understood in rice and *Arabidopsis*.^69,87–89^ Therefore, a strong SW-regulating *ABI3VP1* TF gene identified at SW-regulating robust QTL interval by integrating QTL mapping with QTL region-specific association analysis, differential expression profiling and gene haplotype-based association/LD mapping could be a potential candidate for marker-assisted genetic enhancement of chickpea for increasing its seed weight as well as yield.

## 4. Conclusions

A high-density inter-specific genetic linkage map constructed by high-throughput genotyping of genome-wide 834 genic and genomic SSR and SNP markers in our study would expedite genome mapping and targeted mapping of genes/QTLs associated with traits of agricultural importance in chickpea, including comparative mapping across legumes. Eleven novel major genomic regions harbouring 15 robust QTLs (10.7–31.3% *R*^2^ at 5.2–16.2 LOD) associated with three quantitative and one qualitative agro-morphological traits were identified and mapped on eight LGs. Using an integrated genomic approach by combining traditional QTL mapping with QTL region-specific high-resolution association analysis, differential expression profiling and gene haplotype-specific association/LD mapping, we enabled to delineate strong SW-associated favourable natural allelic variants and superior haplotype of *ABI3VP1* TF gene localized at robust SW QTL region. The QTL map-based cloning is a powerful and well-established traditional method of trait-influencing gene/QTL isolation by narrowing down the QTL intervals. However, this approach usually involves excessive time for generation of larger advanced generation mapping population and efficient marker genotyping data for fine mapping the target long QTL regions. In this perspective, an integrated approach developed in our study thus have significance to expedite the process of fine mapping and map-based isolation/positional cloning of genes/QTLs controlling important agronomic traits in chickpea with sub-optimal use of resources. Four agro-morphological trait (SW, NP, NB and PH)-influencing QTL regions are being spanned with various gene (TF)-derived SSR and SNP markers, once validated in diverse genetic backgrounds of chickpea accessions can further be delimited to specific genes/QTLs through integrated genomic approach (developed in our study) or traditional fine mapping/positional cloning. These trait-influencing functionally relevant molecular tags delineated eventually will be used as potential candidates for marker-assisted genetic enhancement of chickpea.

## Supplementary data

Supplementary Data are available at www.dnaresearch.oxfordjournals.org.

## Funding

This study was funded by the Department of Biotechnology (DBT), Government of India, through their research grant (102/IFD/SAN/2161/2013-14). Funding to pay the Open Access publication charges for this article was provided by the National Institute of Plant Genome Research (NIPGR).

## Supplementary Material

Supplementary Data
