# Supplementary material for: An Integrated Genomic Approach for Rapid Delineation of Candidate Genes Regulating Agro-Morphological Traits in Chickpea
Source: DNA Res. 2014 Oct 21;21(6):695–710. doi: 10.1093/dnares/dsu031 (PMC4263302; doi:10.1093/dnares/dsu031)
Supplement: Supplementary Data [file supp_21_6_695__index.html]

An Integrated Genomic Approach for Rapid Delineation of Candidate Genes Regulating Agro-Morphological Traits in Chickpea — Supplementary Data 

# An Integrated Genomic Approach for Rapid Delineation of Candidate Genes Regulating Agro-Morphological Traits in Chickpea

## Supplementary Data

Supplementary Data

**Files in this Data Supplement:**

- Supplementary Data - Pdf file
