# Supplementary material for: An Integrated Genomic Approach for Rapid Delineation of Candidate Genes Regulating Agro-Morphological Traits in Chickpea
Source: DNA Res. 2014 Oct 21;21(6):695–710. doi: 10.1093/dnares/dsu031 (PMC4263302; doi:10.1093/dnares/dsu031)
Supplement: Supplementary Data [file supp_dsu031_dsu031supp.pdf]

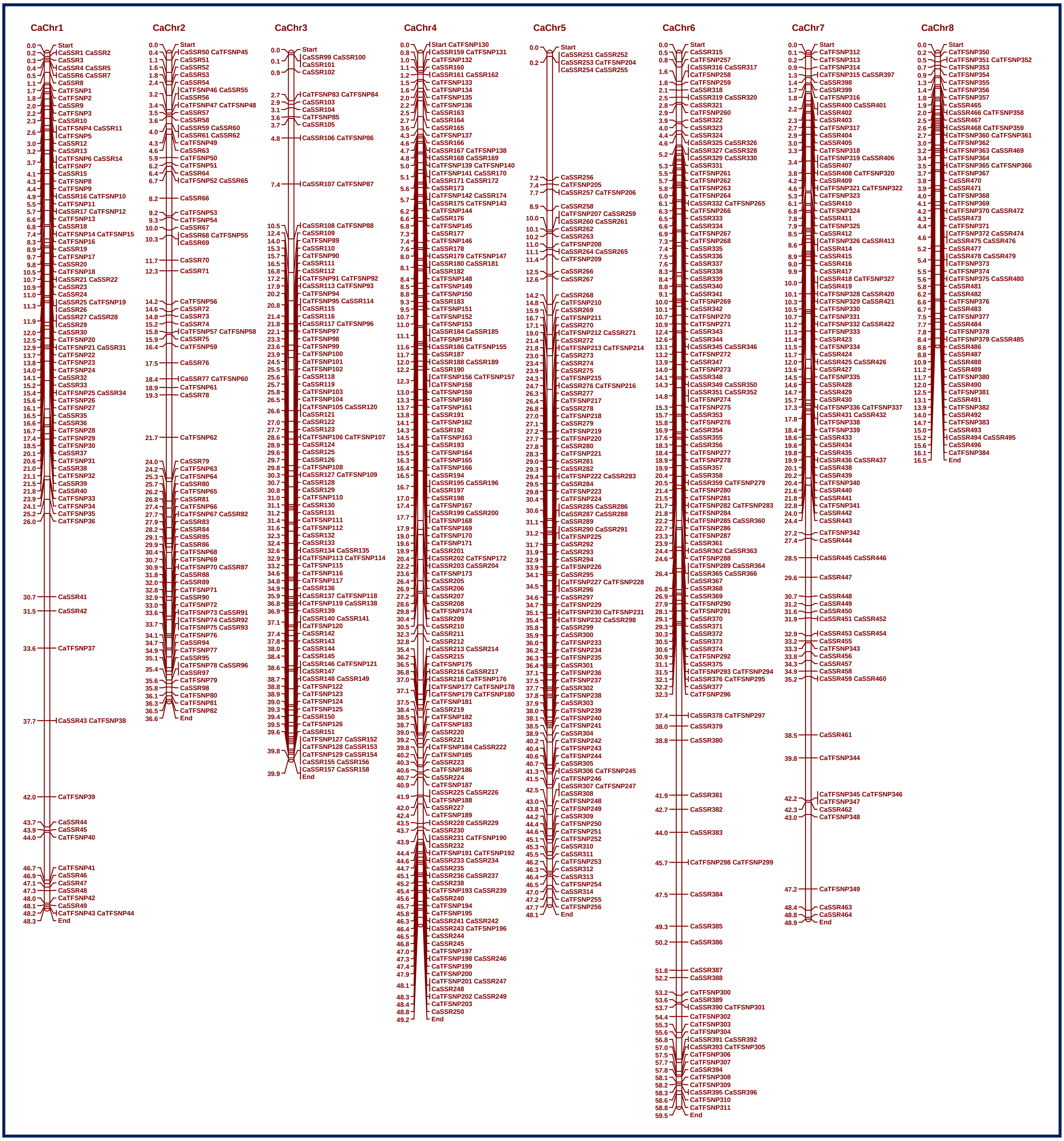

Supplementary Fig. S1: A physical map of chickpea constructed using 880 markers (including 496 genic and genomic SSR markers and 384 TF gene-based SNP markers) showing their genome-wide physical distribution over eight chickpea chromosomes. The physical distance (Mb) and identity of the marker loci integrated on the eight chromosomes are indicated on the left and right side of the chromosomes, respectively. The identity of markers mentioned on the right side of chromosomes that corresponds to the marker IDs “CaSSR” and “CaTFSNP” as mentioned details in the Supplementary Table S3 and S4. The size of the chromosomes (Mb) is based on the draft genome sequence (Pseudomolecule V1.1) of *kabuli* chickpea [Varshney et al. (43)].

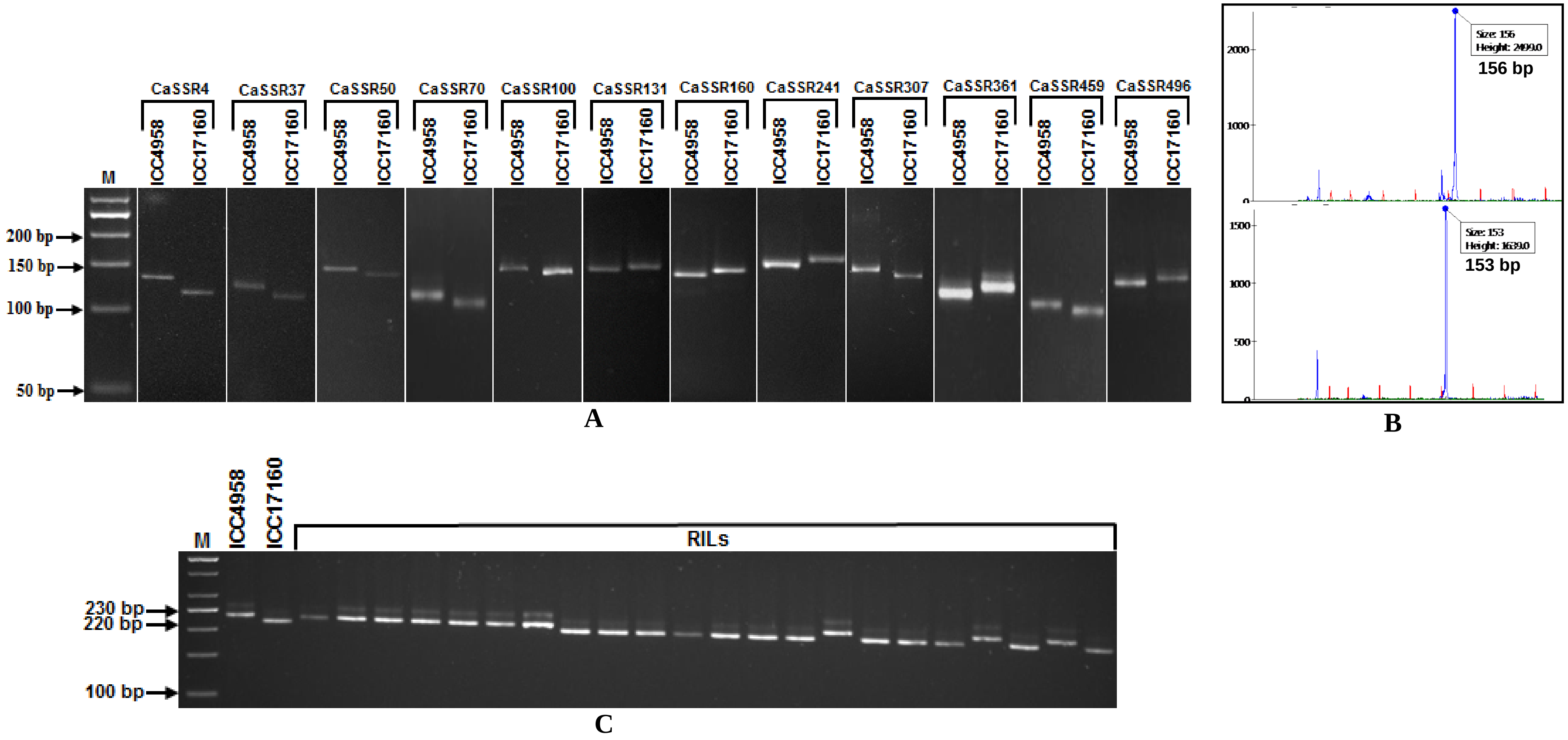

Supplementary Fig. S2: Validation of a representative set of 12 SSR markers (physically mapped across eight chickpea chromosomes) showing *in silico* fragment length polymorphism between parental accessions (ICC 4958 and ICC 17160) of a RIL mapping population (ICC 4958 x ICC 17160) using the gel-based assay (A) and fluorescent-dye labeled automated fragment analyzer (B). (C) The segregation pattern of one selected SSR marker in a representative set of RIL mapping individuals. The fragment sizes (bp) of the amplified polymorphic alleles are indicated. The identities of SSR markers with their detailed information are provided in the Supplementary Table S3. M: 50 bp DNA ladder size standard.

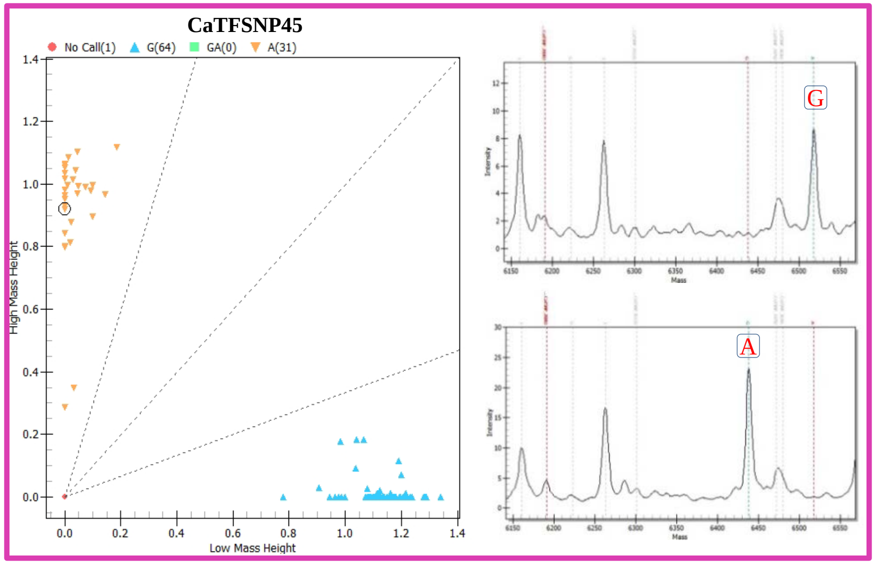

Supplementary Fig. S3: Call cluster plots for one representative SNP (G/A) demonstrating the genotyping information of parental accessions (ICC 4958 and ICC 17160) and RILs assayed with MALDI-TOF mass array. Distinct differentiation of homozygous and heterozygous SNPs based on mass differences of corresponding SNP alleles are evident.

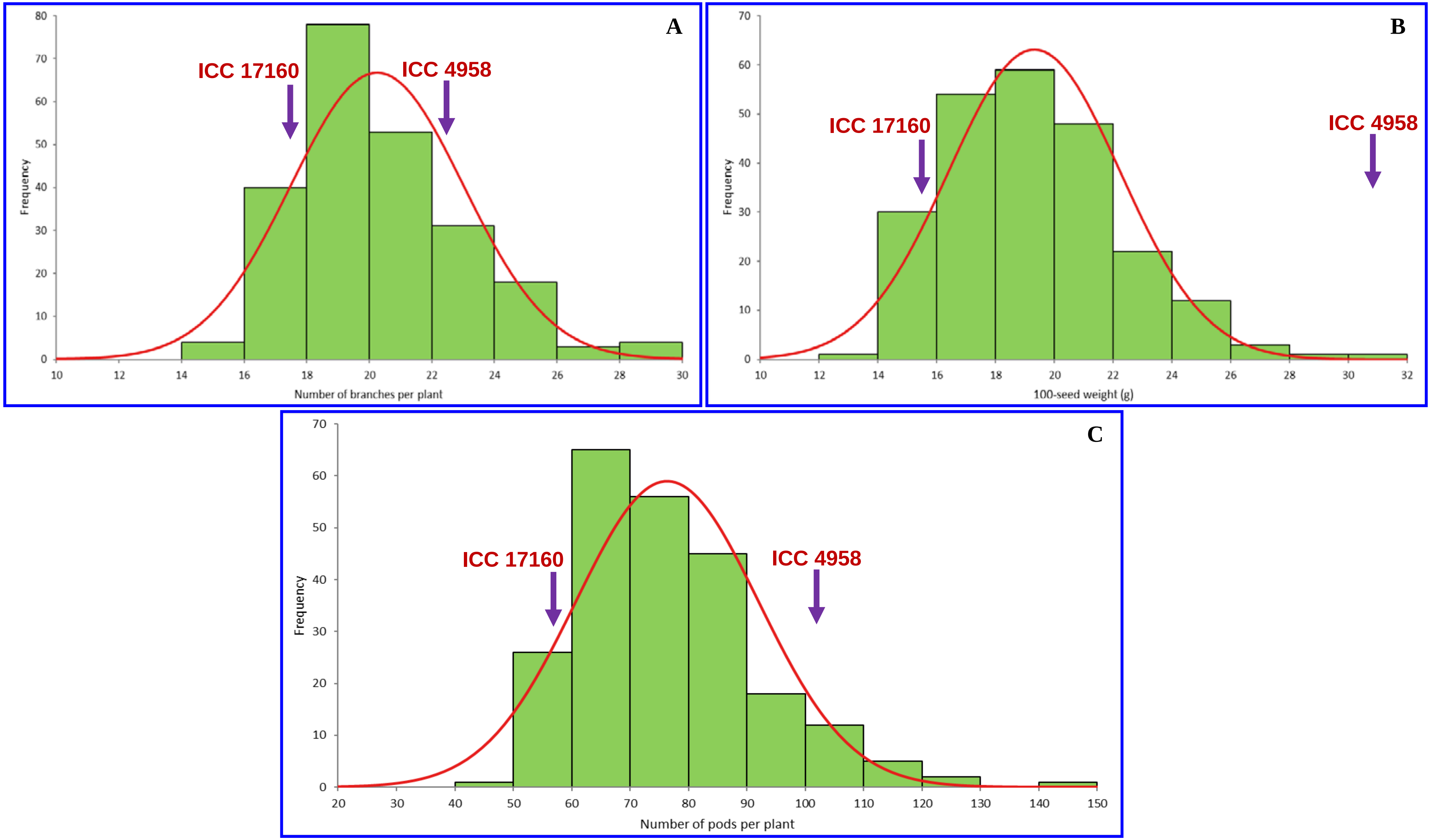

Supplementary Fig. S4: Frequency distribution of three quantitative agronomic traits, namely number of branches per plant (A), 100-seed weight (B) and number of pods per plant (C) in 229 RIL mapping individuals (ICC 4958 x ICC 17160) along with parental accessions depicted a goodness of fit to the normal distribution.

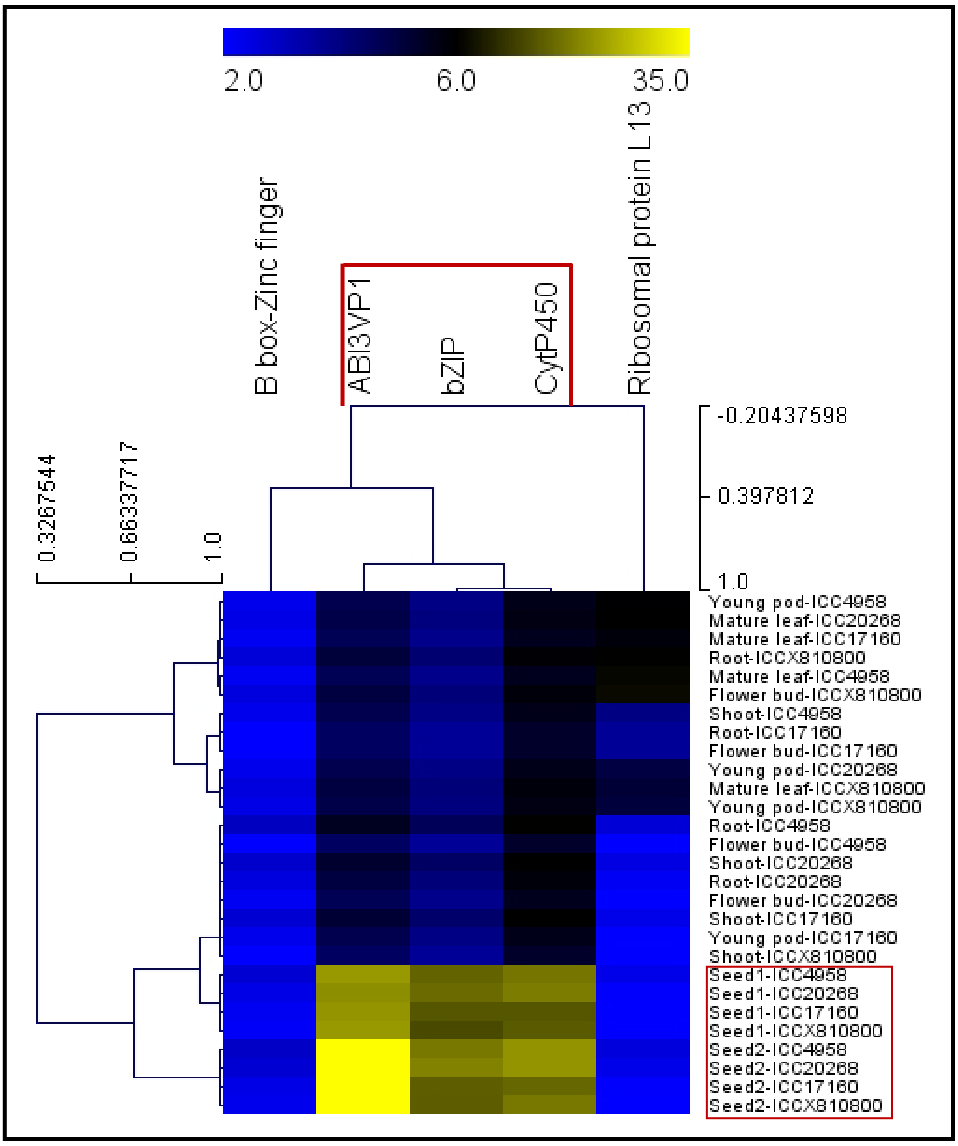

Supplementary Fig. S5: Hierarchical cluster display represented expression profiles for five protein-coding genes differentially expressed in five different vegetative and reproductive tissues and two seed developmental stages of low and high seed weight mapping parents (ICC 4958 and ICC 17160) and contrasting accessions (ICCX-810800 and ICC 20268). The colour scale at the top represents average log signal expression values of genes in various tissues and developmental stages; in which blue, black and yellow color signify low, medium and high level of expression, respectively. The tissues and genes used for expression profiling are mentioned on the right and top side of expression map, respectively. Three seed-specific genes showing higher differential expression in four chickpea accessions during seed development are highlighted with red boxes.

Supplementary Table S1: Chickpea accessions (244 accessions) selected from Kujur et al. (37) for QTL-region specific association mapping

Sl. No.	Accession No.	Cultivars	Geographical origin	Inferred population ancestry
1	ICC3421	*Kabuli*	Israel	*Kabuli* (Population Group I)
2	ICC9402	*Kabuli*	Iran	*Kabuli* (Population Group I)
3	ICC13764	*Kabuli*	Iran	*Kabuli* (Population Group I)
4	ICC12037	*Kabuli*	Mexico	*Kabuli* (Population Group I)
5	ICC12492	*Kabuli*	India	*Kabuli* (Population Group I)
6	ICC13441	*Kabuli*	Iran	*Kabuli* (Population Group I)
7	ICC8855	*Kabuli*	Afghanistan	*Kabuli* (Population Group I)
8	ICC9862	*Kabuli*	Afghanistan	*Kabuli* (Population Group I)
9	ICC13461	*Kabuli*	Iran	*Kabuli* (Population Group I)
10	ICC13628	*Kabuli*	Iran	*Kabuli* (Population Group I)
11	ICC13523	*Kabuli*	Iran	*Kabuli* (Population Group I)
12	ICC13187	*Kabuli*	Iran	*Kabuli* (Population Group I)
13	ICC15802	*Kabuli*	Syrian Arab Republic	*Kabuli* (Population Group I)
14	ICC13077	*Kabuli*	India	*Kabuli* (Population Group I)
15	ICC5337	*Kabuli*	India	*Kabuli* (Population Group I)
16	ICC4841	*Kabuli*	Morocco	*Kabuli* (Population Group I)
17	ICC7571	*Kabuli*	Isreal	*Kabuli* (Population Group I)
18	ICC15264	*Kabuli*	Iran	*Kabuli* (Population Group I)
19	ICC7308	*Kabuli*	Peru	*Kabuli* (Population Group I)
20	ICC2277	*Kabuli*	Iran	*Kabuli* (Population Group I)
21	ICC6263	*Kabuli*	Union of Soviet Socialist Republics	*Kabuli* (Population Group I)
22	ICC8740	*Kabuli*	Afghanistan	*Kabuli* (Population Group I)
23	ICC13357	*Kabuli*	Iran	*Kabuli* (Population Group I)
24	ICC7255	*Kabuli*	India	*Kabuli* (Population Group I)
25	ICC7272	*Kabuli*	Algeria	*Kabuli* (Population Group I)
26	ICC15435	*Kabuli*	Morocco	*Kabuli* (Population Group I)
27	ICC11764	*Kabuli*	Chile	*Kabuli* (Population Group I)
28	ICC15697	*Kabuli*	Syria	*Kabuli* (Population Group I)
29	ICC13816	*Kabuli*	Union of Soviet Socialist Republics	*Kabuli* (Population Group I)
30	ICC7668	*Kabuli*	Union of Soviet Socialist Republics	*Kabuli* (Population Group I)
31	ICC13283	*Kabuli*	Iran	*Kabuli* (Population Group I)
32	ICC7295	*Kabuli*	Tunisia	*Kabuli* (Population Group I)
33	ICC12328	*Kabuli*	Cyprus	*Kabuli* (Population Group I)
34	ICC7315	*Kabuli*	Iran	*Kabuli* (Population Group I)
35	ICC11879	*Kabuli*	Turkey	*Kabuli* (Population Group I)
36	ICC14446	*Kabuli*	Italy	*Kabuli* (Population Group I)
37	ICC10755	*Kabuli*	Turkey	*Kabuli* (Population Group I)
38	ICC14190	*Kabuli*	India	*Kabuli* (Population Group I)
39	ICC15333	*Kabuli*	Iran	*Kabuli* (Population Group I)
40	ICC8042	*Kabuli*	Iran	*Kabuli* (Population Group I)
41	ICC8261	*Kabuli*	Turkey	*Kabuli* (Population Group I)
42	ICC10885	*Kabuli*	Ethiopia	*Kabuli* (Population Group I)

Sl. No.	Accession No.	Cultivars	Geographical origin	Inferred population ancestry
43	ICC8058	*Kabuli*	Iran	*Kabuli* (Population Group I)
44	ICC15512	*Kabuli*	Morocco	*Kabuli* (Population Group I)
45	ICC9137	*Kabuli*	Iran	*Kabuli* (Population Group I)
46	ICC15406	*Kabuli*	Morocco	*Kabuli* (Population Group I)
47	ICC10884	*Kabuli*	Ethiopia	*Kabuli* (Population Group I)
48	ICC15551	*Kabuli*	Australia	*Kabuli* (Population Group I)
49	ICC15725	*Kabuli*	Syrian Arab Republic	*Kabuli* (Population Group I)
50	ICC6204	*Kabuli*	Spain	*Kabuli* (Population Group I)
51	ICC6210	*Kabuli*	Spain	*Kabuli* (Population Group I)
52	ICC11847	*Kabuli*	Chile	*Kabuli* (Population Group I)
53	ICC7654	*Kabuli*	Turkey	*Kabuli* (Population Group I)
54	ICC11749	*Kabuli*	Chile	*Kabuli* (Population Group I)
55	ICC14199	*Kabuli*	Mexico	*Kabuli* (Population Group I)
56	ICC15518	*Kabuli*	Morocco	*Kabuli* (Population Group I)
57	ICC16814	*Kabuli*	Portugal	*Kabuli* (Population Group I)
58	ICC16796	*Kabuli*	Portugal	*Kabuli* (Population Group I)
59	ICC16811	*Kabuli*	India	*Kabuli* (Population Group I)
60	ICC6253	*Kabuli*	Morocco	*Kabuli* (Population Group I)
61	ICC11301	*Kabuli*	USA	*Kabuli* (Population Group I)
62	ICC10749	*Kabuli*	Turkey	*Kabuli* (Population Group I)
63	ICC14216	*Kabuli*	Mexico	*Kabuli* (Population Group I)
64	ICC14203	*Kabuli*	Mexico	*Kabuli* (Population Group I)
65	ICC11742	*Kabuli*	Chile	*Kabuli* (Population Group I)
66	ICC13821	*Kabuli*	Ethiopia	*Kabuli* (Population Group I)
67	ICC14462	*Kabuli*	USA	*Kabuli* (Population Group I)
68	ICC14220	*Kabuli*	Kenya	*Kabuli* (Population Group I)
69	ICC15944	*Kabuli*	USA	*Kabuli* (Population Group I)
70	ICC11303	*Kabuli*	Chile	*Kabuli* (Population Group I)
71	ICC12034	*Kabuli*	Mexico	*Kabuli* (Population Group I)
72	ICC7346	*Kabuli*	Mexico	*Kabuli* (Population Group I)
73	ICC15994	*Kabuli*	Spain	*Kabuli* (Population Group I)
74	ICC18591	*Kabuli*	Mexico	*Kabuli* (Population Group I)
75	ICC8155	*Kabuli*	USA	*Kabuli* (Population Group I)
76	ICC8151	*Kabuli*	USA	*Kabuli* (Population Group I)
77	ICC20268	*Kabuli*	India	*Kabuli* (Population Group I)
78	ICCX-810800	*Desi*	India	*Desi* (Population Group II)
79	ICC5590	*Desi*	India	*Desi* (Population Group II)
80	ICC6013	*Desi*	India	*Desi* (Population Group II)
81	ICC5002	*Desi*	India	*Desi* (Population Group II)
82	ICC3362	*Desi*	Iran	*Desi* (Population Group II)
83	ICC7184	*Desi*	Turkey	*Desi* (Population Group II)
84	ICC3946	*Desi*	Iran	*Desi* (Population Group II)
85	ICC4926	*Desi*	India	*Desi* (Population Group II)
86	ICC12299	*Desi*	Nepal	*Desi* (Population Group II)

Sl. No.	Accession No.	Cultivars	Geographical origin	Inferred population ancestry
87	ICC4657	*Desi*	India	*Desi* (Population Group II)
88	ICC12824	*Desi*	Ethiopia	*Desi* (Population Group II)
89	ICC1052	*Desi*	Pakistan	*Desi* (Population Group II)
90	ICC3761	*Desi*	Iran	*Desi* (Population Group II)
91	ICC5878	*Desi*	India	*Desi* (Population Group II)
92	ICC456	*Desi*	India	*Desi* (Population Group II)
93	ICC5135	*Desi*	India	*Desi* (Population Group II)
94	ICC16207	*Desi*	Myanmar	*Desi* (Population Group II)
95	ICC12537	*Desi*	Ethiopia	*Desi* (Population Group II)
96	ICC12654	*Desi*	Ethiopia	*Desi* (Population Group II)
97	ICC12726	*Desi*	Ethiopia	*Desi* (Population Group II)
98	ICC11944	*Desi*	Nepal	*Desi* (Population Group II)
99	ICC12866	*Desi*	Ethiopia	*Desi* (Population Group II)
100	ICC4814	*Desi*	Iran	*Desi* (Population Group II)
101	ICC6293	*Desi*	Italy	*Desi* (Population Group II)
102	ICC3776	*Desi*	Iran	*Desi* (Population Group II)
103	ICC4418	*Desi*	Iran	*Desi* (Population Group II)
104	ICC4463	*Desi*	Iran	*Desi* (Population Group II)
105	ICC12916	*Desi*	India	*Desi* (Population Group II)
106	ICC11627	*Desi*	India	*Desi* (Population Group II)
107	ICC8607	*Desi*	Ethiopia	*Desi* (Population Group II)
108	ICC11498	*Desi*	India	*Desi* (Population Group II)
109	ICC12928	*Desi*	India	*Desi* (Population Group II)
110	ICC12947	*Desi*	India	*Desi* (Population Group II)
111	ICC4182	*Desi*	Iran	*Desi* (Population Group II)
112	ICC5845	*Desi*	India	*Desi* (Population Group II)
113	ICC9942	*Desi*	India	*Desi* (Population Group II)
114	ICC16261	*Desi*	Malawi	*Desi* (Population Group II)
115	ICC2629	*Desi*	Iran	*Desi* (Population Group II)
116	ICC6571	*Desi*	Iran	*Desi* (Population Group II)
117	ICC762	*Desi*	India	*Desi* (Population Group II)
118	ICC3218	*Desi*	Iran	*Desi* (Population Group II)
119	ICC3325	*Desi*	Cyprus	*Desi* (Population Group II)
120	ICC11584	*Desi*	India	*Desi* (Population Group II)
121	ICC1392	*Desi*	India	*Desi* (Population Group II)
122	ICC3631	*Desi*	Iran	*Desi* (Population Group II)
123	ICC14402	*Desi*	India	*Desi* (Population Group II)
124	ICC2242	*Desi*	India	*Desi* (Population Group II)
125	ICC11664	*Desi*	India	*Desi* (Population Group II)
126	ICC15868	*Desi*	India	*Desi* (Population Group II)
127	ICC1710	*Desi*	India	*Desi* (Population Group II)
128	ICC791	*Desi*	India	*Desi* (Population Group II)
129	ICC2072	*Desi*	India	*Desi* (Population Group II)
130	ICC13524	*Desi*	Iran	*Desi* (Population Group II)

**Sl. No.**	**Accession No.**	**Cultivars**	**Geographical origin**	**Inferred population ancestry**
131	ICC2507	*Desi*	Iran	*Desi* (Population Group II)
132	ICC440	*Desi*	India	*Desi* (Population Group II)
133	ICC2065	*Desi*	India	*Desi* (Population Group II)
134	ICC9586	*Desi*	India	*Desi* (Population Group II)
135	ICC13863	*Desi*	Ethiopia	*Desi* (Population Group II)
136	ICC14077	*Desi*	Ethiopia	*Desi* (Population Group II)
137	ICC16487	*Desi*	Pakistan	*Desi* (Population Group II)
138	ICC1161	*Desi*	Pakistan	*Desi* (Population Group II)
139	ICC6537	*Desi*	Iran	*Desi* (Population Group II)
140	ICC9737	*Desi*	India	*Desi* (Population Group II)
141	ICC2263	*Desi*	Iran	*Desi* (Population Group II)
142	ICC6874	*Desi*	Iran	*Desi* (Population Group II)
143	ICC10945	*Desi*	India	*Desi* (Population Group II)
144	ICC13892	*Desi*	Ethiopia	*Desi* (Population Group II)
145	ICC15618	*Desi*	India	*Desi* (Population Group II)
146	ICC9002	*Desi*	Iran	*Desi* (Population Group II)
147	ICC16269	*Desi*	Malawi	*Desi* (Population Group II)
148	ICC2210	*Desi*	Algeria	*Desi* (Population Group II)
149	ICC14051	*Desi*	Ethiopia	*Desi* (Population Group II)
150	ICC6811	*Desi*	Iran	*Desi* (Population Group II)
151	ICC9643	*Desi*	Afghanistan	*Desi* (Population Group II)
152	ICC12307	*Desi*	Myanmar	*Desi* (Population Group II)
153	ICC14778	*Desi*	India	*Desi* (Population Group II)
154	ICC8195	*Desi*	Pakistan	*Desi* (Population Group II)
155	ICC1715	*Desi*	India	*Desi* (Population Group II)
156	ICC4495	*Desi*	Turkey	*Desi* (Population Group II)
157	ICC11378	*Desi*	India	*Desi* (Population Group II)
158	ICC1431	*Desi*	India	*Desi* (Population Group II)
159	ICC7441	*Desi*	India	*Desi* (Population Group II)
160	ICC12155	*Desi*	Bangladesh	*Desi* (Population Group II)
161	ICC2884	*Desi*	Iran	*Desi* (Population Group II)
162	ICC15606	*Desi*	India	*Desi* (Population Group II)
163	ICC8950	*Desi*	India	*Desi* (Population Group II)
164	ICC12851	*Desi*	Ethiopia	*Desi* (Population Group II)
165	ICC6816	*Desi*	Iran	*Desi* (Population Group II)
166	ICC10393	*Desi*	India	*Desi* (Population Group II)
167	ICC11198	*Desi*	India	*Desi* (Population Group II)
168	ICC6579	*Desi*	Iran	*Desi* (Population Group II)
169	ICC67	*Desi*	India	*Desi* (Population Group II)
170	ICC1923	*Desi*	India	*Desi* (Population Group II)
171	ICC2720	*Desi*	Iran	*Desi* (Population Group II)
172	ICC4567	*Desi*	India	*Desi* (Population Group II)
173	ICC637	*Desi*	India	*Desi* (Population Group II)
174	ICC1882	*Desi*	India	*Desi* (Population Group II)

Sl. No.	Accession No.	Cultivars	Geographical origin	Inferred population ancestry
175	ICC5639	*Desi*	India	*Desi* (Population Group II)
176	ICC8384	*Desi*	India	*Desi* (Population Group II)
177	ICC95	*Desi*	India	*Desi* (Population Group II)
178	ICC1098	*Desi*	Iran	*Desi* (Population Group II)
179	ICC708	*Desi*	India	*Desi* (Population Group II)
180	ICC14098	*Desi*	Ethiopia	*Desi* (Population Group II)
181	ICC1083	*Desi*	Iran	*Desi* (Population Group II)
182	ICC1164	*Desi*	Nigeria	*Desi* (Population Group II)
183	ICC1205	*Desi*	India	*Desi* (Population Group II)
184	ICC14831	*Desi*	India	*Desi* (Population Group II)
185	ICC5434	*Desi*	India	*Desi* (Population Group II)
186	ICC10399	*Desi*	India	*Desi* (Population Group II)
187	ICC4593	*Desi*	India	*Desi* (Population Group II)
188	ICC11121	*Desi*	India	*Desi* (Population Group II)
189	ICC15612	*Desi*	Tanzania	*Desi* (Population Group II)
190	ICC3230	*Desi*	Iran	*Desi* (Population Group II)
191	ICC867	*Desi*	India	*Desi* (Population Group II)
192	ICC16915	*Desi*	India	*Desi* (Population Group II)
193	ICC6802	*Desi*	Iran	*Desi* (Population Group II)
194	ICC14815	*Desi*	India	*Desi* (Population Group II)
195	ICC1356	*Desi*	India	*Desi* (Population Group II)
196	ICC13219	*Desi*	Iran	*Desi* (Population Group II)
197	ICC1422	*Desi*	India	*Desi* (Population Group II)
198	ICC5613	*Desi*	India	*Desi* (Population Group II)
199	ICC14799	*Desi*	India	*Desi* (Population Group II)
200	ICC2969	*Desi*	Iran	*Desi* (Population Group II)
201	ICC1194	*Desi*	India	*Desi* (Population Group II)
202	ICC15567	*Desi*	India	*Desi* (Population Group II)
203	ICC506	*Desi*	India	*Desi* (Population Group II)
204	ICC11284	*Desi*	Union of Soviet Socialist Republics	*Desi* (Population Group II)
205	ICC4639	*Desi*	India	*Desi* (Population Group II)
206	ICC8621	*Desi*	Ethiopia	*Desi* (Population Group II)
207	ICC2580	*Desi*	Iran	*Desi* (Population Group II)
208	ICC283	*Desi*	India	*Desi* (Population Group II)
209	ICC1180	*Desi*	India	*Desi* (Population Group II)
210	ICC9755	*Desi*	Afghanistan	*Desi* (Population Group II)
211	ICC1397	*Desi*	India	*Desi* (Population Group II)
212	ICC14669	*Desi*	India	*Desi* (Population Group II)
213	ICC3512	*Desi*	Iran	*Desi* (Population Group II)
214	ICC1510	*Desi*	India	*Desi* (Population Group II)
215	ICC2990	*Desi*	Iran	*Desi* (Population Group II)
216	ICC7323	*Desi*	Unknown	*Desi* (Population Group II)
217	ICC4533	*Desi*	India	*Desi* (Population Group II)
218	ICC16903	*Desi*	India	*Desi* (Population Group II)

Sl. No.	Accession No.	Cultivars	Geographical origin	Inferred population ancestry
219	ICC7867	*Desi*	Iran	*Desi* (Population Group II)
220	ICC8522	*Desi*	Italy	*Desi* (Population Group II)
221	ICC16374	*Desi*	Malawi	*Desi* (Population Group II)
222	ICC12028	*Desi*	Mexico	*Desi* (Population Group II)
223	ICC1230	*Desi*	India	*Desi* (Population Group II)
224	ICC8318	*Desi*	India	*Desi* (Population Group II)
225	ICC2919	*Desi*	Iran	*Desi* (Population Group II)
226	ICC5504	*Desi*	Mexico	*Desi* (Population Group II)
227	ICC5383	*Desi*	India	*Desi* (Population Group II)
228	ICC15610	*Desi*	India	*Desi* (Population Group II)
229	ICC1398	*Desi*	India	*Desi* (Population Group II)
230	ICC14595	*Desi*	India	*Desi* (Population Group II)
231	ICC13599	*Desi*	Iran	*Desi* (Population Group II)
232	ICC15510	*Desi*	Morocco	*Desi* (Population Group II)
233	ICC7554	*Desi*	Iran	*Desi* (Population Group II)
234	ICC15294	*Desi*	Iran	*Desi* (Population Group II)
235	ICC4918	*Desi*	India	*Desi* (Population Group II)
236	ICC7819	*Desi*	Iran	*Desi* (Population Group II)
237	ICC1915	*Desi*	India	*Desi* (Population Group II)
238	ICC6279	*Desi*	India	*Desi* (Population Group II)
239	ICC6306	*Desi*	Union of Soviet Socialist Republics	*Desi* (Population Group II)
240	ICC16524	*Desi*	Pakistan	*Desi* (Population Group II)
241	ICC6877	*Desi*	Iran	*Desi* (Population Group II)
242	ICC1836	*Desi*	India	*Desi* (Population Group II)
243	ICC15061	*Desi*	India	*Desi* (Population Group II)
244	ICC13124	*Desi*	India	*Desi* (Population Group II)

Supplementary Table S2: Chickpea accessions (81 accessions) selected from five wild species for gene haplotype-based LD mapping

Sl. No.	*Cicer* species	Accession No.	Cultivar types	Geographical origin
1	***C. reticulatum***	ICC17160	Wild	Turkey
2		ILWC290	Wild	Turkey
3		ILWC233	Wild	Turkey
4		ILWC253	Wild	Turkey
5		ILWC254	Wild	Turkey
6		ILWC237	Wild	Turkey
7		ILWC257	Wild	Turkey
8		ILWC218	Wild	Turkey
9		ILWC247	Wild	Turkey
10		ILWC229	Wild	Turkey
11		ILWC219	Wild	Turkey
12		ILWC242	Wild	Turkey
13		ILWC258	Wild	Turkey
14		ILWC21	Wild	Turkey
15		ILWC36	Wild	Turkey
16		ILWC216	Wild	Turkey
17	***C. echinospermum***	ICC17159	Wild	Turkey
18		ILWC35	Wild	Turkey
19		IG135418	Wild	Syrian Arab Republic
20		ILWC239	Wild	Turkey
21		ILWC245	Wild	Turkey
22		ILWC238	Wild	Turkey
23		ILWC246	Wild	Turkey
24		ILWC288	Wild	Turkey
25	***C. judaicum***	ICC182	Wild	ICRISAT, India
26		ICC17150	Wild	Lebanon
27		ILWC95	Wild	ICRISAT, India
28		ILWC31	Wild	Jordan
29		ILWC185	Wild	ICRISAT, India
30		ICC17148	Wild	Lebanon
31		ILWC280	Wild	Syrian Arab Republic
32		ILWC283	Wild	Syrian Arab Republic
33		ILWC20	Wild	Israel
34		ILWC211	Wild	Syrian Arab Republic
35		ILWC30	Wild	Israel
36		ILWC48	Wild	Syrian Arab Republic
37		ILWC50	Wild	Syrian Arab Republic
38		ILWC207	Wild	Syrian Arab Republic
39		ILWC45	Wild	Syrian Arab Republic
40		ILWC38	Wild	Lebanon
41		ILWC278	Wild	Syrian Arab Republic

Sl. No.	*Cicer* species	Accession No.	Cultivar types	Geographical origin
42		ILWC275	Wild	Lebanon
43		ILWC223	Wild	Lebanon
44		ILWC256	Wild	Jordan
45		ILWC273	Wild	Lebanon
46		ILWC4	Wild	Lebanon
47	***C. bijugum***	ILWC42	Wild	Syrian Arab Republic
48		IG136792	Wild	Syrian Arab Republic
49		IG136786	Wild	Syrian Arab Republic
50		ILWC277	Wild	Syrian Arab Republic
51		ILWC8	Wild	Turkey
52		ILWC228	Wild	Turkey
53		ILWC240	Wild	Turkey
54		ILWC241	Wild	Turkey
55		ILWC260	Wild	Turkey
56		ILWC217	Wild	Turkey
57		ILWC209	Wild	Syrian Arab Republic
58		ILWC227	Wild	Turkey
59		ILWC284	Wild	Turkey
60		ILWC7	Wild	Turkey
61		ILWC220	Wild	Turkey
62		IG136796	Wild	Syrian Arab Republic
63		ILWC285	Wild	Turkey
64		ILWC286	Wild	Turkey
65		ILWC32	Wild	Turkey
66	***C. pinnatifidum***	ILWC9	Wild	Turkey
67		ILWC22	Wild	Turkey
68		IG136820	Wild	Syrian Arab Republic
69		ILWC251	Wild	Turkey
70		ILWC29	Wild	Turkey
71		ILWC33	Wild	Turkey
72		ILWC49	Wild	Syrian Arab Republic
73		ILWC226	Wild	Turkey
74		ILWC248	Wild	Turkey
75		ILWC249	Wild	Turkey
76		ILWC236	Wild	Turkey
77		ILWC225	Wild	Turkey
78		ILWC51	Wild	Turkey
79		ILWC250	Wild	Turkey
80		ILWC261	Wild	Syrian Arab Republic
81		ILWC289	Wild	Turkey

Supplementary Table S3: Summary of 496 genic and genomic SSR markers used for polymorphism survey between parental accessions (ICC 4958 and ICC 17160) of RIL mapping population employing gel-based assay and fluroscent dye-labelled automated fragment analyser

Marker IDs*	Microsatellite repeat-motifs	Forward Primer sequences (5'-3')	Reverse Primer sequences (5'-3')	Actual annealing temperature (0C)	Size (bp) of alleles amplified	Types of markers	Chromosomes	Physical positions (bp)	Putative functions
CaSSR1	(TTA)5	CCTCGCCAAATAATCTCAGG	CCGAAGAGCAGAGGAAGATG	60.1	259	Genic	Ca1	203295	Zinc finger C-x8-C-x5-C-x3-H type
CaSSR2	(TTA)8	TGATGATTATTTCGTTTCCTC	GCAACTAGAAGACGCTGATAA	54.4	152	Genic	Ca1	203312	Zinc finger C-x8-C-x5-C-x3-H type family protein
CaSSR3	(TCT)7	ACGGTCAGTGAGCTGCTTCT	TCCCAATCCTAGCTACCACG	60.1	151	Genic	Ca1	317669	Pseudouridine synthase family protein
CaSSR4	(TCTCAT)4	GGTGCTACTGATTTCAATGTT	AGGAGCATCTTCTTCACTTTC	54.4	156	Genic	Ca1	389097	TCP family transcription factor
CaSSR5	(ATTCTC)5	CAACAACAACAACAAGAATCA	GTTTGAAGAAGGTTGTTGGAT	54.7	153	Genic	Ca1	390435	TCP family transcription factor
CaSSR6	(GA)11	TCAAAGAACCAAAATTCGAACA	CGGTTGATTGATAATTCTCGTG	59.6	153	Genomic	Ca1	472339	
CaSSR7	(CAT)5	AACCTCTTCTTTCACACCTTGC	GGGGATATGATCCAGCCTTT	60.1	279	Genic	Ca1	505905	KT2, a photosynthate- and light-dependent inward rectifying potassium channel
CaSSR8	(ATAAA)9	TGCTGCCAACAAACTTAGCA	GGCACCTTTCCTTGTACTGG	60.6	202	Genomic	Ca1	1145423	
CaSSR9	(AT)8	GGCTGTGTTTGGTTGTGTTG	TTGCATGCTTTTACCAAACC	58.7	180	Genic	Ca1	2046119	Putative secretory carrier membrane protein (SC3).
CaSSR10	(AAC)6	TGGGTTTGAGTTCTAAACAAG	CATTTCACAGCTTCTGATTGT	54.6	151	Genic	Ca1	2346126	Dof zinc finger protein adof2.
CaSSR11	(GAG)6	GGAAGCAGAAACAGTAGAGGT	TGTTCTTCCTCCTCTTTAACC	55.3	165	Genic	Ca1	2631745	zinc finger domain containing protein
CaSSR12	(TCT)6	GAAGCATCGTCTTCCCTGAG	AATCGTTGGCGTTGTTTCTC	60.1	192	Genic	Ca1	2994601	RNA helicase family protein
CaSSR13	(GGAAGT)3	ATAATGGTTACTTTCGGAGGA	TCTTCTTCGTGTTCATGTTCT	55.4	151	Genic	Ca1	3235738	sequence-specific DNA binding transcription factors
CaSSR14	(AT)9	TTGAACCCCAAATTCTCAGC	AAAGATGGTCCATAGTGAAGCAC	60.1	237	Genomic	Ca1	3688143	
CaSSR15	(GA)18	GTTAGGGGCCCATTTTGAAC	GATGGCTTCACGACCTTGTT	60.5	210	Genomic	Ca1	4097905	
CaSSR16	(CTGATT)4	CGCAGATTATATCTCACGGTA	CTATACCGTTTCAAATCCAAA	55.6	148	Genic	Ca1	4865629	BSD domain-containing protein
CaSSR17	(TTG)4	AAAAACAAGGAACCAAAGAAG	ATCTCCTCTATCATTGGTGGT	55.3	126	Genic	Ca1	5666044	nuclear coiled-coil protein related to the carrot peripheral nuclear protein NMCP1
CaSSR18	(TCT)6	TACCCAGATGAGATCTACGAA	GGATGGTATCACTGTTTGTGT	54.9	152	Genic	Ca1	6750597	myb-like HTH transcriptional regulator family protein
CaSSR19	(AT)14	CACATTTGTCATTTTGtAACAGCA	TGAGTTGGGAGGGGAGACTA	60.0	259	Genomic	Ca1	8867063	
CaSSR20	(ATG)5	AATTCAGGTCCTACAAAATCC	GAGCAGTGTGCCTATGATATT	54.8	151	Genic	Ca1	9791574	Glabra 2, a homeodomain protein
CaSSR21	(TCATGA)4	ATCATCATGAAGCATCCATAG	TATCGGATATAGCTTTTGCAC	55.1	156	Genic	Ca1	10682877	Involved in radial organization of the root and shoot axial organs
CaSSR22	(TCATGA)4	ATCATCATGAAGCATCCATAG	TATCGGATATAGCTTTTGCAC	55.1	156	Genic	Ca1	10682877	Involved in radial organization of the root and shoot axial organs
CaSSR23	(AATAA)4	CCTTTTTGGGTTCTTATGATT	ATTACACGAAGGATGAATTTG	55.0	151	Genic	Ca1	10893797	putative transcription factor
CaSSR24	(CAA)6	CCTTCACCTATCCCACTGGA	TGGGCCAAAGTGGATAAGAC	59.9	249	Genic	Ca1	11011602	Protein kinase superfamily protein
CaSSR25	(AT)11	AATCATAACCGATAACAAAACTCAT	TCATTAGACTCCAACAATTCATAGAC	57.3	268	Genomic	Ca1	11261320	
CaSSR26	(TAT)8	AGAAGAATCCAAATCCAAAAC	GAAGCCTCTTTCTCCTGATAC	55.0	148	Genic	Ca1	11285919	GRAS family Protein, transcription factor
CaSSR27	(CAA)6	CCACCTGTTACTATTCCTTCA	AAGGAGAATCAGAACCTTCAC	55	151	Genic	Ca1	11897074	DUF1635
CaSSR28	(GAT)6	ATGAGTCAAAGCCATAGTCAC	TTCTTCTTCAGTTGGATGAAA	55	153	Genic	Ca1	11899747	NAC
CaSSR29	(CTC)4	CTAAAGAATGGAATTGGGATT	CTCGTTTGTTTGCTCTATTGT	55	150	Genic	Ca1	11902768	SBP
CaSSR30	(AT)12	GTTGGTGCCAACATGCCTAT	TGAACCGTGtTTCaAAACCT	60.8	163	Genomic	Ca1	11960068	
CaSSR31	(CTT)9	TAAGTTGTGGCTGTGAGAGAT	GCATGAAAATTCAGAGAGAAA	55.0	157	Genic	Ca1	12928971	GLK1, Golden2-like 1
CaSSR32	(GAA)9	TGAGGATTCAAATGTTAAGGGG	TTCAGCAAGCAATGCAAATC	60.0	255	Genic	Ca1	14140590	Homeodomain-related
CaSSR33	(AAC)5	TCCTTTTGGTGACACTACAAATACA	AAAAAGAGACGCCATTCACG	60.2	277	Genic	Ca1	15231892	Eukaryotic aspartyl protease family protein
CaSSR34	(AAT)7	ACCTTATCAATGATTGCTTCA	CAAGACAAAGAGGAAATTGTG	54.9	154	Genic	Ca1	15378933	putative transcription factor
CaSSR35	(AG)10	GTTCCGAATTTGCGATCACT	TTTGCCGTTCATGGTGATAA	60.1	200	Genomic	Ca1	16531210	
CaSSR36	(AG)13	GAACTGAGCGAGGAAGTTGG	ACATCTCCGAACTCGACCAC	60.1	165	Genic	Ca1	16618090	putative TRAPPII tethering factor

Marker IDs*	Microsatellite repeat-motifs	Forward Primer sequences (5'-3')	Reverse Primer sequences (5'-3')	Actual annealing temperature (0C)	Size (bp) of alleles amplified	Types of markers	Chromosomes	Physical positions (bp)	Putative functions
CaSSR37	(TAA)7	TGAGATAATGGAGCTTGAATC	AGCTTGTTGAAGTGACACAGT	54.5	152	Genic	Ca1	20106435	basic helix-loop-helix (bHLH) DNA-binding superfamily protein
CaSSR38	(AAG)5	TTTCCTCACTTCAAAGACCCA	ATTTGACGAAACTCCAACGG	60.0	252	Genic	Ca1	21017902	IQ-domain 22 (IQD22)
CaSSR39	(ATC)5	AAGACCAATCATCAAACCGAA	AAGCTTGTGCAGGGAACACT	59.9	172	Genic	Ca1	21506732	YABBY family of transcriptional regulators
CaSSR40	(CT)12	GCGATCTCTCGAAAACCCTA	GAACGCAAACCACATGATTG	60.0	159	Genic	Ca1	21752019	ENSANGP00000022085 related
CaSSR41	(TA)12	TGAGAGAGGAAAAaCaGATTGAGA	CACGGATCTTGTGAGTGACC	59.5	141	Genomic	Ca1	30671033	
CaSSR42	(TCT)10	GCCATCTTCTACTTCCGCAC	AATTCGAACTCACCACTCGG	60.1	231	Genic	Ca1	31527936	putative protease SppA (SppA).
CaSSR43	(TC)7	TGAGAACAAGACCAGAGTCAC	GCATTTGTTCTTGAATCTCTC	55.3	144	Genic	Ca1	37689960	Possible function in phloem development in the root
CaSSR44	(CT)8	CCTCTTCAAACCATCCTCACA	TCTCGAGCGATCCATCTTTT	59.9	201	Genic	Ca1	43729171	Trypsin family protein
CaSSR45	(GGA)7	AGAGAAGAGAGAGGGGTCGG	CGACTTCGAGTTCCGTCTTC	60.0	184	Genic	Ca1	43894448	TT_ORF1, TT viral orf 1
CaSSR46	(AC)6cct(C)15	AAACGGATCAATGCGAGAAG	CATTACATTGTCATTTGGCCC	60.2	279	Genomic	Ca1	46935324	
CaSSR47	(AT)8	CTACCTTATGGGCACGCAAT	AGCCAATTTGGTGTTgGAAG	60.0	265	Genomic	Ca1	47131694	
CaSSR48	(ATTTT)4	AGGAATTAAGGTGATGAAAGC	GTTTAACAAGACGGACCAAAT	55.2	164	Genic	Ca1	47290885	BES1/BZR1 homolog 4 (BEH4)
CaSSR49	(TAA)7	CATGGAATTCCGGTGCTACT	GACGGTGTAGCTGCATGAAA	59.9	112	Genic	Ca1	48129325	TCP family transcription factor
CaSSR50	(GA)13	ATGGAGGCGAATATATAGGAG	TTTTAAGAAACAACTGCGTTC	55.1	151	Genic	Ca2	354109	LOB domain-containing protein 41 (LBD41)
CaSSR51	(GAT)6	GGAATTTTGGTGATGATTTTC	CAACATATGAAGGAACACACA	56.0	149	Genic	Ca2	1133300	WRKY Transcription Factor
CaSSR52	(TTC)11	GTCCCCCGCAGTTACTGTTA	GTAATTGTGAAGCCGGTCGT	60.0	218	Genic	Ca2	1588490	Protein of unknown function (DUF3531)
CaSSR53	(AC)6(A)10	TTGTTTCTCCAAACCAACTACA	TGCTTCCATAAGTTCTCCCG	57.3	119	Genomic	Ca2	1754600	
CaSSR54	(AG)11	TGAGAAAAATTAGCACACAAAG	ACATATTCATGCTGTTGTTCC	55.0	143	Genic	Ca2	2409848	homeodomain leucine zipper class I (HD-Zip I) transcriptional activator
CaSSR55	(ATG)5	TTGAAGGTGGTGATGGTGAA	GGGCACCTTATTAGCCCATT	60.2	169	Genic	Ca2	3226335	Glutaredoxin family protein
CaSSR56	(TTAA)3	CATCTATAAATGTGGCACAAG	GATGGAGTGAAGTTTGAGATG	53.4	128	Genic	Ca2	3236381	PRE1 (PACLOBUTRAZOL RESISTANCE1)
CaSSR57	(CACAAA)4	GGAGGTTATAACAACACCAAC	GGCTTTGTTATGTTTGAGGTT	53.7	150	Genic	Ca2	3451762	Mitochondrial substrate carrier family protein
CaSSR58	(TGT)5	CAGGAACCAAGATTGCAAGA	GGGAAAGAGTTCAAAACCCA	59.0	192	Genic	Ca2	3632977	SPX domain gene 4 (SPX4)
CaSSR59	(TCA)7	CAACCAAAAAGACAAGAATGA	GGCTCTTGATACCCTTACAAT	55.5	160	Genic	Ca2	4036035	a chloroplast trans-acting factor of the psbD light-responsive promoter
CaSSR60	(TCA)7	CAACCAAAAAGACAAGAATGA	GGCTCTTGATACCCTTACAAT	55.5	160	Genic	Ca2	4036035	a chloroplast trans-acting factor of the psbD light-responsive promoter
CaSSR61	(TGA)7	GGCTCTTGATACCCTTACAAT	CAACCAAAAAGACAAGAATGA	55.0	160	Genic	Ca2	4036194	a chloroplast trans-acting factor of the psbD light-responsive promoter
CaSSR62	(TAA)15	TAAAATCCTCCTCCACACCG	GGATGGACACATACATgGGG	59.9	169	Genomic	Ca2	4037173	
CaSSR63	(GA)14	GCAAAAGACACATTTAGGGTTT	GTAGGTGCAGGCCCTACCTT	57.4	270	Genomic	Ca2	4570670	
CaSSR64	(CCA)11	ACATTGTTGGTCGTGTTTGC	GGGTGATGTTGGAAGGATGA	60.7	259	Genic	Ca2	6404844	Core-2/I-branching beta-1,6-N-acetylglucosaminyltransferase family protein
CaSSR65	(TA)11	GAACCCCTTTcAACAAACGA	CGAGCCTCGTAAAATCCAAA	59.9	158	Genomic	Ca2	6749922	
CaSSR66	(AT)11	CCCTTCACTTTCTCACATAAAGG	AAGCCCTGTAACTCCAGTCG	59.2	195	Genomic	Ca2	8249226	
CaSSR67	(CTT)8	TCCTCCAACAACAACACCAA	AGGAGGAACCTTTGAAACCC	59.4	218	Genic	Ca2	9985718	Nucleic acid-binding proteins superfamily
CaSSR68	(TTC)6	AATGCCTTCACGAATTTTGG	AGGTTACTTCCTGCGGGTTT	60.0	240	Genic	Ca2	10263909	Expressed protein
CaSSR69	(AG)12	TTCCAGATCTCCGGTAGGTG	ACTCTCCACTCTCCCAACCA	59.7	209	Genic	Ca2	10330416	A basic helix-loop-helix encoding gene (BIGPETAL, BPE)
CaSSR70	(ATC)4	AGCAAATTTCATGCTACAAAC	TCCTAGTTCAATTCCTGATGA	54.8	151	Genic	Ca2	11661488	homeodomain leucine zipper class I (HD-Zip I) protein.
CaSSR71	(TC)10	TGGATCCATTTGGTTtGGAT	TTCTTTCTCGTCGACTCCGT	60.0	226	Genomic	Ca2	12299937	

Marker IDs*	Microsatellite repeat-motifs	Forward Primer sequences (5'-3')	Reverse Primer sequences (5'-3')	Actual annealing temperature (0C)	Size (bp) of alleles amplified	Types of markers	Chromosomes	Physical positions (bp)	Putative functions
CaSSR72	(GCA)4	CTTTAATCATGCCAGCTCTAC	TCCCTTTCTCTTTCTCTTTTC	54.4	165	Genic	Ca2	14624000	AL4, Alfin-Like family protein containing PhD domain
CaSSR73	(TA)10	TGGATGAGCCcTTCTTGAAC	TGAAATTTAAATGGGTGCCA	60.2	182	Genomic	Ca2	14846317	
CaSSR74	(ATG)9	CAGGCCTTGTTTGTTGAGGT	TTCTCCTCGATTTCAATGGG	60.0	206	Genic	Ca2	15235771	ABI five binding protein (AFP1)
CaSSR75	(TG)7(TA)8	TCGGAAAATTGAGAAAGTCTTCA	GCAAATGCTCCCCTAATTGA	60.2	276	Genomic	Ca2	15920661	
CaSSR76	(AT)10	TGACCATTTGCATTAGTTCTCAA	GCCACACCACtTTCAtTTTTC	59.6	246	Genomic	Ca2	17520553	
CaSSR77	(CT)8ttc(T)10	TCCGTTTAATTTTGCTTGCT	CGTGCACTTCAAAACCATGA	57.6	248	Genomic	Ca2	18353310	
CaSSR78	(ACA)8	CTCAAACTCCTCCACGAAGC	TAAACACCCATCTTGTGCCA	60.0	270	Genomic	Ca2	19292063	
CaSSR79	(TGA)4	TGTCTCATCTGAAAAAGATTGA	TTGGTTTCTTCATCAACAACT	55.1	150	Genic	Ca2	23951109	auxin (indole-3-acetic acid) induced gene
CaSSR80	(TTA)7	AAGATTTACCGCATCACATATCTC	CCTCCTACCCCACATTACCA	58.6	203	Genomic	Ca2	25670979	
CaSSR81	(CAA)4	AAAACAACAACTCTTCAATGC	TCTCCAATTGTAACCATCATC	54.6	155	Genic	Ca2	26834137	plant WRKY transcription factor
CaSSR82	(TC)12	CATCTCTCTCACTCTCTTTCC	AGATATGGGTAGCAAACGAAC	53.2	200	Genic	Ca2	27694606	ERF (ethylene response factor) subfamily B-5 of ERF/AP2 transcription factor family
CaSSR83	(TGG)6	TCTAGGCCTGGAAAAAATGGTT	TGATTCCCATTTCCTCTTGC	60.0	179	Genic	Ca2	27874806	Expressed protein
CaSSR84	(TTC)5	TCTTTCGCTTTCACTCCCTC	GGTGGAAGCGGTTATTGAGA	60.1	227	Genic	Ca2	28230844	Concanavalin A-like lectin Putative protein kinase
CaSSR85	(AT)16	TTGTCACCATTATCTCCACTC	CCAATATCTATTCATCTTCACCT	55.1	144	Genic	Ca2	29098865	protein disulfide isomerase-like (PDIL) protein
CaSSR86	(TC)9	AGCGATGAGAGCGAGTAAGC	TCTCTCCCCAATTCACCATC	59.9	278	Genic	Ca2	29913078	protein affected traf&#64257
CaSSR87	(CAG)7	TTGGTTAAGGATGTTCCTTCT	CTACCATCTGCTCTTGCTCTA	55.5	154	Genic	Ca2	30868340	CZF1
CaSSR88	(TA)11	AAAAACGCAAACACCaCTCC	GTGaAATAATTAGCTCTCTGATACCAT	60.0	237	Genomic	Ca2	31798047	
CaSSR89	(CAT)5	TGGAATGAaGAGATCcTCGC	CAAGTGGCAGCAGAAGTTCA	60.3	165	Genomic	Ca2	32013920	
CaSSR90	(GAA)11	CAAGTGAATGAATTCTCAAGC	CTCTCTTTCCTCTCATCTTCC	55.1	152	Genic	Ca2	32867739	EIN3 (ethylene-insensitive3)
CaSSR91	(GGT)4	CATGAACAACATCATCAACAA	CTCCACCACTACTTGTACCTG	55.3	148	Genic	Ca2	33605178	SHI gene family protein
CaSSR92	(TG)11	GGGGGTAAAgATTTCTCCACA	TCCCATAATTCAGGGATCTTG	60.2	225	Genomic	Ca2	33722683	
CaSSR93	(CTT)5	ACCATTTGGCATTGTTCCTC	GCGAAACCTGGAGTTCTTGT	59.3	266	Genic	Ca2	33745627	CLAVATA1-related receptor kinase-like protein
CaSSR94	(GAT)10	GCAACAAAAGAAAAGGCAGC	TTCTTCCTCCTCCTCtTCCC	60.0	195	Genomic	Ca2	34684799	
CaSSR95	(GAA)5	TTGAGCTGGAAGAAGGTCGT	CACTCCTCACATCCAACCCT	60.0	118	Genic	Ca2	35079352	trigger factor type chaperone family protein
CaSSR96	(CCA)6	ATTGATGACTCCGATTTCGC	ACCAGGTTGTCTACCCCAAA	59.3	148	Genic	Ca2	35422305	RNA-binding (RRM/RBD/RNP motifs) family protein
CaSSR97	(CT)6ca(CT)6	AAAATGCACTTGTGTTTTTGG	GCAACTTGATGCGAATTGAC	57.7	279	Genomic	Ca2	35431751	
CaSSR98	(TC)12	GATGGTGCTGTGAAATGTGC	TCCGCATTGAGATAAGGTCC	60.1	214	Genomic	Ca2	35754585	
CaSSR99	(TTCATT)4	AATGAGCTACTGCTGTCTTCA	CCAGCAAAGTAAAGAACTCAA	55.4	157	Genic	Ca3	80260	NAC domain transcription factor
CaSSR100	(TTTGAG)4	ATTCCACTTGTTGCTGATAAA	CTAAAAATGGAGGAGGAAGAG	55.0	151	Genic	Ca3	80320	NAC domain transcription factor
CaSSR101	(TTC)4	ACGCTTCAAGTATTGAACAAC	AAGGAAACCACACTTCACTCT	54.7	150	Genic	Ca3	81357	NAC domain transcription factor
CaSSR102	(AT)10	TGAAGCTAGGCTTGGTGGAT	ATTTTTCACCGACCGTTTTG	59.8	148	Genomic	Ca3	925403	
CaSSR103	(AAT)6	GGAGTTGGATTCTCTGGTGC	AATGGAACAAGTGGCTTTGC	59.7	240	Genomic	Ca3	2898679	
CaSSR104	(TC)14	CGGCCATTGAAAACGAATAC	TGGGTATTGGACTCCTTTCCT	60.3	266	Genomic	Ca3	3090422	
CaSSR105	(TAT)21	TGCTCTCTATTCGTTCCCTTTC	TCCGTTTAAGTTTCACTGCATTT	59.9	278	Genomic	Ca3	3745505	
CaSSR106	(TTA)31	TGTCATGCTTATTCCGACATCT	TCTcCCACCTCTGTTGCTTT	59.6	244	Genomic	Ca3	4772252	
CaSSR107	(TGA)5	TTGGAAGAAACACTGAATGAT	CTTTTTCTTCGTACGGTCATA	54.8	150	Genic	Ca3	7435046	SMAD/FHA domain-containing protein
CaSSR108	(AACAG)5	AGAGAAGAAGTCCAACAATCC	AGGTACAAAGAAAGTGCAACA	55.0	159	Genic	Ca3	10489944	ACT-domain containing protein
CaSSR109	(TTG)5	GGGTCATTTTGTTGCCTCAT	AATTTTGTGCATGACCCTGA	59.0	143	Genic	Ca3	12394016	Expressed protein
CaSSR110	(GA)9	TGAGGGAGAGAGACACATGAAa	CGGAAATTTTGTTGTCCAAG	59.8	212	Genomic	Ca3	15320676	
CaSSR111	(TTA)7	GAGGTTGGGGTTGGTTTCTT	CCATTCGCCTTCAATTTTGT	60.2	208	Genomic	Ca3	16451987	
CaSSR112	(ATTCAT)6	CCTTTCATTCATCACTTCAAC	AGGATAAAGGTGAAGCAAATC	54.8	165	Genic	Ca3	16813885	basic helix-loop-helix (bHLH) DNA-binding superfamily protein
CaSSR113	(TTTA)8	TGTGACATGATTTATGAATGACTTTTT	TTAGGGTTGTTCCAGTTGGC	60.1	207	Genomic	Ca3	17876577	

Marker IDs*	Microsatellite repeat-motifs	Forward Primer sequences (5'-3')	Reverse Primer sequences (5'-3')	Actual annealing temperature (0C)	Size (bp) of alleles amplified	Types of markers	Chromosomes	Physical positions (bp)	Putative functions
CaSSR114	(CATCAC)3	ACCATCATAACCATCATCATC	GGAAACCCTAGGTATTGTTGT	54.6	143	Genic	Ca3	20843695	one of two Arabidopsis RAPTOR/KOG1 homologs
CaSSR115	(CAT)6	CGTTTCCATTAGAGGAGAGAC	ATGAAAGTGATGGTGATAACG	55.6	162	Genic	Ca3	20843747	one of two Arabidopsis RAPTOR/KOG1 homologs
CaSSR116	(TCC)5	TCATCTGGGAAAAGGGAGTG	AAGGACCTCATCAAAACCAGAA	60.0	195	Genic	Ca3	21366194	WRKY Transcription Factor
CaSSR117	(AAC)8	ACACACTTCAAGAAACTTCCA	CTAAATCAGCAGCAGTAGGTG	54.9	150	Genic	Ca3	21774818	novel family similar to DNA binding proteins containing basic-leucine zipper regions
CaSSR118	(CTA)8	TCCCGTGAAACTGTCACAAA	CACCTGGAGAAGCTGAAATTG	59.9	277	Genic	Ca3	25631919	Expressed protein
CaSSR119	(CAA)7	TCTTACACCAAAACACAAACA	GAAGTGTTGTTGTTTCCCATA	54.3	154	Genic	Ca3	25672196	MYC-like bHLH transcriptional activator
CaSSR120	(AATGAA)4	AATAGTTCCAAAGGGTGAGAA	CCTTCTTGTTGGAATTGAACT	55.5	142	Genic	Ca3	26594189	GATA transcription factor gene GNC
CaSSR121	(ACA)7	TTTCAGTGTTTGTGGAAGACT	TGACTCATTTTGGTTTTGTCT	54.9	146	Genic	Ca3	26594514	GATA transcription factor gene GNC
CaSSR122	(TGT)4	TGAGTATCCCTTAATCACGAA	TGCACAGTAACAACAACTCAG	54.9	146	Genic	Ca3	27041765	BTB-POZ and MATH domain 4 (BPM4)
CaSSR123	(AT)7	CCAACCAATGAAAGCTAGGC	TCCTATACCAATCCCCCACA	60.0	134	Genic	Ca3	27694641	PHLOEM INTERCALATED WITH XYLEM (PXY)
CaSSR124	(CATCAC)3	AGATTACTCATTACCGCATCA	TCAAGCTATTGATCTTCTTGC	54.9	149	Genic	Ca3	28874001	HLH, Helix-loop-helix DNA-binding domain
CaSSR125#	(TAA)5	CGCATCTTCAATTCCATCCT	GGGCCCAACTCAAAAGTTTC	60.8	159	Genic	Ca3	29594640	ABC2_membrane, ABC-2 type transporter
CaSSR126	(CT)11	TGTCCCCATCTTCTTCATTCA	TTATTCAATGGGGTTCCAGC	59.8	241	Genic	Ca3	29681010	NIMA-related serine/threonine Putative protein kinases (Neks)
CaSSR127	(AT)16	TTCACAAACAACACCACACAa	TCACACGTGTCACAGTTCCA	58.5	174	Genomic	Ca3	30299506	
CaSSR128	(AG)6	TGAACCCAACAAGGAACCAT	CCCCATGTGCTAAAAGCAAC	60.5	223	Genic	Ca3	30690824	Plant protein of unknown function (DUF868)
CaSSR129	(TAT)6	AGTGGTGGAAGTATCCGTGG	AAGGATGAAAAACAGAGGGTG	58.2	237	Genic	Ca3	30756503	seed storage 2S albumin superfamily protein
CaSSR130	(GA)10	CCTCTGAAATGGGACTGTT	AACACTTCCCCACACAAAC	55.0	141	Genic	Ca3	31064037	ERF (ethylene response factor)
CaSSR131	(AAC)4	TTATCATCCAATGATGAATCC	ACTCCTCTAAACACCCTTTTG	54.8	155	Genic	Ca3	31248988	ERF (ethylene response factor)
CaSSR132	(TA)9	TCGTTTGCACTTGTTTTAGCC	TGTGAGCACTCATCGGTCAT	60.3	201	Genomic	Ca3	32341145	
CaSSR133	(TA)10	TCCTCCATTGCCTTTTCATC	GCATATTTTGGGACTAAGCATGT	60.0	229	Genomic	Ca3	32408558	
CaSSR135	(CTT)7	GCATCACAAGCTTCAACAGC	TTTTGGGTTGATGGGTGATT	60.0	213	Genic	Ca3	32584632	Chloroplast-targeted copper chaperone protein
CaSSR134	(CTT)9	GCATCACAAGCTTCAACAGC	TTTTGGGTTGATGGGTGATT	59.6	213	Genomic	Ca3	32584632	
CaSSR136	(AT)6(AG)7	AGGCTATAAGAATGTCcaCCTTT	AGAAATGCAAGCATCGTCAA	57.5	237	Genomic	Ca3	34902234	
CaSSR137	(CTT)5	ACATTCCCATTCCCATTGAA	ATTTGGAATCCATGGTGCTC	59.8	215	Genic	Ca3	35872876	FASCICLIN-like arabinogalactan protein 16 precursor (FLA16)
CaSSR138	(AG)19	ACCATTAACAGTCAACCATTG	CAGATATGTTCCTGCATTGAT	55.0	153	Genic	Ca3	36810456	group-S bZIP transcription factor
CaSSR139	(AAG)7	GATATCTGGATCGGGTCTATT	CATACCACACCATTTCAATCT	54.7	144	Genic	Ca3	36944041	Zinc knuckle (CCHC-type) family protein
CaSSR140	(TCT)5	TCCTAAATCTAATTCTATTTCGCATAA	ATCGCAAATGAAACGGAGTC	60.1	248	Genic	Ca3	37063772	RING/U-box superfamily protein
CaSSR141	(AATTGT)4	GGAACAACTCAAGCTCAGTAA	ATCCTTAGATCAGAGGAACCA	54.8	164	Genic	Ca3	37101948	squamosa promoter-binding protein-like 12 (SPL12)
CaSSR142	(AGA)6	CAGATTCCAAACGTGCAGTG	ATTGCAATGTGAACCCACAA	59.8	253	Genic	Ca3	37377922	Pectinacetylesterase family protein
CaSSR143	(GAC)4	TACATGTTCAGCACAGCTAGA	TCTTCTTCTTCGTCCTCTTCT	54.7	150	Genic	Ca3	37789311	ACT-domain containing protein
CaSSR144	(TA)9	AGGGAAACTTGACCCTCCAT	TGTTTGAAATTGCCACAAGC	59.8	191	Genomic	Ca3	38027665	
CaSSR145	(GAT)6	GAGCATCATAAGCGACGTGA	CCGCCAATCATATCCATTCT	59.7	140	Genic	Ca3	38354781	protein similar to glutathione synthetases
CaSSR146	(TAA)12	AATACGCATCCAATCCATCC	GTGTGGTGCTGCACAGAGTT	59.9	267	Genic	Ca3	38610591	Rubredoxin-like superfamily protein
CaSSR147	(GAA)7	CAAAGTAAACCCCACCGCTA	ACCGCAATATGGAACACGAT	60.2	259	Genic	Ca3	38624905	Expressed protein

Marker IDs*	Microsatellite repeat-motifs	Forward Primer sequences (5'-3')	Reverse Primer sequences (5'-3')	Actual annealing temperature (0C)	Size (bp) of alleles amplified	Types of markers	Chromosomes	Physical positions (bp)	Putative functions
CaSSR148	(TCA)8	AACACTTGAACATTTCAAACC	ATGGTGATGTTGTTACCAAAG	55.0	153	Genic	Ca3	38689024	Duplicated homeodomain-like superfamily protein
CaSSR149	(ATT)5	TCTCTCCCACAAAGGTCCC	GAAGGTGGCCAAGAGATGAA	60.2	134	Genic	Ca3	38689689	Duplicated homeodomain-like superfamily protein
CaSSR150	(ATC)7	ACCGTTCTCTAGGACGACCA	TGAGTGAAGATGATGACGGC	59.8	216	Genic	Ca3	39398787	WTF1
CaSSR151	(AG)7	GGTTTTGAGCGTGGTTTGAT	TGCAAAATGTGAAGCACAGA	59.0	146	Genic	Ca3	39618754	Expressed protein
CaSSR152	(AG)9(GT)6	TAGGTTTGTTGTGTGGGTCG	CCCTcACTCAGCGGTCTATC	59.5	142	Genomic	Ca3	39804933	
CaSSR153	(GAA)5	AGGAGGAGGTAGATATGATGG	CAAGTCTGTTCAGTTTCCCTA	54.8	156	Genic	Ca3	39817055	NGATHA3 (NGA3)
CaSSR154	(TATCAA)4	CCAAGGTCTAACTCACACTCA	TAGAAAGGAACTGTGATGAGC	55.4	134	Genic	Ca3	39817471	NGATHA3 (NGA3)
CaSSR155	(AAGA)6	ACTTCAGTGCTGTTTGATTTG	CCAACTAACAAACTTGGAAAC	55.6	155	Genic	Ca3	39818017	NGATHA3 (NGA3)
CaSSR156	(AT)14	TGGAGAGGAATTGTTAcGTGG	GCTTCCAATCAATAAAGTCTTACAA	60.0	222	Genomic	Ca3	39849058	
CaSSR157	(TCA)6	CGCAACTTCTCCCTTTCATC	TCATGGATTTCCTTTGCCTC	60.0	248	Genic	Ca3	39872382	endonuclease
CaSSR158	(TTTA)7	TTAGGCGCCACTCACTTTTT	TGTTCATTTGCATCGGAAAC	59.9	243	Genomic	Ca3	39872629	
CaSSR159	(CTT)17	CCGACCCAAcATTTTCATTT	TGCTGGAACGAACATTCAAC	59.7	174	Genomic	Ca4	820341	
CaSSR160	(TTGG)3	TTATGAAAATGATGGGTTGTC	AGACAACAGTGCCAAACTAAG	55.0	156	Genic	Ca4	1103152	NAC domain containing protein 74 (NAC074)
CaSSR161	(CAA)5	CGGTGATGAACCTGTTGTTG	AAGCCACTCAAGACGCTGTT	60.1	234	Genic	Ca4	1206702	12S seed storage protein
CaSSR162	(ATC)4	CAAGAAGTTTCTTCCACTTCA	CCAATCCATACTCTAACACCA	54.7	155	Genic	Ca4	1220485	ERF (ethylene response factor)
CaSSR163	(TA)8	TGTGCACATGACAATTAGAGCA	TTTCCATCAAGAACACGTCG	60.3	221	Genomic	Ca4	2543168	
CaSSR164	(TTA)5	AGATCCACCTCCACCTTGTG	TTGGAGGTTGTGTTGTTGGA	60.0	185	Genic	Ca4	2688512	AT-hook motif nuclear-localized protein 20 (AHL20)
CaSSR165	(AG)10	GTTAGGGATTTGTCGGAGCA	CAACGGTCACCTTTTTGGTT	60.1	217	Genomic	Ca4	3603795	
CaSSR166	(TA)8	CCACCTCTTCCCATCACTCA	TCATGGAGAATCCCAGATCC	59.8	271	Genic	Ca4	4594795	AT-hook motif nuclear-localized protein 19 (AHL19)
CaSSR167	(AG)6	GGGCAGTGAGAGATGAGAGG	CCTCACCCTTTTACCCACAA	59.8	148	Genic	Ca4	4699818	Nuclear protein that mediates light regulation of seedling development in a phytochrome-dependent manner
CaSSR168	(GATTCA)6	TGCAACTAGAGAAAGCTATGG	TGCGTTTTCTTTTAGTGCTTA	55.1	156	Genic	Ca4	4799223	ATRX
CaSSR169	(GATTCA)6	TGCAACTAGAGAAAGCTATGG	TGCGTTTTCTTTTAGTGCTTA	55.1	156	Genic	Ca4	4799223	ATRX
CaSSR170	(AAT)4	GAGAAAGAGGATATTGGGAGA	CTAACTCCAACATTCCTTGTG	55.0	151	Genic	Ca4	5080649	Duplicated homeodomain-like superfamily protein
CaSSR171	(ATA)10	CGGTTTGTTACTGAACTTGAA	CTTTGTCACCAATAAATCGTC	55.6	150	Genic	Ca4	5081312	Duplicated homeodomain-like superfamily protein
CaSSR172	(AAT)7	GTAGTGCAGATAAATGCAAGG	CAAGTTCAGTAACAAACCGATA	55.2	141	Genic	Ca4	5081434	Duplicated homeodomain-like superfamily protein
CaSSR173	(CAA)4	GACTATTCAATCCACTGGTCA	TTGAAATCAGTTACCTTCTCG	55.1	159	Genic	Ca4	5571877	UDP-Glycosyltransferase superfamily protein
CaSSR174	(ATC)4	AGATGATGATCACAAACATCC	TTGAAGTGAAGGAGATGAAGA	54.8	142	Genic	Ca4	5736891	indeterminate(ID)-domain 7 (IDD7)
CaSSR175	(AAC)4	TCATGACCTCATATCAAAACC	AGTTGCAGCAGATAAATGAAG	55.0	160	Genic	Ca4	5736936	indeterminate(ID)-domain 7 (IDD7)
CaSSR176	(ATG)8	TCAAAGGGAAAGGATTTTGG	AGGGTCCCTCAGTATTGCCT	60.0	145	Genic	Ca4	6590103	Major facilitator superfamily protein
CaSSR177	(CAA)7	CGGTGTGAATTTGGTGATGA	CAACAACAACCGAACGAGTG	60.2	253	Genic	Ca4	7315077	Tetratricopeptide repeat (TPR)-like superfamily protein
CaSSR178	(GAGAAA)4	TGAAACTTCAGAGGTTGGTAA	GCTTCTTGCTCTTGTTGTTC	55.0	144	Genic	Ca4	7561185	NAD(P)-binding Rossmann-fold superfamily protein
CaSSR179	(TGG)8	ATCCGGTAAATCAACATGAAT	CAGAACCCAGTTCTCATGTAA	55.9	157	Genic	Ca4	7970786	Zinc finger C-x8-C-x5-C-x3-H type family protein
CaSSR180	(ACCCT)5	TTCCTAAACGACATCTTCAAC	CAGTTTCTTCCACAAAAGCTA	54.5	153	Genic	Ca4	8051984	TLP family
CaSSR181	(ATG)7	GAAACTGAGCACTAGGAGTTG	CTTCTCTCTGCTTCATCTTCA	54.3	169	Genic	Ca4	8052130	TLP family
CaSSR182	(ATG)6	TAAACAAACCGGGAAGTTGG	CCCCTTACGAATCCCATTTT	60.0	137	Genic	Ca4	8052218	TLP family
CaSSR183	(GA)9	GAATCAGAAGGTGGTTGGGA	TTCGTGCCAGAAACAGTGAG	60.0	155	Genic	Ca4	9314839	protein with a DWD motif
CaSSR184	(TA)8	TGCATCTCGCCTTGAATATG	CCACACATGCCCCTACTCTT	60.0	214	Genic	Ca4	11051159	NAC domain containing protein 16 (NAC016)

Marker IDs*	Microsatellite repeat-motifs	Forward Primer sequences (5'-3')	Reverse Primer sequences (5'-3')	Actual annealing temperature (0C)	Size (bp) of alleles amplified	Types of markers	Chromosomes	Physical positions (bp)	Putative functions
CaSSR185	(GAT)4	AGGTTTTCAAGAAAAGTGGAC	GAGGATCAATAACAGCATTTG	55.1	147	Genic	Ca4	11053035	NAC domain containing protein 16 (NAC016)
CaSSR186	(ACC)5	ACTATTATGCACCCACTGAAG	GCAGAGACGAAGAAGATGATA	54.5	138	Genic	Ca4	11573408	Duplicated homeodomain-like superfamily protein
CaSSR187	(GAA)7	TGCTGGTCTTGTAGCTGGTG	GCAGCTTGTAAAGGGTTTGG	59.7	168	Genic	Ca4	11658503	Vacuolar iron transporter (VIT) family protein
CaSSR188	(CTCATC)4	CCACTAATACAAGGAAGCAAA	AGTCGCTCTTATTGGACTTGT	54.8	156	Genic	Ca4	12023919	Leucine-rich repeat protein kinase family protein
CaSSR189	(CTCATC)4	CATTCTTCAAATTCAAACCAC	TGGAAAAAGAAAGACAATGAC	54.8	136	Genic	Ca4	12023936	Leucine-rich repeat protein kinase family protein
CaSSR190	(AAC)7	CCCAAAACATCAACAATCTTA	CCTGTTGGAAATTTCTAGGTT	55.3	132	Genic	Ca4	12176090	SCARECROW-like 8 (SCL8)
CaSSR191	(TCA)4	CTAACTGAACCACCAGCACTA	AGAAGGAGAGGTGATAGGTTG	55.6	146	Genic	Ca4	13843207	LUH | WD-40 repeat family protein
CaSSR192	(AG)9	AAAATTGGATTGGGAAAGGG	TTTGCTACCAAACCAAACCC	59.8	131	Genic	Ca4	14340497	tubulin alpha-6 chain
CaSSR193	(AT)13	GGGATGCATCTGCAAATTTTA	TGAGTCCAATAAAACCCCCA	59.9	249	Genomic	Ca4	15398881	
CaSSR194	(AT)8	AACACCCCACATAATGACCC	TCATTTGACATTCACCTCACTTTT	59.9	222	Genic	Ca4	16478585	Expressed protein
CaSSR195	(ACTTGC)4	TGAACTCGTATCTTCTGCTTC	ATCATGCTTCACAACTTCATC	54.8	172	Genic	Ca4	16650296	ovate family protein 13 (OFP13)
CaSSR196	(ACTTGC)4	TGAACTCGTATCTTCTGCTTC	ATCATGCTTCACAACTTCATC	54.8	172	Genic	Ca4	16650296	ovate family protein 13 (OFP13)
CaSSR197	(TC)13	CGTCGTGCAGATCTTCGATA	GCGACGTCACTGTAAACCCT	60.0	248	Genomic	Ca4	16665218	
CaSSR198	(AT)14	CAATTTCCTCCTATTTGTTTTGACA	TGATTCTCGAGGATGCGAC	60.6	250	Genomic	Ca4	17043109	
CaSSR199	(CCA)6	CCAACTAGGCAAAAGCTTCG	GTTTGCAGGTTTGAGCTTCC	59.9	273	Genic	Ca4	17663778	RING-H2 protein that interacts with the RING finger domain of COP1
CaSSR200	(AT)9	AACTGAGAATGACTTGCCTTCA	TTTAGGGATGATTGCTTGGG	59.0	228	Genomic	Ca4	17704840	
CaSSR201	(ATT)8	TCTTTGAGACTGTTTTTCCCTTT	CATGTCTCATATTCCTCTAGCTGC	58.5	115	Genomic	Ca4	19856773	
CaSSR202	(ATTCT)4	GGGTCATCATCAAAACCA	AGTTCTGAAGTCATGGTAGGG	54.9	147	Genic	Ca4	20436604	TCP family transcription factor
CaSSR203	(TTG)9	CAGGAGTTGAAACTGAAACAC	TCCACAACAACAACAACAGTA	54.9	155	Genic	Ca4	22196748	embryo sac development arrest 31 (EDA31)
CaSSR204	(TA)10	AACCCATCATTTTGCCATGT	CCTGGAAAACTTGCAACTGG	60.1	155	Genomic	Ca4	22226606	
CaSSR205	(TA)10	GGGGATTGTCAGAAACGGTA	TTGTCGACACATCCTCTCAAA	59.8	223	Genomic	Ca4	26366347	
CaSSR206	(TCA)6	ACCACCACTGCCTCTTGTTC	CTCCAACAACTTCCCACGTT	60.0	149	Genic	Ca4	26873093	Expressed protein
CaSSR207	(AG)13	CACAATCATCATCGAGCACC	ACACACTCCTCACCGCTTCT	60.1	222	Genomic	Ca4	27187560	
CaSSR208	(AG)7	TCCACACAGACATGGTTCATC	CCTCATGGAAGGCAACCTTA	60.1	207	Genic	Ca4	28594218	AT-hook motif nuclear-localized protein 20 (AHL20)
CaSSR209	(CAG)6	TGGCATCATCATCACTGTCTT	CCATCCTTTTCAATGCGAGT	60.1	253	Genic	Ca4	30414975	cobalt ion binding
CaSSR210	(AT)9	TGTGAACTTTTCATTTGGCTTG	CCATTCGATCATGGGTAGTAGTT	60.1	205	Genomic	Ca4	30455676	
CaSSR211	(AAT)7	CCCCCACAAACTCAAAGTAAG	GCTGCATTTTCACCGTACAA	58.6	265	Genomic	Ca4	32335239	
CaSSR212	(ATGGT)3	TCCCATCAAGATGCTAAAATA	AATCAATCTTTGAGTTGTTGC	55.0	149	Genic	Ca4	32780320	SHY2/IAA3 regulates multiple auxin responses in roots
CaSSR213	(CAA)5	AAACAACTAACGCCGTAACTA	TTGGCATACGTATTCTTCTTC	54.5	153	Genic	Ca4	35416175	TCP family transcription factor
CaSSR214	(CCATT)4	TTCATTTAGCTTGAAGCACTC	GGTTGAGGAGAAAATTAAAGC	55.0	150	Genic	Ca4	35417258	TCP family transcription factor
CaSSR215	(TCC)5	AAACACAGATGTCGTCGCAA	CACTGATATTCGGCAACACG	60.1	156	Genic	Ca4	36155130	Plant protein of unknown function (DUF827)
CaSSR216	(CAC)6	CGTCGTTCACATACCAAACG	CCAGAAGGAGATCCTGAACG	59.8	207	Genic	Ca4	36842702	Putative plant-specific transcriptional regulator
CaSSR217	(CAC)8	CTAAAGAATGGAATTGGGATT	CTCGTTTGTTTGCTCTATTGT	55.0	153	Genic	Ca4	36842734	Putative plant-specific transcriptional regulator
CaSSR218	(GAT)4	TGAAGTTGAGAGGTTAATTGG	TCATCACACATAGAGCCTTTT	54.6	151	Genic	Ca4	36952282	indeterminate(ID)-domain 5 (IDD5)
CaSSR219	(TA)13	CTTCAACCCACCACGCTACT	CTCACTTCCTCCGTCTCTGC	60.2	271	Genomic	Ca4	38376225	
CaSSR220	(ATAA)7	TCTCCTTTCCCTCTAGTAATTTGTG	TTTGTTTTAAAATTGTCACTTCAACA	59.3	272	Genomic	Ca4	39006837	
CaSSR221	(TA)9	CATTCCACCTTAAAACTCCTCG	CCGTCTAACTAAGATGAACCCTG	60.0	199	Genomic	Ca4	39226042	
CaSSR222	(TA)6	ATAGCAGCAAATCCTCAGCG	GCTCATTGAAGATGACATTGC	58.3	273	Genic	Ca4	39805351	putative c-myb-like transcription factor
CaSSR223	(TC)9	TGTATGTAGGCTTGGGGAGG	ATCCAGATGATGCCCCTACA	59.9	170	Genomic	Ca4	40276977	
CaSSR224	(CT)7	CCTCAAGTGCAACAAAAACAA	TGCAAACATTTTCACACCAGA	60.1	124	Genic	Ca4	40670097	CCT motif family protein
CaSSR225	(CAG)5	TGCAACAGATGTTGTTACAGA	GAGATGAGCCCTATCCCTAC	55.3	170	Genic	Ca4	41865067	LEUNIG

Marker IDs*	Microsatellite repeat-motifs	Forward Primer sequences (5'-3')	Reverse Primer sequences (5'-3')	Actual annealing temperature (0C)	Size (bp) of alleles amplified	Types of markers	Chromosomes	Physical positions (bp)	Putative functions
CaSSR226	(CAG)4	AATTAAGGCTAGGGAACAACA	CATGCCTCTGTAACAACATCT	55.7	143	Genic	Ca4	41865183	LEUNIG
CaSSR227	(TA)12	ATGCCATTCTTGCCTGATTT	TCAAATGCAGCAACAACAAA	59.5	269	Genomic	Ca4	42025608	
CaSSR228	(ACA)4	GTTTGGATGCATAACAACAAG	CTCAGCATTCATATCAGCTTT	55.8	153	Genic	Ca4	43462315	homeobox protein 34 (HB34)
CaSSR229	(GAA)5	GAAAGAAGATCACAAGCCTTT	CAAAGGTATCTTGAGTGGTTG	55.3	155	Genic	Ca4	43513782	basic helix-loop helix transcription factor involved in tapetal cell development
CaSSR230	(GAA)8	TAGTTGCTGCCTTCGGAACT	GGCGGATACTACTTTGTCGC	59.7	200	Genic	Ca4	43715230	Ubiquitin-like superfamily protein
CaSSR231	(AATTAA)4	AATGAATTGGTTTGTTCTGTG	CTTACTCACCCGATCCTAACT	55.2	148	Genic	Ca4	43891008	A class II knotted1-like homeobox gene family
CaSSR232	(AGA)8	GTTTATGGTGTTGAAGGTTGA	TACTCTACGCTTCTTCCCTCT	55.2	149	Genic	Ca4	43891170	A class II knotted1-like homeobox gene family
CaSSR233	(AT)10	GTAAGCCAACTTCCTTGGCA	AACCCACAACCACTTCCATC	60.2	196	Genomic	Ca4	44581810	
CaSSR234	(ATG)5	AATGTTTCAATTTTCAATGGGA	GGATCGGTTATCGACTGAGG	59.5	189	Genic	Ca4	44625382	Expressed protein
CaSSR235	(AG)10	GGACACACAACAGAGAGAAAA	ACGAGGATTGTAAGGAGTACC	55.3	167	Genic	Ca4	44660343	AL4 the Alfin-Like family of nuclear-localized PhD domain
CaSSR236	(CAT)6	TCGCATAGAAGATGTCGTCG	ACGCTACTCGATGACGAGGT	59.9	152	Genic	Ca4	45074489	protein containing N-terminal tripartite nucleotide binding site
CaSSR237	(ATTT)9	GCTTCAGAATTCGACGTGGT	TACATTGGGCTGGTACATGG	60.3	105	Genomic	Ca4	45108920	
CaSSR238	(TGAA)3	ATGGAGGAAATTCTGAAACAT	AAGGAACTGTCTGTTGAAA	55.2	150	Genic	Ca4	45186457	transcription factor involved in photomorphogenesis
CaSSR239	(TAT)12	GGGTGTGGATAGCAATGGTT	AGCTCAATTGCCAGGAAGAA	60.0	274	Genic	Ca4	45445913	Putative homolog of the Blind gene in tomato
CaSSR240	(GTA)5	GGCGGAATAATTGAAGTAGTAG	GTTGTCGATCAAAAGTATTGC	55.0	158	Genic	Ca4	45595013	Growth regulating factor, transcription activatorr
CaSSR241	(GT)7	TTTTGTTAGCGTTTGTGTTTGG	CATAAAACCGCCTGCAACTT	60.1	203	Genic	Ca4	46259055	F-box protein
CaSSR242	(TC)10	CGTAACCGAGGAGTTTGGAA	AAGGGCGTTGAAAGAAAGAA	58.9	217	Genic	Ca4	46269177	Mannose-P-dolichol utilization defect 1 protein
CaSSR243	(AGATGA)3	CGATGAATTCAAGAAGCAATA	TGAACCTAACCAAATTCTTGA	55.7	159	Genic	Ca4	46398833	DREB subfamily A-5 of ERF/AP2 transcription factor family (RAP2.1)
CaSSR244	(AG)10	AATCAGAAGCGGAGTGGTTG	AATGGAGGCGGGAGAGTAAT	60.3	266	Genomic	Ca4	46457612	
CaSSR245	(TTA)21	ACAGAGCCACCAGGATTGTT	GAAACTGGCCAGACGtTTTT	59.6	225	Genomic	Ca4	46788418	
CaSSR246	(CT)7(AT)7	ATCTCGCCAATAACCACCAC	GGTAGCTAGTTGATCAAGTTCTTTCTT	59.8	197	Genomic	Ca4	47266711	
CaSSR247	(GAA)7	TGACATCTCCAACAACAAGAT	CACACTGTGGATTCTTGTCTC	55.6	147	Genic	Ca4	48124978	BEL1-like homeodomain 7 (BLH7)
CaSSR248	(ATA)6	GGCTATGCCAACATTCCTGT	TGAAGTAGGCCAATGCAACA	60.3	248	Genic	Ca4	48145725	cytosolic thioredoxin
CaSSR249	(TAAA)5	AAAGCATGCACTAATCACAGT	CAAGTCTTTGTCATCTTTGCT	55.1	139	Genic	Ca4	48319615	Basic-leucine zipper (bZIP) transcription factor family protein
CaSSR250	(TC)9(T)10	TTACATGAAAAATAAAAaCAAGCAA	CCGGAAGATTTAGCAATGGA	57.3	199	Genomic	Ca4	48825962	
CaSSR251	(GAT)6	ATGAGTCAAAGCCATAGTCAC	TTCTTCTTCAGTTGGATGAAA	54.4	153	Genic	Ca5	164592	LONG VEGETATIVE PHASE 1 (LOV1)
CaSSR252	(TCCC)3	CGAGTATACAAGAGAGCAGGA	AGCTGCCTTATTAGAGTGTCC	54.9	147	Genic	Ca5	166052	LONG VEGETATIVE PHASE 1 (LOV1)
CaSSR253	(CAA)5	TGACAACAGTTCTTGGTCTTT	GGGAGAGGGAGTAACAAAGTA	54.9	154	Genic	Ca5	166287	LONG VEGETATIVE PHASE 1 (LOV1)
CaSSR254#	(AATCCA)5	ATTCAGCAGTTAGTTCCAACA	GCATCAGCAAAGTAGACAAAT	55.1	147	Genic	Ca5	166458	LONG VEGETATIVE PHASE 1 (LOV1)
CaSSR255	(CTTTG)5	AATGGAGAAAAAGCAGAAGAC	GCGTAAACTAGATGGCTGATA	55.3	144	Genic	Ca5	222915	homeobox protein 31 (HB31)
CaSSR256	(TA)11	ATTGGGAATTCAGCGTTTCC	ACTGAGAATTGGGGTCgTTG	61.2	280	Genomic	Ca5	7247789	
CaSSR257	(ATTA)3	AACATGATGGAAGATGGAAC	TGGTCACTTGGGCTAATG	54.8	147	Genic	Ca5	7675104	a MADS box transcription factor expressed in the carpel and ovules
CaSSR258	(CT)15	TCTGCCTCGTACTCCTCACA	GGTTGTTGCTGGCATTTTCT	59.6	247	Genomic	Ca5	8866821	
CaSSR259	(TGT)4	TTGTTGATGTTGATGTTGCT	CCTATCAACCTATCGAACCTT	55.0	144	Genic	Ca5	9961600	WRKY transcription factor 2
CaSSR260	(TGC)5	ATTGATGATGAACCATTATGC	ACAACAACAACATCAACAACA	54.9	144	Genic	Ca5	9961692	WRKY transcription factor 2
CaSSR261	(ATA)6	TCGTCATAGTTTCAAGCGGT	CAGGAGGTGCACTATCAGCA	60.0	149	Genic	Ca5	9989246	chloride channel protein
CaSSR262	(TTC)9	TGCTATTGTGATTCAGTTCCT	AGCATGACAAGTTTAAAAGCA	55.0	147	Genic	Ca5	10080588	myb family transcription factor
CaSSR263	(AG)16	CAGAAAACAAACCAAAACCGA	TGCTCATGCATTTCAACCTC	60.0	191	Genomic	Ca5	10184550	

Marker IDs*	Microsatellite repeat-motifs	Forward Primer sequences (5'-3')	Reverse Primer sequences (5'-3')	Actual annealing temperature (0C)	Size (bp) of alleles amplified	Types of markers	Chromosomes	Physical positions (bp)	Putative functions
CaSSR264	(CTT)7	TCCAAAACACATTCACAAGCA	TATGGATCGGAAGACGAACC	60.1	245	Genomic	Ca5	11065299	
CaSSR265	(CTT)6	CCAAAACCATTTTCCTTCAATC	TATGGATCGGAAGACGAACC	59.9	191	Genic	Ca5	11065353	Plant invertase/pectin methylesterase inhibitor superfamily
CaSSR266	(AG)14	CGAATAAATTTGGCGGAGAA	AAGCATCCAAATTGGCAAAG	60.0	275	Genomic	Ca5	12476511	
CaSSR267	(GT)9(AT)6	AAGACATTATGAAATGCAACCAAA	TGAGAGATGCATTCAACATTATAGG	59.8	250	Genomic	Ca5	12592203	
CaSSR268	(AAT)10	TGATTTCTCCATCTtATCGGG	ATTAGCGCCTTACGACATCC	59.0	259	Genomic	Ca5	14185310	
CaSSR269	(ATT)8	AAAaGAACAAAATGGAAGCCC	TGGATGCAAAAGATTTGAGC	59.5	266	Genomic	Ca5	15878709	
CaSSR270	(AT)10	TCACGTCCATATTAATCATCAATTC	AGTGATAACCGTTGGAAAAGC	59.2	280	Genomic	Ca5	17120195	
CaSSR271	(CAC)4	CTTATGACATGAGGAAGCAAC	ATTTCTCTCACAATCAAATGG	55.0	150	Genic	Ca5	19048743	Acyl-CoA N-acyltransferases (NAT) superfamily protein
CaSSR272	(TCA)4	CAAGATTATCATTTGGACGAC	AAATGGTGCTAATTTGGATCT	54.7	150	Genic	Ca5	21386676	Pseudo response regulator involved in the generation of circadian rhythms, TOC1
CaSSR273#	(AT)10	CCTTTATAAAAaGAGGTGGGgT	GTGGGTTGCAAGGAAGATTG	57.7	201	Genomic	Ca5	23012241	
CaSSR274	(AT)12	TGCTTAAAGAATTTGAAACGATGA	CATTAGCCTCGTTGGCATTT	60.1	210	Genomic	Ca5	23438498	
CaSSR275	(AG)13	TACAAAAGAATCAAACCCAAG	TTTCTTCCACAATTCTCGTTA	54.6	165	Genic	Ca5	23874393	BTB/POZ domain-containing protein
CaSSR276	(AAT)7	TCCACTTCACCACATCATCA	TGCATATTTTGACCCTGCAT	59.0	209	Genic	Ca5	24717518	Homeodomain-like transcriptional regulator
CaSSR277	(AT)10	AACCGTTCACAGTAGAGGCA	CCCTATGTCCCAAAGCTACG	58.4	147	Genomic	Ca5	26329409	
CaSSR278	(GAT)7	TGAGGGTACTCCTAAACCCG	CCCCCTTCTTTTCCTTCAAC	59.0	180	Genomic	Ca5	26833175	
CaSSR279	(GAAA)3	AGCTATGCCTCCTGATAAACT	GAGTAGTGGCTTCTGTTTGAA	55.0	167	Genic	Ca5	27118508	basic leucine-zipper 52 (bZIP52)
CaSSR280	(CCT)4	AAGTAAACCACACGAGTTCCT	TATGAAGCTTTCAACATCAGG	55.5	144	Genic	Ca5	27757413	auxin response factor family protein
CaSSR281	(AAT)8	GCACATAGCAGTTACATATGGCAG	TACTTTTGCACCTCGGTTCC	60.9	265	Genomic	Ca5	28955959	
CaSSR282	(AT)7	CACAGCAACAGAACTGGCAT	TTCATGGCACAAGAAGTTGC	59.8	267	Genic	Ca5	29319100	protein, expressed in leaves, with similarity to pollen allergens.
CaSSR283	(CCTTCT)3	AGTTCTTCCACAAATTCAACC	CCAAGAACAGAACAAGAAGAA	55.8	152	Genic	Ca5	29398059	DREB subfamily A-6 of ERF/AP2 transcription factor family (RAP2.4)
CaSSR284	(CTT)5	TTCTCTTCCTCCATCAATGACTT	GCGAAAAGTTGGTTGGAAAA	60.1	155	Genic	Ca5	29465178	Contains a weak similarity to ELG protein from Homo sapiens
CaSSR285	(TCTTGC)3	TCTTTATCTTCTGCAAAACCA	CAAGAGCAAGAACAAGAAGAG	55.3	149	Genic	Ca5	30587088	nucleic acid binding
CaSSR286	(CTTCTC)4	TTTCATCGTCTACTTGATGGT	TTGATGGTAGAGCAAGAACAT	54.8	157	Genic	Ca5	30587245	nucleic acid binding
CaSSR287	(CTTCTC)4	TTTCATCGTCTACTTGATGGT	TTGATGGTAGAGCAAGAACAT	54.8	157	Genic	Ca5	30587245	nucleic acid binding
CaSSR288	(TTCTCC)3	TTTCATCGTCTACTTGATGGT	TTGATGGTAGAGCAAGAACAT	54.8	157	Genic	Ca5	30587245	nucleic acid binding
CaSSR289	(TAT)7	ACGCCTTCTTTGCTCCTTTT	AGTCAAGCCTCACTCCCAGA	60.4	250	Genomic	Ca5	31109261	
CaSSR290	(TTC)5	ATGAAATTGCTCCGTTGAGG	GGGATTTGATTCGTGGAAGA	59.9	258	Genic	Ca5	31182241	Calcium-binding EF hand family protein
CaSSR291	(ATG)6	CATGAAGGCCTATAACAAGAA	GAGCTTCTACTCGTCATCATC	54.7	145	Genic	Ca5	31194921	HMGB (high mobility group B) protein
CaSSR292	(ATT)9	AATTAGGGTTGGATGGAGGG	CTTCCGAACCACTACGCTTC	59.9	175	Genic	Ca5	31744505	RING/FYVE/PHD zinc finger superfamily protein
CaSSR293	(TC)6(TA)8	TCAATCTCCATCTTCCCCTG	CAACAAgGTCCCCACCAATA	60.0	222	Genomic	Ca5	31861702	
CaSSR294	(TG)7	TTTTTCCCTTTATCGCATGG	TAGGGGAAGGCAAATGTACG	60.0	145	Genic	Ca5	32856391	Expressed protein
CaSSR295	(CGG)5	CTGAGGATTTTGAAATTGAAC	TCATCTACACTCCGATCTCAC	54.0	145	Genic	Ca5	34050111	WRKY Transcription Factor
CaSSR296	(TAT)8	CCAGTTGTTGCCTCGGTATT	CAGGTTGATGTCCGAATGTG	60.0	273	Genic	Ca5	34521276	Expressed protein
CaSSR297	(AAG)10	CCAGCCATGGTAATTTGGAC	TTCTACAACCGTTTTTCTTCTCTG	60.2	204	Genomic	Ca5	34626059	
CaSSR298	(ATAA)9	TTtGGTTGCTAGACCAAGGG	CGGAATCCGATTGCTTCTAC	60.1	230	Genomic	Ca5	35432669	
CaSSR299	(AG)6	AAGCATCAGAAGAATCAGACAGG	CTTCCTCCTCGAGATCCTCC	60.3	220	Genic	Ca5	35796488	AGC (cAMP-dependent, cGMP-dependent and protein Putative protein kinase C)
CaSSR300	(CTTC)7	TCTAGAAGCTTCCTACTTCACC	CGAAAGTGAGTGTTGAGAAAG	54.7	163	Genic	Ca5	35861292	Heat Stress Transcription Factor (Hsf) family

Marker IDs*	Microsatellite repeat-motifs	Forward Primer sequences (5'-3')	Reverse Primer sequences (5'-3')	Actual annealing temperature (0C)	Size (bp) of alleles amplified	Types of markers	Chromosomes	Physical positions (bp)	Putative functions
CaSSR301	(TAT)5	CACTCCCTTCCTCCCAAAAT	CGCCTTTCGAATTATTGCAT	60.1	212	Genic	Ca5	36421460	Trypan_PARP, Procyclic acidic repetitive protein (PARP)
CaSSR302	(ATA)6	TCCATTTCCTTCCTTTTCCTC	GGTAGAAGGTGCTTGACCGA	60.3	219	Genic	Ca5	37739978	Chondroitin sulphate attachment domain
CaSSR303	(TTG)4	GGTATGACCAGAACATTTGAA	AACATCAATTCAATCATGGAG	55.1	142	Genic	Ca5	37896821	protein similar to a subunit of the CCAAT promoter motif binding complex
CaSSR304	(AT)10	AACAAGTCTGCAACAACCTTCT	CACAAAGTGAAGGGGGTCAT	58.0	270	Genomic	Ca5	38903229	
CaSSR305	(ACC)9	TTCTCTGAATCCAACGGTCC	TGTGTGCGTGAGAGAAGAGG	60.2	149	Genic	Ca5	40667653	Nodulin MtN21 /EamA-like transporter family protein
CaSSR306	(AAC)7	CTCTTGCAACTTCCCCACTC	AAAGCAAAGGAGGGTTTGGT	60.0	150	Genic	Ca5	41308534	Expressed protein
CaSSR307	(AG)6	ATGGTTTTGCTCACATTCTTA	TCTCAATTCTCTTCAATTCCA	55.0	151	Genic	Ca5	42499312	the MADs box transcription factor family
CaSSR308	(TC)11	CAGTGCCATCACTCCATCAC	CACGCaAAAACAAACCACAG	60.1	252	Genomic	Ca5	42534709	
CaSSR309	(GAA)4	GGAGGAAGAGGAAGAGAGAAT	GAGAGGACCTCAATTTCTTGT	55.7	172	Genic	Ca5	44153203	SWI3 gene family protein
CaSSR310	(ATG)4	TATAAACCACGCAAGTATTCG	GTACCCATCAAATCAACTTCA	55.6	146	Genic	Ca5	45306950	Homology Subgroup III
CaSSR311	(TA)6	CCGGCTTAATGAAAGAGGAA	GACCATGGGATGGATGTTTC	60.0	209	Genic	Ca5	45479771	Expressed protein
CaSSR312	(AG)8	AGAGTCAAATTAACAAAGAGCATAGA	AAAATGCCCACAAGAGCAAG	60.2	122	Genic	Ca5	46316346	CYP714A
CaSSR313	(CAC)9	AACCCATTTTGCAATCTGCT	CGCTGAGGAGAGAGTTCCAC	60.1	124	Genic	Ca5	46360697	RING/U-box superfamily protein
CaSSR314	(CCATA)4	AAAACAACACTCTCCTCTTCC	ACAGGTAGTGGGTATATTCCAG	55.1	143	Genic	Ca5	47007215	Transcription elongation factor (TFIIS) family protein
CaSSR315	(CAA)7	CAAGCTCACACTGAACTCTCT	GTTGTTGAGTTGTGGTGATTT	54.8	146	Genic	Ca6	466443	KANADI protein (KAN)
CaSSR316	(TCATTT)3	TCATAGATTCTTCTCCCTTCC	TGGAAACTTATAGATTTTGTGG	55.0	156	Genic	Ca6	1609131	DA1-related protein 2 (DAR2)
CaSSR317	(TTCTCT)3	CTCCACATCATTTTCATTTTC	AAACCAGACTTCCTTTCTGAG	54.7	173	Genic	Ca6	1609211	DA1-related protein 2 (DAR2)
CaSSR318	(TC)9	TAGGCATTTGCAACGCTATG	TTTTGCGTTCCTTTTCTCCT	59.9	202	Genomic	Ca6	2111716	
CaSSR319	(TTG)7	TCACCATCGTGTGATGGACT	TTGTTGGGTCCTCTTTTGTTTT	59.9	220	Genic	Ca6	2501203	Expressed protein
CaSSR320	(TTC)5	GCAGCAACATCTGGTGAGAA	CGTTCAGAGGTCTAGGGTGC	59.9	205	Genic	Ca6	2549860	RING/U-box superfamily protein
CaSSR321	(TTA)15	TCCCTTCCCCTCTTTTGTTT	TGTTTGGAAAACTGTGTTTAGAGT	59.9	203	Genomic	Ca6	2766175	
CaSSR322	(AAAGAG)5	GATGCACATCATCTTCTTCTG	GCAATATTGGAAATTCAGTGT	55.8	147	Genic	Ca6	3867508	NAC domain containing protein 75 (NAC075)
CaSSR323	(AT)12	CGTTTTTCATATTCAGGCCC	CGCATGCATACGGAACTCTA	59.4	187	Genomic	Ca6	4048430	
CaSSR324	(AG)7(A)13	CATAAATGGTTGCGACATGC	TCGACACAAACAAAATCACCA	60.0	168	Genomic	Ca6	4400440	
CaSSR325	(TTTTTC)4	ACAACTTACTGTCGTTGGAAA	ATTTCAAATCCTTCGTGAAAC	55.0	147	Genic	Ca6	4566075	Transcriptional co-activator
CaSSR326	(TTTTTC)4	ACAACTTACTGTCGTTGGAAA	ATTTCAAATCCTTCGTGAAAC	55.0	147	Genic	Ca6	4566075	Transcriptional co-activator
CaSSR327	(TA)8	CGGGTAATGAAACTCATAAATTAAA	CAATGCCTGCATTCCAATTA	57.8	274	Genomic	Ca6	5169574	
CaSSR328	(CAA)5	AAGACCAAGTCAAGGACTTTC	GAATACATATTCGTCGTCGTC	55.1	144	Genic	Ca6	5237484	Homeodomain protein
CaSSR329	(ATC)7	TGTTCCTTCTTCATCTTCAAA	TGATCTATTTTCTTGCCACAT	55.1	151	Genic	Ca6	5237688	Homeodomain protein
CaSSR330	(CAA)5	TGTTCCTTCTTCATCTTCAAA	TGATCTATTTTCTTGCCACAT	55.1	151	Genic	Ca6	5237688	Homeodomain protein
CaSSR331	(TCT)8	GGCAGCGACAACTACAACAA	TAATTGAGAATCGGGTTCGG	59.9	199	Genic	Ca6	5259895	BTB/POZ domain-containing protein
CaSSR332	(ATT)8	TCCTTGCCTTGTTCATAGTTA	GGCCCCTGTAGATGATGA	55.2	121	Genic	Ca6	6141841	Putative auxin response factor
CaSSR333	(GAA)8	TAATGAAAATTGTGGGAGAAG	TGACCCTCTTGTCATACTCAT	54.0	153	Genic	Ca6	6540055	Putative protein kinase similar to the calcium/calmodulin-dependent protein
CaSSR334	(TTC)12	ATCACTTCTTGCCATGTCCC	GGTGGTGGAATGAGGAGAAA	59.9	106	Genic	Ca6	6540055	TT viral orf 1
CaSSR335	(AAAT)12	CCGAATTCCCTGTTGTAGGA	ACTGCATGTTTGCCAGGTTT	59.9	276	Genomic	Ca6	6631430	
CaSSR336	(GA)9	TTCACTCAAATgTAGCAATCAAa	AATTATTGCCTGAGTTGCGG	57.5	214	Genomic	Ca6	7376505	
CaSSR337	(AT)8	TGCAGCAGAGCATCAAATCT	GTTGTCTGAAGGTCCCCAAA	59.9	135	Genic	Ca6	7531996	Expressed protein
CaSSR338	(AC)8	GCCACCGTTGTGTTCCTTAT	CGTTTTGAAAAATCGGTGGT	59.8	183	Genic	Ca6	7645002	LIP1 gene small GTPase
CaSSR339	(CT)20	TTTGGTGGTGAACGTTGAAA	GGAGAAGGaAAAGGAAGGGA	60.0	243	Genomic	Ca6	8335176	
CaSSR340	(AAAAC)5	CGTTGCTTAAGATACCAAAAC	AGAGCTTCCAAGTCTCTTCAT	54.4	179	Genic	Ca6	8368375	homeodomain leucine zipper class I (HD-Zip I) protein
CaSSR341	(TGA)9	TCATGGATTTCCTTTGCCTC	CGCAACTTCTCCCTTTCATC	60.0	248	Genomic	Ca6	8816079	
CaSSR342	(AG)10	AGGTAGTTGAAGAAACGAACC	AGAAGCAGGATCAATACCTTT	55.1	153	Genic	Ca6	9118284	Basic-leucine zipper (bZIP) transcription factor family protein

Marker IDs*	Microsatellite repeat-motifs	Forward Primer sequences (5'-3')	Reverse Primer sequences (5'-3')	Actual annealing temperature (0C)	Size (bp) of alleles amplified	Types of markers	Chromosomes	Physical positions (bp)	Putative functions
CaSSR343	(TGAT)4	TGATTGTGGATTCTTCGATAC	CAACTAACACCAAGAAGCAAC	55.2	150	Genic	Ca6	10053750	basic leucine-zipper 44 (bZIP44)
CaSSR344	(CAG)8	AATCTCAGCCTCAAACTCAAT	GCTGTTGTGATTTTTGTTGTT	55.6	147	Genic	Ca6	12437191	auxin response factor family protein
CaSSR345	(AC)12	TTCTTTTTGTTTCTCACCAAC	CCTTTGGTGTAAAAGGGTACT	54.6	138	Genic	Ca6	12569883	TEOSINTE BRANCHED 1, cycloidea and PCF transcription factor 2 (TCP2)
CaSSR346	(CCACTA)4	TTGCCATTATCATCTTCTTTC	ATAAATTGCACAGAAGGTGTG	54.6	141	Genic	Ca6	13095585	indeterminate(ID)-domain 14 (IDD14)
CaSSR347	(AACACC)3	AGCTCATATCAAAACATGTGG	GGTTGTAATGTTGTTGCTTGT	55.3	159	Genic	Ca6	13096321	Putative role in shoot gravitropism
CaSSR348	(AGA)6	ATCGCGTGAAGGAGAGAGAG	TACAACACCAACGACGCATT	60.0	141	Genic	Ca6	13949884	Protein of unknown function DUF1084 (InterPro:IPR009457)
CaSSR349	(AC)10	TGGCATCACAAGATTTCAATG	GCACTGTCATGAGAAAGCCA	59.5	251	Genomic	Ca6	14059898	
CaSSR350	(TC)11	CGTTTCTCGCTCTGGAGGTA	TTTCGTTGGTTACACGGTCA	60.0	253	Genic	Ca6	14301000	A-type cyclin-dependent Putative protein kinase
CaSSR351	(AAT)27	TGAAAGTGTTTTGAAAATTAAATGATG	GTGGCTACGGAAGTCTCCAA	59.7	255	Genomic	Ca6	14324174	
CaSSR352	(TTC)13	CTGCATCAACCACACCAATC	CAACGGATAATGCACTCCCT	60.0	136	Genic	Ca6	14759107	potassium transporter
CaSSR353	(ATA)11	TTGAGCACAAAGTTTCCTCT	TCCCTTCAATAGGTAAATTCC	55.2	151	Genic	Ca6	14811478	basic helix-loop-helix (bHLH) DNA-binding superfamily protein
CaSSR354	(AT)11	ATTGTTGGCAGGTTGTGGTT	CCGATTGTAGTGGGGCTAAT	60.3	230	Genomic	Ca6	15710297	
CaSSR355	(TCT)5	CATCAGTAACCGCTCAACGA	TTTGGAGGTGGAAAGAGTGG	60.1	244	Genic	Ca6	16936979	Ribosomal protein S5 family protein
CaSSR356	(TA)10	ATCAGCACCTCATACCCGAG	CGACAAGCCCAGACTCATAA	60.1	242	Genomic	Ca6	17580949	
CaSSR357	(TAT)9	TATCCCAAAAGTCACAATTCA	ATATGCAACACACAGAGGAAG	55.7	141	Genic	Ca6	18298837	ERF (ethylene response factor) subfamily B-3 of ERF/AP2 transcription factor family
CaSSR358	(AT)15	TTTTTCATCCATCACATCATCA	TTGATGCTTTACAACGTCGC	59.9	261	Genic	Ca6	19858207	F26K24.10 protein
CaSSR359	(ATT)15	CGGTTACCTATTTTTAATTGTGC	TTCTCCATCACACTGGGAGA	57.0	247	Genomic	Ca6	20442770	
CaSSR360	(TTGATA)3	GTGGAAGTGAATTCAAGTGAG	GTCCAACACCATAACAATCAT	54.8	148	Genic	Ca6	20514111	SET domain protein 35 (SDG35)
CaSSR361	(TTCACA)5	CCCACTTTCTCACTCACTCTT	TTCTGGAGGAGATAGTAGTGGT	56.1	163	Genic	Ca6	22249411	GRAS family transcription factor
CaSSR362	(CAA)8	ACCATTTGTTTGGGCATTTC	CGAATTGAGGGTTCTTCCAA	60.0	274	Genic	Ca6	23920468	NTMC2T5.2
CaSSR363	(AGA)4	GGATTAATTATCTCCGACCAG	TGGTAATCTTCCAGCTATCAA	55.4	122	Genic	Ca6	24448426	the R2R3 factor gene family
CaSSR364	(TC)6	ACCAACTCACATCTAGAAGCA	TGATCTTCCCATCTTATTTCA	55.0	157	Genic	Ca6	24448740	the R2R3 factor gene family
CaSSR365	(TAG)6	TGCAAACCACTTTTCCTTCTG	CGAATCACAAAATCCATCCC	60.1	140	Genic	Ca6	26357912	GRAS family Protein, transcription factor
CaSSR366	(TAG)7	AAGCCTTCTATATTGCAAACC	CAAAATCCATCCCTAGAACTT	55.4	146	Genic	Ca6	26357925	GRAS family Protein, transcription factor
CaSSR367	(ACT)7	TGGATAACCCTTTTCTTCTTC	AGCATTTGGTTTATGTAACGA	55.1	145	Genic	Ca6	26358317	GRAS family Protein, transcription factor
CaSSR368	(AGAA)5	GTGATCTGTCATCGTTTCAAT	AGGAAGCTTTGAGAAAGAGAA	55.0	141	Genic	Ca6	26358709	GRAS family Protein, transcription factor
CaSSR369	(AG)6	GCTATCGCCGATCAAGAGAA	TTCTAAGCTTTGGAAGAGATCCA	59.5	236	Genic	Ca6	26765102	Ras-related small GTP-binding family protein
CaSSR370	(ATT)8	AAAAaCCCGTGTCGTTTATTT	AGGGCAATATGCGATTCATT	57.6	256	Genomic	Ca6	26878517	
CaSSR371	(AT)14	CCATGCTTGTGACGTTCTCT	GCCTTTTCtTTTTCTTTGGGA	58.9	279	Genomic	Ca6	29119837	
CaSSR372	(GTG)4	GAGGAAGTTACCAAAAGCAAC	GGTTACATGTTTTGCATTCAT	55.7	162	Genic	Ca6	29256025	C2H2-type zinc finger family protein
CaSSR373	(TAT)32	TtCACTTGTTCAAGTACAACTATTtCA	GGAATGTGCCAACATCAGAA	58.5	273	Genomic	Ca6	30258394	
CaSSR374	(TA)10	GCGTTAGCGTCCTTGACTTC	TGAAGTGGTATATCTTAACCCAGGA	60.0	257	Genomic	Ca6	30465376	
CaSSR375	(AGA)7	GACCTCGAAATTGAGAAGCG	GCATCGTCATCTTTGCTTCA	60.0	109	Genic	Ca6	30580648	DNA-binding protein that binds to plastid DNA non-specifically
CaSSR376	(AAT)7	AGCGATTACCTTGAATGTGAGA	TCACACACATGGACCGAGTT	58.9	273	Genomic	Ca6	31079517	
CaSSR377	(TA)10	GGAGACAACTTCTCTTCACCAA	AGCCACTTCATTGACTTGTCTC	58.4	203	Genomic	Ca6	32082396	
CaSSR378#	(ATT)9	CATTCCCATATTTTCCTCCG	AAGACAATCGAATCCAACGG	59.9	266	Genic	Ca6	32200864	Expressed protein
CaSSR379	(GAA)6	TGTTCTGAATTCTGTTTCAGG	ACTTCCTCTGTCTATGCAGGT	55.4	150	Genic	Ca6	37387965	ovate family protein 4 (OFP4)
CaSSR380	(CTA)8	TGTTGCTCcTTTCTTTGCCT	GAGCATGTGGTGAAGCAGaA	60.0	152	Genomic	Ca6	37974670	

Marker IDs*	Microsatellite repeat-motifs	Forward Primer sequences (5'-3')	Reverse Primer sequences (5'-3')	Actual annealing temperature (0C)	Size (bp) of alleles amplified	Types of markers	Chromosomes	Physical positions (bp)	Putative functions
CaSSR381	(AG)12	TACCACTTTTATACGCTGCAC	ATGAATGAACGAATGTGACTC	55.7	145	Genic	Ca6	38814576	HAIRY MERISTEM 3 (HAM3)
CaSSR382	(AT)11	TTTTtGTTTTtGTCAATGTAATATGC	AAGCGAGAACTTACGGAACC	58.6	244	Genomic	Ca6	41910782	
CaSSR383	(GGA)7	TTTTGGAGGAGGAGATATAGG	AGTCATTTGTAATGGCATCC	55.1	163	Genic	Ca6	42715963	Dof-type zinc finger DNA-binding family protein
CaSSR384	(TA)11	CCCATAGAGAGCCCAACAAG	GGTCCtCTTTCTTCCGTCG	59.7	252	Genomic	Ca6	43988772	
CaSSR385	(AC)7(AT)6	CTTTCCATTTCCCATCACAA	AAGTCCTTGCAAACAAATCTCC	58.4	218	Genomic	Ca6	47487429	
CaSSR386	(TA)10	TGAAAATTAACGTACGGCACA	GATGACTTGTTCTCGTCGCA	59.1	153	Genomic	Ca6	49250840	
CaSSR387	(CT)15	CACATGTTCTTGCAACCCTG	ATTCaAAACCCaGAAAGCCC	60.2	154	Genomic	Ca6	50249027	
CaSSR388	(AG)16	AAACGCCTTGTCACCTTTTG	AATgGGGTGTTGAtTTTGGA	60.1	233	Genomic	Ca6	51773961	
CaSSR389	(AT)9	CCCtGCACTACACGAGGTCT	AAATACGTGTCTGCACGTCG	60.3	277	Genomic	Ca6	52161279	
CaSSR390	(TTA)7	CTCCTCAACCGCTGCATTAT	TGGTGGTTCTTCTTCCTACGTT	60.2	259	Genomic	Ca6	53565894	
CaSSR391	(CAA)7	TCAACTGTTCAACAACATCCT	GAGGAGGAGCACAAGAACTAT	55.6	163	Genic	Ca6	53652536	basic helix-loop-helix (bHLH) DNA-binding superfamily protein
CaSSR392	(AAT)6	CGGTAAGAGAAGAAGCCACG	TGCATTCAATTCAATTCCACA	59.9	280	Genic	Ca6	56766583	putative galacturonosyltransferase activity
CaSSR393	(GAA)6	GAATGGGAGGAAGTGGTTGA	CAACCCAACACCCCTCTCTA	60.0	198	Genic	Ca6	56796755	RING/U-box superfamily protein
CaSSR394	(AC)11	ACCGAAGAGTTTGATGGACG	GCTGCACTCATGGATGTGAC	60.1	137	Genomic	Ca6	56978935	
CaSSR395	(AT)7(AG)8	TCACCAACTCGTTGTTTACCTTT	TCAATAAACAAATTCAACATTCAAG	60.0	271	Genomic	Ca6	57785268	
CaSSR396	(AAG)6	TCGAGGATCTGATGAAGGAAG	TTCAGTTTCACGCAATCACC	59.7	236	Genic	Ca6	58296246	Major facilitator superfamily protein
CaSSR397	(TGTTCA)4	GAATCACAGCCATTAACCAT	GACAGTGTTGGAAAGACAAAG	55.0	147	Genic	Ca6	58334307	transcription factor CIB1 (cryptochrome-interacting basic-helix-loop-helix)
CaSSR398	(TA)6	TTGCTTTGAAGAAGGGGAAA	CCACTGCTGTCCTACACTGC	59.5	155	Genic	Ca7	1283953	Expressed protein
CaSSR399	(AGA)7	AACGATTCCAAAAGTTACGAT	GGGTTTCTGAATTAGGTTCAT	55.5	145	Genic	Ca7	1371794	WRKY Transcription Factor
CaSSR400	(CT)6(CA)7	TTTACACCCCCTAATTCCCC	CAATTTCGGCCAAGAACATT	59.9	151	Genomic	Ca7	1691706	
CaSSR401	(TC)8	CGATCAGTTAAAACCGCACA	TCTTCGACGAAACAAAGCAA	59.7	241	Genomic	Ca7	2219240	
CaSSR402	(TTA)7	GGGACAAGTCAGTCCGGTAA	ACACCACCCACCAGTAGGAA	60.3	196	Genic	Ca7	2222564	Expressed protein
CaSSR403	(AAT)7	ACACCACCCACCAGTAGGAA	GGGACAAGTCAGTCCGGTAA	60.0	196	Genic	Ca7	2222759	Syntaxin/t-SNARE family protein
CaSSR404	(TTC)7	AATGGAAGTGGAAGTAGTGGT	CTCCAATCCAATTTCCATAAT	55.3	139	Genic	Ca7	2334917	AP2 family of transcriptional regulators
CaSSR405	(TAT)5	CGCCACAACAACACCATATC	CGAAAGAAATTGATGCGAGA	59.0	202	Genic	Ca7	2944747	casein Putative protein kinase 1 protein family
CaSSR406	(TC)12	TCACTCCCTCGATCCTCAAC	AGAAACTTGGCAAAAGCAGC	59.6	280	Genic	Ca7	3046324	MAP Putative protein kinase Putative protein kinase 2
CaSSR407	(TGC)4	AGATTTTGTGATTCAGTCAGC	TCAACAGCAACAACAGTTACA	54.5	174	Genic	Ca7	3356103	Putative auxin response factor
CaSSR408	(AAG)10	CGGCCATTGAAATTGAAAA	GTTGGAAACAAACACGCCTT	60.0	276	Genic	Ca7	3393987	plastidic beta-ketoacyl-ACP synthase II
CaSSR409	(TCT)4	CAAATTGATGCTTATCAAACC	AGAAGGAGGAGAGATGCTTAG	54.9	159	Genic	Ca7	3750508	the Aux/IAA family of proteins
CaSSR410	(TA)8	AACGCTCCAACGTCTTAAAAT	ATGTATGAAACAAACAAACACGA	58.0	266	Genomic	Ca7	4219629	
CaSSR411	(CT)10	ATTCTCCTCATAAGCCATTCT	TTCTCGATCTATACGTCAACC	54.7	150	Genic	Ca7	6057942	A Class II KN1-like homeodomain transcription factors
CaSSR412	(ATTC)6	AGAATTCACAACCCACCGAC	ACATCACCTCCTCCGTTCAC	60.0	240	Genic	Ca7	7785589	serine/threonine protein Putative protein kinase.
CaSSR413	(TC)11	CCGATACCGGAGGATGTAGA	CCCAAACTCGACCTTGTTGT	60.0	239	Genic	Ca7	8489146	HNH endonuclease
CaSSR414	(CTT)7	ATCACAACGTGGTGGTAGCC	TCTTCTTCTCCTGGGGATCA	59.7	265	Genic	Ca7	8615603	TCP family transcription factor
CaSSR415	(CTT)9	TCGACATGATAACACAAGTCA	TAAGATCAAACAAACCTCACG	55.0	163	Genic	Ca7	8615627	TCP family transcription factor
CaSSR416	(TAT)7	CATCAACGACGACGAGAGAA	AACACTTGGCCGTTGGTTAC	60.0	214	Genomic	Ca7	8921807	
CaSSR417	(AGA)6	GAGGAGGTGAATTTGGCAGA	TGTTGGTTCTATTAACCCCCAT	59.5	217	Genic	Ca7	8979287	Dynein light chain type 1 family protein
CaSSR418	(AAGAAC)3	ATACTTCCCCTGAAGAAGATG	CCACGAACTCTCTTAACCTTT	55.0	142	Genic	Ca7	9867339	C2H2-like zinc finger protein
CaSSR419	(AT)11	CCACATCCACAAACACAACA	TGTTCACCCAAACCAACTCA	58.8	267	Genomic	Ca7	9969630	
CaSSR420	(AGA)12	GGAGCTGTGAACGGTGAAAT	CGAAACGCACCCTATTTTGT	60.1	207	Genomic	Ca7	10031195	

Marker IDs*	Microsatellite repeat-motifs	Forward Primer sequences (5'-3')	Reverse Primer sequences (5'-3')	Actual annealing temperature (0C)	Size (bp) of alleles amplified	Types of markers	Chromosomes	Physical positions (bp)	Putative functions
CaSSR421	(TGAATT)4	TGAACTATCCCCTTTAGGAAC	GAAGTTGATGAGTTTGATTCG	55.0	153	Genic	Ca7	10115566	ERF (ethylene response factor) subfamily B-3 of ERF/AP2 transcription factor family (ATERF-5)
CaSSR422	(TC)7	CCCAGAAAAGAGAAAACGCA	ATTTCTCAACTGTCTCGCCG	60.4	269	Genic	Ca7	10296770	Inositol phosphorylceramide synthase
CaSSR423	(GTG)4	AAGAGGTTCAAGAATTGAAGG	AGCCATAGAGAAAGTGGTTTT	55.2	149	Genic	Ca7	11192806	homeobox protein HAT22, the HD-Zip II family.
CaSSR424	(AC)8	CGTGGGACGAACTGAGTCTT	AGTTTGGCGCTGAATTTGAC	60.3	230	Genomic	Ca7	11425179	
CaSSR425	(AGA)6g(A)10	TTCTTCCTTAGAGCACAAACCC	TCCTCTCTCTCCGTTACCCA	59.8	190	Genomic	Ca7	11656778	
CaSSR426	(AT)7ag(T)11	TTCGTTTTTCATTTTTCTTCAGC	ACAACGTCGCACTAAAACGa	59.8	172	Genomic	Ca7	11960437	
CaSSR427	(ACACAA)4	CCTCTCTTATTTTGTTTGTCAAC	TGAAACTGTCGGTTCTGTAAC	54.9	165	Genic	Ca7	12021104	WRKY DNA-binding protein 15 (WRKY15).
CaSSR428	(TA)10	TTTGCAAATGGCTCAcTCAT	TTCAAAATGAAATATTCTTCTTCCG	59.3	233	Genomic	Ca7	14610251	
CaSSR429	(TAT)5	AAACTTGAGGGCAAGCCTTT	CAAAACACAGAGTTGAAGGAACA	59.3	103	Genic	Ca7	14738300	flavanone 3-hydroxylase
CaSSR430#	(AG)12	AAGCAGAGACCCATGTGGAG	AGTGGGGAGGGAAACAAACT	60.3	233	Genomic	Ca7	15721218	
CaSSR431	(ATGG)3	AGGTTTACCATCAGCTTCACT	TCCTTATGTTGCAATGTATCC	55.6	157	Genic	Ca7	17777145	G group bZIP transcription factor family member
CaSSR432	(ATGG)3	AGGTTTACCATCAGCTTCACT	TCCTTATGTTGCAATGTATCC	55.6	157	Genic	Ca7	17777145	G group bZIP transcription factor family member
CaSSR433	(TTC)7	GTCTTGGGAACCTTGCGTTA	GATGCGTAACTACAACCGCA	59.8	160	Genic	Ca7	18618008	Tetratricopeptide repeat (TPR)-like superfamily protein
CaSSR434	(ATT)28	TGAACAACTCAAATAAACAATCTTCC	TCTCACTAATCCaAAACaCCaAAA	59.8	257	Genomic	Ca7	19594142	
CaSSR435	(CT)18	ACCGCCTCAATTTCTTCTTA	TAATTTCACGGACAGTGTCTT	56.6	148	Genic	Ca7	19831349	PLATZ transcription factor family protein
CaSSR436	(TTC)7	AGTGATTCTCTGTGTCTCTGC	AGAATAGAAAACGGCAATGTT	54.5	155	Genic	Ca7	19942079	basic helix-loop-helix (bHLH) DNA-binding superfamily protein
CaSSR437	(TTC)7	AGTGATTCTCTGTGTCTCTGC	AGAATAGAAAACGGCAATGTT	54.5	155	Genic	Ca7	19942079	basic helix-loop-helix (bHLH) DNA-binding superfamily protein
CaSSR438	(GAA)8	AACATCATCATCAAGTTGGAG	TGAAGAAGAAGCACTTAATGG	55.1	154	Genic	Ca7	20120406	putative transcription factor; KANADI family
CaSSR439	(TAT)6	GGTTTGTTCCCTTTTACTTGT	CTCTTCAACTGAACCCTACCT	55.0	141	Genic	Ca7	20231155	Floral homeotic gene encoding a MADS domain protein homologous to SRF transcription factors
CaSSR440	(TA)11	TATCTCCACTCCCTCGTTCC	TGTTCTAGCAGGACCCTTCC	59.1	197	Genomic	Ca7	21614939	
CaSSR441	(AGC)4	CTGGTGTATTTTTCGTCAGTC	GATTCAAAATAGGGGTCTCAC	54.9	153	Genic	Ca7	21805504	Putative transposase
CaSSR442	(TTGGAA)3	ATGATGTTGGTGTTTCAAGAG	TAATATCACTGCGAAATCCAC	55.1	159	Genic	Ca7	24021097	homeobox protein 34 (HB34)
CaSSR443	(AT)13	CCGCAATCATATCAACTTtGTG	CCCTTCCAATTCTTCAATCC	60.4	243	Genomic	Ca7	24412796	
CaSSR444	(AT)6(AC)6	ATGCAAAGGTGTTTCGTGTG	CAAAGTGAGGATGAACCAATTTC	59.6	229	Genomic	Ca7	27443030	
CaSSR445#	(AG)14	AGCGGTAGGTACTTAAGAAGG	AAGCTGTCTCCTAAATGGAAG	54.7	155	Genic	Ca7	28547368	SBP-box gene, a the SPL gene family
CaSSR446	(AATT)6	GCTCCTCCATTAACTCATTTC	CAACTTCAAGCTCAATGTTTT	55.5	168	Genic	Ca7	28548004	SBP-box gene, a the SPL gene family
CaSSR447	(AG)6(A)12	TTGAATCGAAATGGGTGTGA	cCCCTTTGCCTTTATTCCAT	59.9	197	Genomic	Ca7	29567814	
CaSSR448	(AT)21	TCGGATTAGTGCATGaGAAAa	GGATAATACGAgTTTGTTCGCAA	58.4	205	Genomic	Ca7	30653052	
CaSSR449	(TC)12	CCCCTCcATCTACTGTTTCC	AAACCTAGCTTGGCAAAAACC	58.4	252	Genomic	Ca7	31192294	
CaSSR450	(TA)10	GTGACGGCCTCCTTAACAAC	TTTTGGTGGAACACTTGCCt	59.6	264	Genomic	Ca7	31642295	
CaSSR451	(TTGTGA)4	TTTGAAGGTCTTGTTGTTGTT	AGTGCAAGAGCTATGACAAAC	54.9	151	Genic	Ca7	31893675	indeterminate(ID)-domain 11 (IDD11)
CaSSR452	(TTGAGT)3	GACAAAGCTATAACCTCAGCA	CAGGAAACAGAACTGAAATTG	54.9	148	Genic	Ca7	31894863	indeterminate(ID)-domain 11 (IDD11)
CaSSR453	(TTTGAT)6	CAAGAAGGTGAAGTTAAAGCA	TCTTCAACAACAACAACAACA	55.0	155	Genic	Ca7	32855952	Growth regulating factor, transcription activator
CaSSR454	(TTTGAT)6	CAAGAAGGTGAAGTTAAAGCA	TCTTCAACAACAACAACAACA	55.0	155	Genic	Ca7	32855952	Growth regulating factor, transcription activator
CaSSR455	(TC)8	TCCAATGAGGTTGTGACGAA	CGGAGATGAATTTTCCGAGA	60.1	201	Genomic	Ca7	33218307	

Marker IDs*	Microsatellite repeat-motifs	Forward Primer sequences (5'-3')	Reverse Primer sequences (5'-3')	Actual annealing temperature (0C)	Size (bp) of alleles amplified	Types of markers	Chromosomes	Physical positions (bp)	Putative functions
CaSSR456	(AAT)9	CAAACGGAGAGGGATGAAGA	TGATCCATGCTTTTCCAAAT	58.0	270	Genic	Ca7	33782743	Putative 1-phosphatidylinositol-3-phosphate (PtdIns3P) 5-Putative protein kinase
CaSSR457	(ATG)4	CTTCTCCATCAACAACAAATC	CTTGTTTCCAAGTGACAATTC	54.8	161	Genic	Ca7	34320789	NGATHA3 (NGA3)
CaSSR458	(ATC)7	ACCAACCCTACAAGTCTCTTC	GTTATGCATCTCATGGTGAAC	55.0	142	Genic	Ca7	34923150	protein containing Dof zinc finger motifs
CaSSR459	(ATC)4	TCTTGAATCCAGCAGATTTAG	TGTATTTCTCTTTTCCTCGTG	54.8	150	Genic	Ca7	35180540	basic helix-loop-helix (bHLH) DNA-binding superfamily protein
CaSSR460	(TA)11	ACTAATGCCAATCGCTCAGG	ATGTGATGCGTGGTTGAGTC	60.2	267	Genomic	Ca7	35187940	
CaSSR461	(CAAGCC)3	ATGTCCATGCAAGTAAACAAG	CGACGACCTCTACCTTCTACT	55.3	137	Genic	Ca7	38472298	TCP family transcription factor
CaSSR462	(AAT)45	GTCATTTTCAACTGACTCATATTCAT	TTCTATGGaAAcCCAGTGAGC	57.8	247	Genomic	Ca7	42333636	
CaSSR463	(ATG)5	TACGGAGGCTATGGATGGTC	CACTCTTCCACTCCTTTGTGC	59.9	194	Genic	Ca7	48439099	Expressed protein
CaSSR464	(AAT)24	TCACCTTCATTGTTGGTCGT	TTGTAAATTGTGGCACCCAG	59.0	256	Genomic	Ca7	48752339	
CaSSR465	(TTC)5	CCCTCTTCTCTACGACACCG	CGGTGGaCATCCTCGTATCT	59.9	279	Genomic	Ca8	1930653	
CaSSR466	(AG)6	TGTCAAAAGCGAGAATTCCA	GGAAGCAGCTGGTTTTGTTG	60.8	280	Genic	Ca8	2001637	protein with hydroxymethylglutaryl-CoA synthase activity
CaSSR467	(ATA)7	CAAAGGGGAAGAAGTGTTGC	GCCATTTCTGGAGTTGGAAA	59.7	219	Genomic	Ca8	2524466	
CaSSR468	(TGAT)3	GCTCTAAGACCCAATTGTTTT	GAATCAGTGATGAAGAAGACG	55.2	151	Genic	Ca8	2557055	homeodomain-leucine zipper protein
CaSSR469	(CAA)7	TTTCAACAATGCCAACCAAA	TCGAAGAAGGGGAAGAAACA	59.8	211	Genic	Ca8	3154215	basic helix-loop-helix (bHLH) DNA-binding superfamily protein
CaSSR470	(AT)6	TTTTCAAAGAACCCCAACAGA	AGCAGGACATTTGTGCAGTG	59.9	159	Genic	Ca8	3763063	Chaperone DnaJ-domain superfamily protein
CaSSR471	(ACT)7	CAAAACCCCATAATGCCACT	TTAGCTGAGCTGTTTGGCG	60.3	205	Genic	Ca8	3945750	Phototropic-responsive NPH3 family protein
CaSSR472	(TGA)5	TCCAATTCCTCACAACCACA	CTCTTAACTCCGCCGTCAAC	59.9	197	Genic	Ca8	4243742	Homeodomain-like superfamily protein
CaSSR473	(TTA)9	AAGTTGAGGAAGGGGATGGT	TAGTGGGCTTCGTATTTGGG	59.8	274	Genomic	Ca8	4270429	
CaSSR474	(TGT)7	ATATTTTTGTTGTTGGTGTCG	GAAGCCAATGGTAGAAGAAAT	55.2	155	Genic	Ca8	4623460	BEL family of homeodomain proteins
CaSSR475	(TGT)7	ATATTTTTGTTGTTGGTGTCG	GAAGCCAATGGTAGAAGAAAT	55.2	155	Genic	Ca8	4623460	BEL family of homeodomain proteins
CaSSR476	(AG)6aaagaga(GT)[illegible]	CGCGAAGAGAAAGAGACGAG	TGCAGGGTATGAAAGGATGA	60.4	141	Genomic	Ca8	4628934	
CaSSR477	(TTC)4	TAGATCTCACACACACAATGG	GTTATGGTGTGAATTTTGAGG	54.4	143	Genic	Ca8	5196644	homeobox protein 24 (HB24)
CaSSR478	(ATT)4	TCTACAGACACAAAAGGGGTA	CACTTATCTGTCGCACTCTTT	54.9	150	Genic	Ca8	5411626	SHI gene family protein
CaSSR479	(AG)7	CACAAAAGGGGTAAGTGGTCA	CGCATTCAACTCATTCGTGT	59.7	232	Genic	Ca8	5411634	SHI gene family protein
CaSSR480#	(TTGAG)4	AATCCAATCGAAGAGAGAGAG	ACTCTAGGGTTTTCACTTTCC	55.3	154	Genic	Ca8	5569895	DNA binding
CaSSR481	(GAA)6	TGCAAGTTAACACGAGCACC	TTCCAGCAAATTTTGGAACC	59.9	189	Genic	Ca8	5822343	Chalcone-flavanone isomerase family protein
CaSSR482	(GGT)5	TCCTCTTCGACAATTCGCTT	GAAGGAATTGGTGCTTTTGG	59.5	173	Genic	Ca8	6160737	Expressed protein
CaSSR483	(TCT)8	TTCAATCTCTTCACGCCCTT	GAAGGTTTCGAGTGCCTCAG	60.0	160	Genic	Ca8	6688803	Pentatricopeptide repeat (PPR) superfamily protein
CaSSR484	(AAG)8	GAACCAAACATGCACAATGC	TCCTGCGTATCTGTGTCTTTGT	60.0	278	Genomic	Ca8	7731588	
CaSSR485	(CTA)7	CACAAGGGTTTATGATAGTGC	CCATGATAAGAAGACAACCTG	54.9	150	Genic	Ca8	8364902	homeobox-7 (HB-7)
CaSSR486	(AGA)4	TGAGATTGGTGTAAAGAAAGG	AATTTGTCCACCTTAGCCTAC	54.6	155	Genic	Ca8	8639326	the R2R3 factor gene family.
CaSSR487	(AATAAC)5	TCACAGAAGCAGAATCAAAGT	CAAAACTCAGAATCTCACCAG	55.2	148	Genic	Ca8	8841425	MYC-related transcriptional activator
CaSSR488	(AT)9	AGGCCAGTTGACACCATAGC	GCTGGAACCATTTCCAATGT	60.1	171	Genomic	Ca8	10925248	
CaSSR489	(TA)10	TTgaATATTGTGTTTGATTTCCTTT	CCCTCATATTCAAAACAATCCC	58.1	219	Genomic	Ca8	11159303	
CaSSR490	(AT)10	CAAATCAATATACAACACATCACTCC	CTTGCTCCTACGTATCCCCA	58.8	215	Genomic	Ca8	12030526	
CaSSR491	(TA)10	TGTCTTCAAAAGGATAACTCATGC	TCATTTTGAGTGTTGAAGATTATgG	59.7	207	Genomic	Ca8	13144169	
CaSSR492	(ACA)5	CCCAGATACAATGCATACGCT	CCGATTTTCCTTCCTCAACA	60.0	222	Genic	Ca8	14017713	Expressed protein
CaSSR493	(CCT)5	CATCATCTGTTGCCTCTTAGT	ATGAAGATGATGATTTCGATG	54.5	167	Genic	Ca8	14955395	Alcohol dehydrogenase transcription factor Myb/SANT-like family protein

Marker IDs*	Microsatellite repeat-motifs	Forward Primer sequences (5'-3')	Reverse Primer sequences (5'-3')	Actual annealing temperature (0C)	Size (bp) of alleles amplified	Types of markers	Chromosomes	Physical positions (bp)	Putative functions
CaSSR494	(ATCAAA)4	AAGTACTGTGAATGCTTCCAA	CTTGCTGAATATACGTGTGGT	55.1	156	Genic	Ca8	15220963	Tesmin/TSO1-like CXC domain-containing protein
CaSSR495	(ATCAAA)4	AAGTACTGTGAATGCTTCCAA	CTTGCTGAATATACGTGTGGT	55.1	156	Genic	Ca8	15220963	Tesmin/TSO1-like CXC domain-containing protein
CaSSR496	(TCT)4	TAAATCGCCTAGAGATGTTCA	CATAACTTGTTCCGAGTCTTG	55.2	155	Genic	Ca8	15607448	DREB subfamily A-4 of ERF/AP2 transcription factor family

*CaSSR *Cicer arietinum* SSR

Supplementary Table S4: Summary of 384 TF gene-derived SNPs used for polymorphism survey between parental accessions (ICC 4958 and ICC 17160) of RIL mapping population employing high-throughput MALDI-TOF mass array

SNP IDs*	1st Pre-PCR primer sequence	2nd Pre-PCR primer sequence	Unextended Primer sequence	SNP allele (ICC 4958)	SNP allele (ICC 17160)	Size (bp) of alleles amplified	Actual annealing temperature (0C)	Chromosomes	Physical positions (bp)	Transcription factors (TFs)
CaTFSNP1	acgttggatgTCCCAACAAGGAAGCTAGTG	acgttggatgTCAGGTTCCTTCTTAGTTGC	AGCGTTCTTAGCAGGACCTTCATTA	C	T	98	49.1	CaChr1	1661476	GeBP
CaTFSNP2	acgttggatgTCAACAAGACAGTTCGAAGCA	acgttggatgCATCAGCGTGTCTTTTGAGG	GTTCCGAAGACAGGATGTAC	T	C	108	59.62	CaChr1	1827593	Jumonji
CaTFSNP3	acgttggatgCACTGGAGGTGTTGCTTTCA	acgttggatgTTCTCCCCAACAGAACCAAC	CCCCAACAGAACCAACTGAA	G	A	199	59.87	CaChr1	2188544	SNF2
CaTFSNP4	acgttggatgTGATCCTCCAGCTTTTGACC	acgttggatgAAAGGTGGGGTGAGGAAAAG	AAAACCTCTTCTTCATCTTCATCATC	A	T	158	60.2	CaChr1	2602506	SBP
CaTFSNP5	acgttggatgGAGGAGGAAGTAGAAACAATAGA	acgttggatgTCCTCCTCCTCCTCTTCT	AGAGGAGGAAGTAGAAACAATAGA	G	A	101	53.77	CaChr1	2631901	C3H
CaTFSNP6	acgttggatgTGACTGCCAAGGAACTCAAA	acgttggatgTCCCTCTTCTGCATGTATTCAA	GGATTCGTTCCAAAAAGAAC	G	C	115	59.41	CaChr1	3650162	MADS
CaTFSNP7	acgttggatgAAAGGGAAGCATCAGGGAAT	acgttggatgTGCTAAAATCCATGGGATGA	GCCAAATGAATAAAAGGCAGAA	G	A	201	59.9	CaChr1	3734533	Bromodomain
CaTFSNP8	acgttggatgGCCTTGCTCTGCCCTAATCT	acgttggatgTTGTGAAAGAGGGGTTTTGG	TGCTCTGCCCTAATCTCAAC	G	C	109	60.87	CaChr1	4325063	mTERF
CaTFSNP9	acgttggatgCCCCCTCTCAATGTTTTTGA	acgttggatgTTTCATCATGGAGCCGAAA	CATAATCATTTAGTTCCCTGTATCTTTCT	T	C	197	59.9	CaChr1	4379384	SNF2
CaTFSNP10	acgttggatgGGGTGTTGTTGCTCCTGAAT	acgttggatgTCTTTGGACGATTCACTGCT	CATCTAATGTTGCTGTAACTGTGAGT	G	T	228	59.97	CaChr1	4866342	BSD
CaTFSNP11	acgttggatgGCAACTTCTTATTTAATTAT	acgttggatgCCATTTAGTAGATTTTCATC	CATATAATATTTATCCTAAAAATGCAAG	C	G	109	46	CaChr1	5452945	MYB-related
CaTFSNP12#	acgttggatgCAAGGAACCAAAGAAGAAAAGG	acgttggatgGGTGCTCATGGAAACAACAA	GAAAAACTACTAATTGTATTTCTGCTG	T	C	103	59.28	CaChr1	5666051	WRKY
CaTFSNP13	acgttggatgCCCGTGAAAACTGGGATTTA	acgttggatgAGTCACCCGAACAAATCGTC	CCTGGGCATACTCACCTGTA	G	A	206	59.79	CaChr1	6592726	FHA
CaTFSNP14	acgttggatgGGTGCCAAAGTTGCTTCAAT	acgttggatgGGATCAGTTCAAAGGGTTGC	TTCAGAACTGAGTCCTCCGC	A	T	199	60.12	CaChr1	7417345	HB
CaTFSNP15	acgttggatgATCCAATGGCCAATCTTCAG	acgttggatgAAATGTCGAGACTCGTGCAA	TGCAGGTGTTCCTTTGCC	G	A	199	59.89	CaChr1	7436472	CCAAT
CaTFSNP16	acgttggatgTGTCATGCAACCTAAACACCA	acgttggatgGTTACTTCCAACCGGCACAT	TTTGGTACACAAGTTTCACCTATCTC	G	C	206	60.02	CaChr1	8284578	MYB-related
CaTFSNP17	acgttggatgCTCCGAATACAGAAGCCATC	acgttggatgAGTACAGCCCTTTACTTCGC	CGAGACCCTTCTAAACC	G	A	100	46.3	CaChr1	9654075	Trihelix
CaTFSNP18	acgttggatgGAGTCTGCAACCGCAATTTT	acgttggatgTGGCAATGATGACATGATGA	GATGATCTTGTTGACAAATACC	G	C	117	60.26	CaChr1	10455472	MYB
CaTFSNP19	acgttggatgACCTTGTCGCTCGTGATTCT	acgttggatgGATGGAGGGTGGTGGTCTTA	TGAATTCAAATTCGAATAACTCC	G	A	115	59.87	CaChr1	11285630	GRAS
CaTFSNP20	acgttggatgGGGAGCTTGTGAGGATTTGA	acgttggatgGTACTGCTTCCCCGGTACAA	GGATGGAGCGAAACAGAGTGT	G	A	197	60.2	CaChr1	12530205	AP2-EREBP
CaTFSNP21	acgttggatgCACTGCAAGTAGCACACTCACA	acgttggatgGGAAGCTGGCAAGAAGAAGA	ACAAAAAACATAACATTCACATAA	G	C	110	60.16	CaChr1	12928919	G2-like
CaTFSNP22	acgttggatgATTTCCCAATGCAGTCGGTG	acgttggatgAAAGTTCAGGCGCGCACAAG	CCATACATTGCCCTTGC	C	T	102	48.6	CaChr1	13679988	AP2-EREBP
CaTFSNP23	acgttggatgTCTGCTGGTTCATCCTGTTG	acgttggatgTGTTCGTGTGAGGAATCCAA	GCTCTATGATTATGGATTTGA	T	C	118	59.83	CaChr1	13801427	bHLH
CaTFSNP24	acgttggatgTTGTCTGGTGAAGTTGCTTGA	acgttggatgCCCGAACCTTAATCCTCTCA	CCTAAGTTCCCTACACCTCCTC	T	C	203	59.47	CaChr1	13994815	HB
CaTFSNP25	acgttggatgCATTTCGGAGAGAAGGAGGA	acgttggatgTGCTTCAAGTCCTGAAACCTC	CCAACATTACTAAGATGGATAAGGC	A	C	178	59.36	CaChr1	15378650	bHLH
CaTFSNP26	acgttggatgACTGTCCATCCCGAGGATCT	acgttggatgCGATTCATCTTCCTCCCTCA	GCGTTCTTGTAAGCCCGAT	A	C	201	60.88	CaChr1	15606770	WRKY
CaTFSNP27	acgttggatgGCACCCGATGATAACGAAGT	acgttggatgTCCTCCAACTTTCGTCGTTT	GGGCGAGAGTGTCAGAGAGAC	G	T	200	59.96	CaChr1	16080907	GRAS
CaTFSNP28	acgttggatgAGCTTTCCAATACCCAGATCC	acgttggatgCGCAAAGTAGCTGGTCATCA	TTCAGTCCAAGAGATAGGAAGTATCC	T	C	192	59.43	CaChr1	16680465	NAC
CaTFSNP29	acgttggatgGATGAGGCTTGCTCATTTGC	acgttggatgCTCTTTCAATGCGGTTGGTG	AATGCGGTTGGTGATATTTTACTAGC	T	C	105	54.2	CaChr1	17405553	CAMTA
CaTFSNP30	acgttggatgGGGGGCTAGTCTGGAGAGAA	acgttggatgTGGCATGTGCAAGATTGAGT	AAACATAGTATCAACGACTACAAATGGA	A	C	209	60.73	CaChr1	18460430	mTERF
CaTFSNP31	acgttggatgAGGAGGAAAAACCAGGCTGT	acgttggatgGGCACTCTTTCTCTCCCAGA	GGCCAGACAGAATGCAAGTATTA	T	C	211	60.11	CaChr1	20598392	C3H
CaTFSNP32	acgttggatgACCTTTCCTGTTCTCTGAGC	acgttggatgAAATCGAACGTAGCCTAAGC	CGGGCAAGAAAGAATCAATTGTTCA	C	A	107	52.8	CaChr1	21135977	MADS
CaTFSNP33	acgttggatgTGCAGCATCCTGAATCTGAG	acgttggatgAAATGGGTGTCGGAAATCAG	CGGGAAATTAAACTTAGCACCAC	T	C	204	60.1	CaChr1	23850218	AP2-EREBP
CaTFSNP34	acgttggatgTTTGCAAAAGATGCAGTTCA	acgttggatgAAGCCTAATCAAGCACTATCAGC	CCATTAAAAAAGCAGTGTGCTGA	G	A	188	58.05	CaChr1	24068638	ARF
CaTFSNP35	acgttggatgTGGCTAAAACTGTTGCCTTC	acgttggatgGAACCCCACAAATGGATCAC	GGGGAAAATGGATCACAAGAAGTC	T	C	86	45.3	CaChr1	25186105	Tify
CaTFSNP36	acgttggatgCCGTTTGAATGCATATTGGA	acgttggatgGAGGAGGAAGACACATCCTGA	GACACATCCTGATGCTGCT	A	C	120	59.38	CaChr1	26026317	SBP
CaTFSNP37	acgttggatgCTTCCAGATCTTCCAGAGTG	acgttggatgTAAGCGTAGCTGTCGCAAAC	TAGCGCCGAAGAAAGC	C	T	107	50.1	CaChr1	33623744	SBP
CaTFSNP38	acgttggatgAGAGGTGGTGAACCAAATTC	acgttggatgTGTTGGACCTCAAGGAATGC	CCGCCTGAAAATATGGCACT	G	T	83	48	CaChr1	37691837	G2-like
CaTFSNP39	acgttggatgCAATTTTTTGCTTATGGGTC	acgttggatgCTTGACCTTAACGTTCCTCC	CCTGAAGTTGCTTGATCTG	G	T	95	48.6	CaChr1	41981594	AP2-EREBP
CaTFSNP40	acgttggatgCAAACACATTTGGAGGAGCA	acgttggatgTGGGTTATTTCTTTCCATTTTTG	CCCTTTATCATGTGATAGAAAAGT	G	C	119	59.69	CaChr1	43957759	NAC
CaTFSNP41	acgttggatgGGCTTTTCCTCCTATTCTGC	acgttggatgCCATGAATCTGGTTCAAGGC	GAGGGCTCTAGTTTGAA	C	G	95	45.5	CaChr1	46718906	mTERF
CaTFSNP42	acgttggatgGAAACTTGCTTATTTTGCACCA	acgttggatgGAACCAATTGCATTTCGTACAG	TGTTTCAATACATAGCAACTAGATAT	T	C	98	59.3	CaChr1	48002347	PHD
CaTFSNP43	acgttggatgCGCAGCAGCTACCACATAAC	acgttggatgGAAGCGAAGAAATTGGAGCA	GACGAAAGAAATGTGGAGAAATC	T	C	184	59.52	CaChr1	48218880	Alfin-like
CaTFSNP44	acgttggatgATTCCTCGATTTCTCCTCGG	acgttggatgAATGATGTCTCGAGGAAGGC	GGGCGAGGAAGGCAATAGAGTCACT	C	A	101	54.3	CaChr1	48249846	bZIP
CaTFSNP45	acgttggatgTCTCTTCTTTCGACACGTGG	acgttggatgGCCAATAACTTCGGAAGCAG	GAGTCGGAAGCAGCTGCTAA	G	A	103	51.5	CaChr2	355965	LOB
CaTFSNP46	acgttggatgCCAAGGCCAAAAATTCTGTT	acgttggatgTCATGGAGCAATGGAAGTGA	GCATTGAAGTAGTTGTGTCATGCT	A	T	231	59.06	CaChr2	3202163	HB
CaTFSNP47	acgttggatgATTGACCCTTTTGCCCTTCC	acgttggatgCAAACCAACATTGACATGGC	CCATTGACATGGCAAAAAAAGCACTTAG	G	A	112	54.5	CaChr2	3353616	MYB
CaTFSNP48	acgttggatgCATGCAGCTAATGTTGGAGGT	acgttggatgCAACCACAAGCAGCACAAAT	CTAATGTTGGAGGTTATGCT	G	A	104	60.15	CaChr2	3410435	zf-HD
CaTFSNP49	acgttggatgTTTATGATGCGTGCAACCTT	acgttggatgGCTCTGTCCTGCAATCAGGT	AAAGTCATCTTGTATTCAGAACCCTC	A	C	199	59.19	CaChr2	4324332	SAP
CaTFSNP50	acgttggatgGAGGGGAAACAAACATGGAA	acgttggatgCCTTTTCCACTCCCCTTTTC	TGTTATTTCGTCCATTACGGTTG	A	T	199	59.77	CaChr2	5886240	PHD
CaTFSNP51	acgttggatgAGAGGAAAGGGTGGGGTTT	acgttggatgTCTCAAACATGCGTTTCCAG	AACATTACGACGAACTGGCAT	T	C	200	59.78	CaChr2	6181000	SET
CaTFSNP52	acgttggatgGGGTGTCCATTCAGATTACG	acgttggatgGGTTGTGAAACCCACAAACG	TCACCATTTCTTACGGG	C	T	102	46.3	CaChr2	6743024	FAR1
CaTFSNP53	acgttggatgGGCTTCAATTCTTCGAGCAG	acgttggatgTTTTCTTGAGGACCATCACTG	CAAAAACGTTGGTGTC	G	A	116	60.1	CaChr2	9152974	bHLH
CaTFSNP54	acgttggatgAGTAGGCGAAGGAAGAGAAG	acgttggatgGAAGAAATGCATTTGGCGGG	ATTTGGCGGGAACTGT	T	C	98	49.2	CaChr2	9266035	GNAT
CaTFSNP55	acgttggatgAACGCCTTTAACCGAATCCT	acgttggatgCTTCCAACACGGCATCCTAT	GTGAACCATGGCTGGAAAAG	T	C	203	59.97	CaChr2	10330226	bHLH
CaTFSNP56	acgttggatgCCATGGACATGGTCCATGAG	acgttggatgTTTCAATTCCTCTCGCTCCC	CCCCCCTTTACAAATCCACAAAA	G	A	85	46.8	CaChr2	14248647	GNAT
CaTFSNP57	acgttggatgGCAACCTCTCTTTTCTCACC	acgttggatgTGTAAACTCCCTGGAGAGTG	AGCGACTAGAGTATGATTTTTGGAAGT	G	A	107	51	CaChr2	15817338	C2C2-YABBY
CaTFSNP58	acgttggatgTTGGCTCCTCTTCCAGTTTG	acgttggatgTCGAATCTTCAATACAAAACATACC	CCTCACTTTGATTTTCAAGA	G	A	84	60.37	CaChr2	16132823	SBP
CaTFSNP59	acgttggatgCCGGCATTGTTAGACTCGTT	acgttggatgTTGTCATGGCCCCATAATTT	TGAGAAGAAAGTGAACAATGACAAAT	G	C	188	60.13	CaChr2	16353473	GeBP
CaTFSNP60	acgttggatgTCCTCTTCCCAACAAATCCC	acgttggatgCCTTCGTTTCAAGGGATTGG	GCTTGGGAAGGCTGTTGT	T	C	92	53.5	CaChr2	18418063	GNAT
CaTFSNP61	acgttggatgGATGCTGGACGAAATCATCA	acgttggatgTGAATCATCCGTGGTTGAGA	ATCCTAGTGCAGATGAAGGACC	T	C	225	59.61	CaChr2	18878024	bHLH
CaTFSNP62	acgttggatgCGGCGAATTTTGACCTTGAG	acgttggatgGAGATTCCAGAGGCATCAAC	AGGCATCAACTCCGGT	C	G	99	50.9	CaChr2	21714168	zf-HD
CaTFSNP63	acgttggatgTGCATAGCCACCGTCATCTA	acgttggatgAGGGTGAGGGTGGAGGTACT	TGCGAATCAACTTCCATATCAA	T	C	210	60.24	CaChr2	24236619	Alfin-like
CaTFSNP64	acgttggatgATTGGCCTCCTGTCTTCCTT	acgttggatgGGCATGCTGACGATGATAGA	TTCTCTTCTCTCCTATGTTGATCTTCT	G	C	202	60.07	CaChr2	25291561	C3H
CaTFSNP65	acgttggatgGAGGGAAGGGTTTGATGGTT	acgttggatgTCTCCAGGACCACAAAGGTC	GTGGTGGTGGAGTTGGTTC	T	C	207	60.17	CaChr2	26158345	FHA
CaTFSNP66	acgttggatgGCCTCCTGATAAGTTAGCTG	acgttggatgGCAGACTGTCGATTTGCAAG	TCTCATTCTCTTAGCACGTTTGGG	A	G	98	50.7	CaChr2	27361120	bZIP
CaTFSNP67	acgttggatgAACCAACCTTGTTCCTCTTTCA	acgttggatgCCAGCTTCAGCTTCTTGAGC	GCTTCTGTAAAGACATGGTAGAGAGA	G	C	194	60.01	CaChr2	27694185	AP2-EREBP
CaTFSNP68	acgttggatgTTGGAAATCACAGTGCATGA	acgttggatgGATGAAAGTTTCGGGTTTCG	CCTTTATTGAAGTCTCTAGAAGCC	G	A	89	58.62	CaChr2	30377757	MYB
CaTFSNP69	acgttggatgTAGCATCGGAACCTTCTTCC	acgttggatgTTTAGGAGATGCCCAACCAG	TCTTGAGAGTGAAGATGATGATGC	G	A	200	59.27	CaChr2	30698052	HMG
CaTFSNP70	acgttggatgAGTCCGTGTAGTTGAGATAG	acgttggatgGGAGCAAGGAATTCAACAGG	GGGGCTTTGAATCCAACAAACCTTGA	C	T	120	49	CaChr2	30867757	C3H
CaTFSNP71	acgttggatgTCAAATGTGTTTTGGGCAGA	acgttggatgCAGAGCCAACCAAAAGAAGC	TATCCCAGTGAATGGAGCAAA	G	A	203	60.09	CaChr2	32769483	FAR1
CaTFSNP72	acgttggatgAAAGTGTTGCTGCTCCTGGT	acgttggatgGCTGAAGCCTATGCACATCA	CATCAAACCTTAGCTAATATGTACC	G	C	117	59.91	CaChr2	32958106	bZIP
CaTFSNP73	acgttggatgCAAGTACGTGCACCAGCAGT	acgttggatgCCTCCACCTAAATGAAGCTCAG	AAAACATGTCCACCAATCTTCAC	A	T	197	59.97	CaChr2	33604180	SRS
CaTFSNP74	acgttggatgCCAGCTTTAGCGAGGATGAA	acgttggatgGCACAACAAACAGCAAATGG	CGAGGATGAAACTGCCGA	T	C	215	60.48	CaChr2	33661127	HB
CaTFSNP75	acgttggatgATAGTCCTTGCCACTTGACC	acgttggatgTTCAGATAGGATCAGCCAAG	CCGCCAAGATAATTCTGGCAATGTT	T	C	91	52.5	CaChr2	33727257	SNF2
CaTFSNP76	acgttggatgACGACGAAAGGTGGAGAAGA	acgttggatgTCCTGTGGCTTTCCAAAATC	TCCTATATTTTCTTCCTCTTAGGCA	T	C	202	59.84	CaChr2	34132454	NAC
CaTFSNP77	acgttggatgGAGGCTCGGACCATACTGAG	acgttggatgTTCCTCCTCACGATCTGACC	CATCTATGGTTGTGTTTCTCTCTCTACT	G	T	199	59.83	CaChr2	34853120	Bromodomain
CaTFSNP78	acgttggatgGGTCAAGGATTGCACCTCAT	acgttggatgCGAAACACCCTCAGCTTCTC	TGTGAAGTTCATTTACCAAGTACAA	G	A	209	59.93	CaChr2	35412866	mTERF
CaTFSNP79	acgttggatgCTCGTGAACAGGACCGATTT	acgttggatgATCGTCTCCGTTGATCGTCT	AAGATCTCGAAAGACGCGAA	G	A	197	60.11	CaChr2	35556870	CCAAT

SNP IDs*	1st Pre-PCR primer sequence	2nd Pre-PCR primer sequence	Unextended Primer sequence	SNP allele (ICC 4958)	SNP allele (ICC 17160)	Size (bp) of alleles amplified	Actual annealing temperature (0C)	Chromosomes	Physical positions (bp)	Transcription factors (TFs)
CaTFSNP80	acgttggatgTGCAGGCATCAGAACAACAT	acgttggatgTTGTAAACGGCGAAACTTCC	TTTACTGCACGTTGGAATCATC	G	A	214	60.27	CaChr2	36087743	GRAS
CaTFSNP81	acgttggatgACGAGGCACATAGCTCCAAG	acgttggatgCTAAGGGATCCCCAATTGCT	ATCAGAGATTGTTGCATTATTTCTTG	G	A	193	60.42	CaChr2	36337365	GNAT
CaTFSNP82	acgttggatgACCAGGAAGACTCAAACTCG	acgttggatgCACATTTTCCTCAAGTTTCC	GGCCTCAAGTTTCCTAATTTTGAAGC	G	T	111	50.9	CaChr2	36520110	FAR1
CaTFSNP83	acgttggatgGGCTTTCCTTGATGCATACC	acgttggatgTCTCCAATGGACAGAGAAGC	CTCCAATGGACAGAGAAGCAAGAGTC	C	T	116	57.3	CaChr3	2706867	C2C2-CO-like
CaTFSNP84	acgttggatgACCATTAGACCTGCGGTCAC	acgttggatgGGCATCGGCTTTACAAAGAA	TTTTGCCGCGCAGACT	T	C	213	60.00	CaChr3	2709320	C2C2-CO-like
CaTFSNP85	acgttggatgTGTCTTTTTCCAACGGTGTTC	acgttggatgGGCGCATTTTATGGAAGTTG	CACCACCTTAACAACCACCAC	A	C	197	60.00	CaChr3	3617757	zf-HD
CaTFSNP86#	acgttggatgGGCTACAGGTGGCGAAAGTA	acgttggatgGTGTTGTGGCTGCTGTGTCT	AACATGTCGAGAGAGCATCAAC	A	C	200	60.27	CaChr3	4823888	WRKY
CaTFSNP87	acgttggatgCGGAGTGAAGCTTCCTTATC	acgttggatgGATGCCTCCTGAGACTAATG	AAACTTAAGAGAGAAATGAAGATG	C	A	101	47.2	CaChr3	7436178	FHA
CaTFSNP88	acgttggatgAAAGAAGATACTGCGTGCTC	acgttggatgGACCTCGATCGAAAGAGATG	CCACTCTCCTCGGAC	A	G	100	47.4	CaChr3	10493006	PHD
CaTFSNP89	acgttggatgAGGCAATTTAGTCCCTGAGC	acgttggatgGCCGGAATTCTGCAATATGG	CTTTGCATTTGTTCAAGCCAAGATGTT	G	T	105	50.6	CaChr3	14026885	ARF
CaTFSNP90	acgttggatgCTACCGATGGCTCTGCAAAT	acgttggatgTCATGAGCTCCATTGTCTGC	AAGGAAGCTGAACTCTAAAGAAACA	G	A	202	60.24	CaChr3	15739519	FHA
CaTFSNP91	acgttggatgGCTTTGGCTCAGTCACCTGT	acgttggatgATTGCATACTAAACTCAAAGACCTC	ATCTAGATTGAAAGCAAAAAGGATTC	G	A	200	60.45	CaChr3	17174233	LUG
CaTFSNP92	acgttggatgTAGCGTTTAAGGAACCACCC	acgttggatgGCAATGCACAGTTACAGCG	CAGTTACAGCGAAGAGAT	C	T	83	46.5	CaChr3	17181273	LUG
CaTFSNP93	acgttggatgACCCGATAAACGACAGCAAG	acgttggatgCGAAGGTGGCCTAATAGGTG	TCTCTCTTTCACTAGTTTCTCGGC	T	C	200	60.13	CaChr3	17936789	MYB
CaTFSNP94	acgttggatgGGAGGGTTTGATGTCACCAT	acgttggatgCAACCAGGCTGAAAATAGCC	CATGAACAAATTTTCTTGGACT	G	A	98	59.64	CaChr3	20174631	S1Fa-like
CaTFSNP95	acgttggatgACCTAGGGTTTCCAAAACAC	acgttggatgTAAATCCCCCAATGCCATCC	GTAAACCCTTAGAGATCAATCATTCGAT	T	C	101	52.7	CaChr3	20843598	SAP
CaTFSNP96	acgttggatgGCAAAGGTTGGCACCTAAAG	acgttggatgCACCACTCCTTGATGGTCCT	TTGTTGAACAGCAACTATTATCAAG	G	A	227	59.75	CaChr3	21776235	GRAS
CaTFSNP97	acgttggatgCACGGTTGTCACTACGCAAA	acgttggatgTCAAATCCAGTGCCAGACAA	GGTTTGGGCATTGGCG	G	C	211	60.75	CaChr3	22065431	FHA
CaTFSNP98	acgttggatgGCTGGGAGAGGCCTTAATCT	acgttggatgCACAGCCTGCTCCTCATTTT	GCTGACACAGTTGTCATCTA	T	C	87	59.81	CaChr3	23278789	SNF2
CaTFSNP99	acgttggatgAAGGGTCACCATATCCAAGG	acgttggatgGGATCTGATCTGTTTCTTTC	TGATCTGTTTCTTTCAACTTGTTTTCT	C	A	111	52.4	CaChr3	23622995	WRKY
CaTFSNP100	acgttggatgCGGCGTCTGTTAGGTTCTTC	acgttggatgTGATAATGAAAGCCTCCTTGG	TCGGAAGGACTTGTTGAGTC	G	A	102	59.88	CaChr3	23898026	C3H
CaTFSNP101	acgttggatgGATTAATGCGTGTGGGAGGT	acgttggatgAATGCAAATGTCTTGGCACA	TGGGAGGTTGCCCGC	G	A	176	59.82	CaChr3	24542187	mTERF
CaTFSNP102	acgttggatgCTCCTTCACATGGCTTGATG	acgttggatgCCTTATCGGTTCCGAAATCA	AGAATATAATCACCAAGTGTTGAG	G	C	119	59.24	CaChr3	25508396	C2C2-CO-like
CaTFSNP103	acgttggatgTTGCTCCGAAACAACCTCTT	acgttggatgAATCGAAGGATACGCCAATG	CCGGATTTTTCTTTCTCCTCTAC	G	A	209	59.85	CaChr3	25757540	SET
CaTFSNP104	acgttggatgCAGGTCTTAGCAATGACACG	acgttggatgGCCCTATTTCTTCCCATGTC	CCATGTCTTGTTTGGATAC	C	T	81	46.1	CaChr3	26544003	NAC
CaTFSNP105	acgttggatgGAGGAATATGTCTTCTTCCATTCC	acgttggatgTGGAAAGAGTAATTTTCCCCCTA	CTCATGACTGAGTCACTTCCA	G	C	116	59.37	CaChr3	26593875	C2C2-GATA
CaTFSNP106	acgttggatgATGGTTTCCTTCCCCTTGAC	acgttggatgAATGGGAGAGGCTAAGAGTGC	TTAACTCATCTAAACAGTCAGAAATATGAA	G	C	188	60.17	CaChr3	28560960	SAP
CaTFSNP107	acgttggatgGAACTGGGGAGAGTCCAACA	acgttggatgGCACGTATGCCTTTTTCCTC	CAACAGATGATACAGAAGACAAAAATC	G	A	197	60.09	CaChr3	28615172	bZIP
CaTFSNP108	acgttggatgCCACATGAAGCAACGTTCAG	acgttggatgTGCAATGTTTGACGTGGAAT	GGTGCTCCTCTAGCCACCTT	G	C	235	60.3	CaChr3	29843867	bZIP
CaTFSNP109	acgttggatgGAGAAGGGTGATGGAGGTCA	acgttggatgCTCCCCATTCCCTTCTTTTC	GGGATGAGATTGAGGATCTTAGTAG	T	C	194	60.05	CaChr3	30320095	BSD
CaTFSNP110	acgttggatgGGCATGCAAATTTGAGCTTC	acgttggatgGAATTCCGACGTCGCTAAAG	GTAGCGTCTTCCTTAACCACAAT	G	C	197	60.73	CaChr3	30955375	GNAT
CaTFSNP111	acgttggatgCAAATTCACGAGGATGCTGA	acgttggatgGGGTGAACCCAAAGTATGGT	GGTATAGTGTCCCAGGAAGATTTG	G	T	197	59.8	CaChr3	31359638	Jumonji
CaTFSNP112	acgttggatgATGGCTACGCCTCAAATCAT	acgttggatgTTGGAGATGCTCTAACCTTCG	CAGGACAGTATTTTACCAACGCT	A	C	213	59.56	CaChr3	31566643	G2-like
CaTFSNP113	acgttggatgCACAGTTTCTCAGCGTCAGC	acgttggatgACCCTTGTGATCCAGCATTC	GGCCGATGACTTTGAGTT	T	C	99	59.78	CaChr3	32898333	S1Fa-like
CaTFSNP114	acgttggatgGAAATCTCGTTTTGTGTTGTC	acgttggatgACGTGGCTATGCTTTCTGTC	CGTCTGTCCGTAACACACATGA	G	T	93	51.6	CaChr3	32900447	S1Fa-like
CaTFSNP115	acgttggatgTGTGAGGTCAACACGGCTTA	acgttggatgCAAAAGTCGCTTTGTTTCAGG	AAAGAAGGGCTTGAGGGAAA	T	C	202	60.3	CaChr3	33155717	Sigma70-like
CaTFSNP116	acgttggatgTGAAGCATATTTGTCCCTTGG	acgttggatgGGGAAGGGCTACTTCCAGAC	CTGACTGCAGTTCTTTCTGTAGTTG	G	C	209	59.95	CaChr3	34594243	Alfin-like
CaTFSNP117	acgttggatgGGCCTGTCAAAATCCCTTCT	acgttggatgAAATGTCTGCAATCGTGGTG	CGTCCCTCATAGTTACGACTCCT	T	C	201	60.44	CaChr3	34848851	C3H
CaTFSNP118	acgttggatgTTGATGGAACCTGGGTTGAG	acgttggatgAGCATCAAAACCTCTGCACC	CTCTGCACCATCACA	G	A	100	45.2	CaChr3	35935079	Jumonji
CaTFSNP119	acgttggatgTTCCTCATCCTCGATCGTCT	acgttggatgTTCCGCTTGATTTCCTTTTG	ATCTCCTCTTATTATTTCGATTTTATTTG	G	A	202	59.76	CaChr3	36809912	bZIP
CaTFSNP120	acgttggatgCTTCCACCCAACCGTACTTC	acgttggatgTGTTCCCCAAAGATCAGGAA	GTTCCCCAAAGATCAGGAAAG	A	T	204	59.45	CaChr3	37102025	SBP
CaTFSNP121	acgttggatgTCAACAACGGCGATAAACAA	acgttggatgCCTCAACCATCCCAATCCTA	TCAATCAATCCAGATACGTTTTTC	T	C	218	60.11	CaChr3	38614598	GeBP
CaTFSNP122	acgttggatgTGCTCTTCTCAAAGCCAAGC	acgttggatgTTATTTCCACAAGGGCCAGA	CTACAGCGGCGCAAGC	T	C	168	60.8	CaChr3	38760068	bZIP
CaTFSNP123	acgttggatgAACTGGAGCAGCGGTAAAGA	acgttggatgCATTTGCAGCCTCTCCTTTC	TTCTTCCATTTTCAGTGATTTCG	G	T	181	60.02	CaChr3	38914646	mTERF
CaTFSNP124	acgttggatgTAAGGACCCACTTTCCATGC	acgttggatgGGAGAGACTGCATCATCAAGG	GGATATCTGGGTTGACCAATAA	G	C	90	59.93	CaChr3	38969598	MYB
CaTFSNP125	acgttggatgTTCTCCAGCAATGACGACTG	acgttggatgGCCTGCTTCCTTAGTCTGGAT	CAAATGTCACAAGTGCCCC	G	A	198	59.98	CaChr3	39312173	bZIP
CaTFSNP126	acgttggatgGGGAACCGAAGAAGAAATCC	acgttggatgTTTTCGGCCTTTCAGACAAT	GGTGCCGGAGATGGC	G	A	104	59.88	CaChr3	39546588	AP2-EREBP
CaTFSNP127	acgttggatgCCTGTTTTCCTTGGCCTCTA	acgttggatgTGAGGAGGAGGGACTGCTAA	TAAGGTTTAAACCCTGTTCG	G	A	109	59.31	CaChr3	39788772	MYB-related
CaTFSNP128	acgttggatgCCAATACCAATTAGGCATCCA	acgttggatgAGCCGTGGGGGAATAGAATA	CCGGGTAAAATATCTTCCTCTTC	T	C	233	59.67	CaChr3	39816472	ABI3VP1
CaTFSNP129	acgttggatgACATAGATTGGAGGCCAAGG	acgttggatgTAGTTTGGAGGGAGAAGTGG	GGATGATGATAGTGAGAGTG	T	A	99	47.1	CaChr3	39817437	ABI3VP1
CaTFSNP130	acgttggatgGCGGTATGCTTAACTGTTCC	acgttggatgATGATGCAGAGGCTTCATTC	AGTCTTCTTCTTCTTCTTCTTTT	G	T	111	45.1	CaChr4	19353	bZIP
CaTFSNP131	acgttggatgTGAATCGCATGATACCACCA	acgttggatgAGGACAGGTTGTTCCCATGA	TGATACCACCATGCTCTTTGG	A	C	197	60.9	CaChr4	832308	AP2-EREBP
CaTFSNP132	acgttggatgAGCATACAGGTGTGCTGTCG	acgttggatgACATGCTCGGTTTTCTTTGG	GCGTGATTTAATTATGCC	G	T	105	59.93	CaChr4	1047604	SNF2
CaTFSNP133	acgttggatgCAAATCCGGAACTTGCCTTA	acgttggatgCATTTGTTGTTGCTGCTGCT	AATTCCAACGACGAGTTTCC	T	C	188	60.07	CaChr4	1494086	WRKY
CaTFSNP134	acgttggatgTCCAGCAGAAAATGATGGTG	acgttggatgTTGCACACAAGAGTTGAGAACA	GAGAGCACATTACTTCTCACTCC	T	C	204	59.65	CaChr4	1619541	bHLH
CaTFSNP135	acgttggatgTCCAAACACTGCTTCTGGTG	acgttggatgTGGCTTGGTTTGACTGAACA	TGGCCCTGCACAGTACA	T	C	95	59.87	CaChr4	2005078	PHD
CaTFSNP136	acgttggatgGGGTGGTGACCCTTTCTGTA	acgttggatgCTTTATCGGTTGGCCAATGT	GGATTTTAGTAAACAAGAAAATAAAGCAG	G	T	201	59.82	CaChr4	2168233	SAP
CaTFSNP137	acgttggatgTTCACAAAACCCAACCACCC	acgttggatgTGTCAAGAAGAGGTTAGGCG	GAAGAGGTTAGGCGTATGAA	G	A	94	49.5	CaChr4	4276290	Jumonji
CaTFSNP138	acgttggatgTCCATTGTCCAAACATTCCA	acgttggatgCCCTGTAAAGGCGTCTTCTG	GAACTTCCCTTTCTGATTTTGACTA	G	A	198	59.75	CaChr4	4745377	G2-like
CaTFSNP139	acgttggatgCTGGTTGGCTCCACCTTCTA	acgttggatgGGAATTAGTCCCCAGCATCA	CTGGTTGGCTCCACCTTCTA	T	C	199	60.25	CaChr4	5004108	bHLH
CaTFSNP140	acgttggatgTGTTTGGATCGAGGGTCAAT	acgttggatgACGAAATTCCCGACCACTTA	GCTACCTCTCCCCTTTCTTCTTC	G	T	202	60.32	CaChr4	5022053	bZIP
CaTFSNP141	acgttggatgCCAAGGGAAGCTTGTTCTACA	acgttggatgCCTTTGTTTTCATCTGCTGGA	GCTTGTTCTACAATCTGATCAAAA	G	C	108	59.36	CaChr4	5080383	Trihelix
CaTFSNP142	acgttggatgGAGGACAACTGCTGCGAATC	acgttggatgCTTCCTGGTGTGTTGTTGGA	AATTGAACGACTGCCAAATC	T	C	203	60.96	CaChr4	5677430	MYB-related
CaTFSNP143	acgttggatgAATTCCGCCATGAGAGCTAA	acgttggatgCAGGAGAATCGTCACCATCA	GCACGTCATGCTTCCAATC	A	T	208	59.81	CaChr4	5747340	Jumonji
CaTFSNP144	acgttggatgTCTTGGAAAAGAAACCCAACA	acgttggatgAGGTTCCACTTCCATCATGC	AGTTGAATATCAATAAGAAAGAGTTT	G	A	95	59.57	CaChr4	6183406	HB
CaTFSNP145	acgttggatgGGCAAATTTCCCACTTCAGA	acgttggatgTCAAAAGTTTTTCCTGTTTGGTT	TGGTTAAACCAATGACTAGA	T	C	105	60.05	CaChr4	6766479	MYB
CaTFSNP146	acgttggatgTGCCCCAAATTGTCGTATAA	acgttggatgCACTGCAGCTCCTTCTGATG	CCACTTTGAAAATCTCCCAA	T	C	116	58.89	CaChr4	7394399	AP2-EREBP
CaTFSNP147	acgttggatgGCCGTTCGTAGAAGCAGAAC	acgttggatgCCCAGTTGGGTCGTTACTGT	AAGCTCTGAAGATGTTGGAACC	G	C	196	60.02	CaChr4	7974685	C3H
CaTFSNP148	acgttggatgGAGTAGAATGCTCTCTCACG	acgttggatgTTCCGTTAACGATTCAATCC	TCAATCCAAAACTGCTTCT	T	C	107	47.6	CaChr4	8390599	BSD
CaTFSNP149	acgttggatgCCTCAGAATTGAAGTCAACAACA	acgttggatgGAGCCAACACCTGTTAGAGGA	GATAATATATCAGACTCATTTTTCACCAAT	G	C	191	59.27	CaChr4	8471278	AP2-EREBP
CaTFSNP150	acgttggatgTCCCTTTTGCTCCCAAAAAT	acgttggatgTCGCTGAGGAGAGAGAGAATG	AAATCTCAATTCAAAAACCCTAAT	A	T	82	60.77	CaChr4	8769821	NAC
CaTFSNP151	acgttggatgGGGGAGCACAGATTTACGAG	acgttggatgACGATTTCGGATTTGGAATG	GGCGCAGGTAATGATGC	G	A	208	59.69	CaChr4	9471744	AP2-EREBP
CaTFSNP152	acgttggatgTTGCAAGAGGTGAAGTGTGG	acgttggatgGGGGGCAGAAGAGTGATTTT	GAAGTTGAGACACGACTCCATCTA	A	C	177	59.87	CaChr4	10704805	mTERF
CaTFSNP153	acgttggatgCTCGTGCTCACAAGTGCTTC	acgttggatgGTGCCAAGGATGGATCTGTT	AAATGATCATATGGGATTTAGAGCA	G	A	197	59.78	CaChr4	10973664	SAP
CaTFSNP154	acgttggatgGAAAAGTTTGGGCGTTCTCA	acgttggatgATAATCGGAATCGGCGAAC	GGGCGTTCTCATTTGG	G	T	92	60.23	CaChr4	11054722	NAC
CaTFSNP155	acgttggatgTCGACGAGTGAGGATGATGA	acgttggatgCGCGAATCTCTTTCTTCCAC	CCTCGCCTATATAAGAAACCGC	G	A	213	60.36	CaChr4	11600593	Trihelix
CaTFSNP156	acgttggatgCTTGTGGCATTGTGGAATTG	acgttggatgTCATAGGCGCTTCCAAGATT	GCTTCCAAGATTCGACGTAA	G	A	220	59.96	CaChr4	12315577	RWP-RK
CaTFSNP157	acgttggatgCCAGAATGGTCTCCCAATGT	acgttggatgGCCTCAAGAGCTTTGCAGAT	CAAAGCCAAGGTTCCACG	G	T	209	59.78	CaChr4	12318493	RWP-RK
CaTFSNP158	acgttggatgCATCATTAGAGGAACATGG	acgttggatgAGGACATGTGATCCAAAGGC	TTCCAAATCCAAATCAAAATCCATA	G	C	101	50.1	CaChr4	12319718	RWP-RK
CaTFSNP159	acgttggatgTTGGGAGAAGAACTCCATGC	acgttggatgTTTTGCACTTGTTGCTTTCG	TTAGCATGAAAGATTTGCAGA	G	A	101	60.2	CaChr4	12964701	MADS
CaTFSNP160	acgttggatgACAGCCTAGACTGCCTCCAG	acgttggatgCTAGGCCCAATGTTTCTTGC	TTCTTGTCAGTCATACAGCATCG	G	A	190	59.62	CaChr4	13328322	Tify

SNP IDs*	1st Pre-PCR primer sequence	2nd Pre-PCR primer sequence	Unextended Primer sequence	SNP allele (ICC 4958)	SNP allele (ICC 17160)	Size (bp) of alleles amplified	Actual annealing temperature (0C)	Chromosomes	Physical positions (bp)	Transcription factors (TFs)
CaTFSNP161	acgttggatgCTCCTTCAACAAGGGCAGAG	acgttggatgTATGGGAACAATGCATGCTG	AACACAGTAGGGTCAAGATGAAAAG	T	C	196	59.98	CaChr4	13727396	Alfin-like
CaTFSNP162	acgttggatgGGCATATTCGCGTAAACGAT	acgttggatgTGGATCTTGGCCTTTACGTT	TGAGAAGTAGATAGGGCTCAAAGAAA	A	T	221	59.96	CaChr4	14094264	FHA
CaTFSNP163	acgttggatgCCACGAACACGTTTGCTAGT	acgttggatgCCGGAATACCCTCAAGCTAA	TTGGGTGTTCCGTCGAA	G	A	191	58.85	CaChr4	14511914	LIM
CaTFSNP164	acgttggatgACCATCCTGCAGACCTCAGT	acgttggatgAGGAGGGCAGAAAGAGGAAG	TGGTCTCCATAAGGGCATGT	A	T	195	59.71	CaChr4	15539515	C3H
CaTFSNP165	acgttggatgGACTTGTACGACGCCGTTTT	acgttggatgTGATGACAAAGAACGTGGCTA	AAAACCGCTAGAGCTTGTGA	G	A	200	60.18	CaChr4	16279070	C2C2-CO-like
CaTFSNP166	acgttggatgCCAAAAAGGACTTGTTCTGG	acgttggatgAAGTCAAGTCAGATCCACTC	TCCACTCTTAAGAACAAAAA	G	C	119	45.1	CaChr4	16362489	AP2-EREBP
CaTFSNP167	acgttggatgCTTCCACCATTGACATGCAC	acgttggatgAAATGGAGGCGATTCATCTG	TCGTTTCTTTCCCTTAGTAGCG	T	C	206	59.97	CaChr4	17427472	Jumonji
CaTFSNP168	acgttggatgTTTTGGTGAAGACCATGCTG	acgttggatgCCCTCACCAGAGCAACAAAT	TCAGATCATCAACTGTAAGCCG	G	T	203	59.69	CaChr4	17722046	SNF2
CaTFSNP169	acgttggatgGGTGCTCCATTTTGCAGTTT	acgttggatgCGGTTCAGCTCCAGATTCTC	GGTTCCAAAATAGGCGTGCT	G	T	181	60.12	CaChr4	17862210	HB
CaTFSNP170	acgttggatgATGGGACAAATGCAAAGCAG	acgttggatgAATTTTTGCAGTGGCTGAGG	GCGAATTGAGCCCTCAC	A	C	102	61.03	CaChr4	19013085	C3H
CaTFSNP171	acgttggatgTGCAGGAGTTGATGTCAAGAA	acgttggatgTTCCAATGCCTCATTCTTCC	AGAAGAAAAACACACTCCACCAA	A	C	198	59.43	CaChr4	19586476	MYB-related
CaTFSNP172	acgttggatgAAGTCTGCGTATGCATCGAG	acgttggatgAAAGGGTCCGTCAGTTGGAG	CTAAAAGCTCAAAAAGTCTCTTGTC	A	T	105	49	CaChr4	20446814	Sigma70-like
CaTFSNP173	acgttggatgCAAAGACAGTCCCTGAGTAG	acgttggatgGCAGTACAACATGTGGAACG	GAACAATTCAGCGGCGCGTGA	A	G	99	56	CaChr4	23557644	HB
CaTFSNP174	acgttggatgCAAACCCACATCAAATCTTG	acgttggatgCTTCTGAGGTTTTTCCTTGTG	CAAATTACTTGGTAGTGGATCTT	C	T	100	47.6	CaChr4	29778149	HB
CaTFSNP175	acgttggatgGCGTTGAACAAAATCTTGCC	acgttggatgAACTGTTCCTGATGGTACCG	CAACAGAATTTGGTTCGT	G	A	114	45.9	CaChr4	36535887	ABI3VP1
CaTFSNP176	acgttggatgAGAGGTCAGCGAAGGAAACA	acgttggatgGGCCAATTTGTTGCAATTCT	TTGAGTTAAAATATTGAACCAAAGAAAAC	G	A	206	59.99	CaChr4	36974817	SNF2
CaTFSNP177	acgttggatgGAGAATGTTTGTGTGAGGCTCTT	acgttggatgTGAAAAACAAACTGCGGAAA	AAGATACTCAAAACACAAAGGATC	T	C	101	59.81	CaChr4	37061273	SET
CaTFSNP178	acgttggatgTGTCTTGTGGATCACACATATAAAGA	acgttggatgGGCAGGAAGTAGCTGCGATA	CATATAAAGAAGTCCTTCTCACAA	T	C	112	59.83	CaChr4	37096147	ABI3VP1
CaTFSNP179	acgttggatgGGGAGTGGTGATTTCCTTTTC	acgttggatgCATGGAATGTGGACTTGAAGC	CTAGAGAGAAAATAACAACTTGGAATGA	A	T	185	59.8	CaChr4	37097065	ABI3VP1
CaTFSNP180	acgttggatgTTTCACAAATTCAAGCCAACC	acgttggatgTGGAATAAATTCCGTCATCACA	ATAGGGAGTGGTGATTTCCTTTT	A	T	189	59.97	CaChr4	37097068	ABI3VP1
CaTFSNP181	acgttggatgTGTTATCCTTGCTTCGCTGA	acgttggatgCAACCACCATGTTTCCATCA	CTCGCAAAGCATCACTGG	G	A	205	59.57	CaChr4	37514472	Tify
CaTFSNP182	acgttggatgTATCCGGGAGAAACAGATGG	acgttggatgGCTGCATAGGTTGAACACGA	ATTTGAGAATTGAAATCCCTTCC	A	T	203	59.89	CaChr4	38486601	MADS
CaTFSNP183	acgttggatgGCGTTGATGATCCTGAGTCC	acgttggatgGTGATGAAGCTGGAATGCAA	GCTAGCTTTTCAAGTCTTTGTCCTAT	G	C	203	60.63	CaChr4	38659802	GRAS
CaTFSNP184	acgttggatgTGGCGATCAAACTGTTGCTA	acgttggatgTAAAGTCAAGCATGCGTCGT	TAGGGCTGTTATTTCCCTGG	G	A	203	60.4	CaChr4	39804794	Trihelix
CaTFSNP185	acgttggatgCCGAGCGATTTGACAGTACA	acgttggatgTTCATGATCGTCCGGGTATT	AAGTCAGATACAAAAAGCACGAAA	G	A	200	59.86	CaChr4	40230993	Sigma70-like
CaTFSNP186	acgttggatgGGTGGTTTTGGTCAAGGATG	acgttggatgACCAGCAACCAAACCTTCAG	GGTGTTGTTTCTGGAAATAGGGT	A	T	201	60.21	CaChr4	40578365	Jumonji
CaTFSNP187	acgttggatgAACGGTTTCACGTCGTTGTC	acgttggatgGAACTTTTCACGAAGCGTCC	CCCAATTGCCGGAGGA	T	A	100	48.8	CaChr4	40933076	bZIP
CaTFSNP188	acgttggatgAAGTTTCTCTGCAAAAACTCGTG	acgttggatgTGGTGACAGAAACAAAATTGATG	TTCTCTGCAAAAACTCGTGT	G	C	111	59.98	CaChr4	41866592	LUG
CaTFSNP189	acgttggatgTTGCTTAGAGGACGCTGTTG	acgttggatgTGATGATTGAGCCCTTTCGT	GACGCTGTTGGCTTGAC	T	C	91	59.22	CaChr4	42354903	bZIP
CaTFSNP190	acgttggatgTGGTTATGGTAAGCCATT	acgttggatgACTCACCCGATCCTAACT	AGATTCTAATTCTAATT	G	A	91	51.25	CaChr4	43891050	HB
CaTFSNP191	acgttggatgTTCCTTCCTGTTGAATGA	acgttggatgTTCATGGCTTAATGATATTG	ACTGTTTCAATATTATATATAT	G	C	97	38.89	CaChr4	44393302	C2C2-CO-like
CaTFSNP192	acgttggatgGGAATCGCATAGCTTGGTAG	acgttggatgCCCTCCCAATCCAATTCAAC	ACACTCTCAACCTTCAAAG	C	T	118	47.5	CaChr4	44394420	C2C2-CO-like
CaTFSNP193	acgttggatgGGGTCCATTGCAAAGCATA	acgttggatgCGGCTAGGCAAGAGTTTGAA	CAAAGCATATTTAGCTGATTTTCAAT	T	C	203	59.49	CaChr4	45416533	HB
CaTFSNP194	acgttggatgGACAGAGACCATGGGGAAAA	acgttggatgAGTATTCGGCGGAAAAGTGG	GCAGCAGAGATTAGAGATCCACA	T	C	203	59.9	CaChr4	45736499	AP2-EREBP
CaTFSNP195	acgttggatgAGGTGACCATGAGAAAAATGC	acgttggatgTCCACACACTTGCAAACAAA	GCAAGTTTCATATTTGTGAACTACA	T	C	194	59.04	CaChr4	45835578	LIM
CaTFSNP196	acgttggatgTACGCCCTCTTGCTCCTAAA	acgttggatgGGAAGCACTTTCGGGTCTAA	CACTTTCGGGTCTAATGAAA	G	C	107	59.97	CaChr4	46419887	HB
CaTFSNP197	acgttggatgTGTGAAGCGTATAAGCGGTCT	acgttggatgAATCCCCCAATTCACTACCC	CAGGGGAGAACACTTTACGG	T	C	198	59.92	CaChr4	46995447	CCAAT
CaTFSNP198	acgttggatgTTAAGCGGAGAGGAAGACCA	acgttggatgCAAGCAAACATTCGTTGGTG	AAAATGAGATAGAAAATACCCTTCTGTT	A	T	201	59.95	CaChr4	47258388	ABI3VP1
CaTFSNP199	acgttggatgACCGATTCAACCAATTCAGC	acgttggatgTTCCAGACGTGGAAAACTCA	CAATTCAGCCGATTCAACC	G	A	203	59.94	CaChr4	47445593	LIM
CaTFSNP200	acgttggatgATCCTGATCACGGAAACCAG	acgttggatgGGCGTAACATGGATGAAGGT	CAACCCATCTTTGATCTCAAC	G	A	96	59.93	CaChr4	47884183	HMG
CaTFSNP201	acgttggatgGGCTTGGTTCGGATACATTG	acgttggatgGGAGATGTCAAGCTGATTCCA	GTAGGCAAGATTCGCAATTC	G	A	101	60.33	CaChr4	48124866	HB
CaTFSNP202	acgttggatgGATTTGTTGCTTGGTGACGA	acgttggatgGAGCCACAGTTCAACCCACT	GCACCACGGGATTTCCT	G	T	185	59.7	CaChr4	48276063	SWI/SNF-SWI3
CaTFSNP203	acgttggatgGGCTGACCTCGGTATATATG	acgttggatgTCAACCTCCAACTCAAGAAC	TGTTCGAGATTTGCATGATAA	C	T	101	48.3	CaChr4	48384848	ARF
CaTFSNP204	acgttggatgCCTCCTGCAGCAGAATCTTG	acgttggatgTGATCCTAATTACCGTGCAG	TGGCAAGGTTAATGATGATGCAGCAACA	A	G	110	54	CaChr5	166360	NAC
CaTFSNP205	acgttggatgACGACAGTGTTTCGGAATGG	acgttggatgCATGATCAAGCCCTATAGAC	ATTGGTGACATAGGTATCACTT	G	A	98	49.5	CaChr5	7382449	bHLH
CaTFSNP206	acgttggatgTGTTGAAAAATGGGGAGAGG	acgttggatgCCATTTCTTCTCTTGCAGAAAGTT	GAAAAATTGAAATAAAAAGGATTGA	G	A	89	59.9	CaChr5	7681689	MADS
CaTFSNP207	acgttggatgGGAAGTGCCGTTGAATTGAT	acgttggatgTTCATTGGCACAACCATCTC	TTTTCTGGAACATTGACACTTGA	G	A	191	59.94	CaChr5	9958884	WRKY
CaTFSNP208	acgttggatgACTTACTGCCTGAGCGGGTA	acgttggatgATCCTTCAATCGACCACGAG	TGAATTTTCAGATTCTTGCTTGTT	T	C	187	59.9	CaChr5	10989680	FAR1
CaTFSNP209	acgttggatgGGAGGTTAATGAGTTTGCAAC	acgttggatgGAGAAGGAATTGGACTTCCG	GCGTTGGTGTTGCTG	G	A	104	48.7	CaChr5	11384336	C3H
CaTFSNP210	acgttggatgGACGGAATTTATTCCAACCA	acgttggatgTTGACCAAAACAGAACATGGA	TCATTTCTAGTGAGCTTCAAGTC	A	C	87	57.4	CaChr5	14755276	ABI3VP1
CaTFSNP211	acgttggatgTCCAGCTTACTTGAACTTGC	acgttggatgCATACCAGAAGCCAACCTCA	CCCTCATTTATCAGAGAAGCG	G	A	120	48.3	CaChr5	16677409	C3H
CaTFSNP212	acgttggatgTTGGTGGTTGAATAATGTTGTCA	acgttggatgACATGAACCGCAAACAAGTG	GTTGTCACTTGTTGTAGGCTTA	T	C	116	60.14	CaChr5	19048521	GNAT
CaTFSNP213	acgttggatgACCTCATCACGAGGCTTTGA	acgttggatgAGGGATTCGGAGAAGGATGT	CCGCAGCATCACTCCAC	G	T	206	60.8	CaChr5	21801207	FHA
CaTFSNP214	acgttggatgTTCTCGTTCTCCTTCTCCAC	acgttggatgAATCCCTCCCTCTATGGTTC	ACTCTATGGTTCCTCTCGTGCTC	C	G	118	54.5	CaChr5	21801378	FHA
CaTFSNP215	acgttggatgCCCATCAAACAAAGAGAGTTGG	acgttggatgTCAGCTGATGATATGGATGGA	AGAGTTTGTTTGTTTCTCTCCA	A	T	102	60.87	CaChr5	24343946	CCAAT
CaTFSNP216	acgttggatgCAGCCAGCTATGACAGGATTC	acgttggatgTGCATATTTTGACCCTGCATT	TGACCCTGCATTTTAAACTC	T	C	110	59.85	CaChr5	24717637	MYB
CaTFSNP217	acgttggatgTGGATCATTCCCAATGAATC	acgttggatgTTCTGTAGGCTCCTCCTTAG	GAATATTAACACGTTGTTGATTC	A	G	111	45.4	CaChr5	26426790	WRKY
CaTFSNP218	acgttggatgAAATGCTGAGGAAGCGTGAT	acgttggatgGAATCACACCCACCCACTTC	GAGAAAATGTACTATGTTGGTGACG	G	A	202	59.84	CaChr5	27042239	MYB-related
CaTFSNP219	acgttggatgTCGGTTCAAAACCCTAATGC	acgttggatgTCTGTCCAGCACTTGACTCG	GTCAGATACCAACGGAGCATC	G	C	203	59.94	CaChr5	27150196	ABI3VP1
CaTFSNP220	acgttggatgAAGCTGCCAGCCTGATCTAA	acgttggatgTCTTCGTTTTTGCACAATGC	TCACTTGACATCTCTCGGTTCA	T	C	201	60.12	CaChr5	27749046	ARF
CaTFSNP221	acgttggatgGGCATGAAAGGCTATGAACC	acgttggatgTCTTGCACTCGTTTGAATGAA	ATGAAAGGCTATGAACCACG	T	C	118	59.53	CaChr5	28253736	LOB
CaTFSNP222	acgttggatgGCGAGAGGTACACAAAA	acgttggatgAATAACACAATTTGGAAGC	ACAAATCAATTTCTATTT	G	C	73	49.91	CaChr5	29397487	AP2-EREBP
CaTFSNP223	acgttggatgCCCTGCAGGTCCTGAAAGTA	acgttggatgCATGCTGCAATACTGGATGG	AGAGCATGCGAAGTACACCAC	T	C	189	60.25	CaChr5	29790311	FHA
CaTFSNP224	acgttggatgTGTGGCTCCTGATTCATGTC	acgttggatgGATCCATCAGCCACTTGTAG	TAACATGGTACCACCTGATTT	C	G	112	49.7	CaChr5	30439023	MYB-related
CaTFSNP225	acgttggatgGCTAGTGGCTCCAACGGTTA	acgttggatgGTAAGTGTCGCGCCTTGTCT	GATATGGAAAGAAGCAATTCACAGT	A	T	210	60.27	CaChr5	31244017	Bromodomain
CaTFSNP226	acgttggatgAGACGGAAGCATTCATGACC	acgttggatgAAACTGAAACTGGGCTTCCA	ACTCTGTTACCGGATGTGGTG	G	A	192	60.08	CaChr5	33879428	bZIP
CaTFSNP227	acgttggatgGACTGCATAGCATGTAACAC	acgttggatgATGAAGGTCCTCCGAAATGC	GAGAGGTCTAGAAGGCCTAA	G	A	100	50.4	CaChr5	34476592	Tify
CaTFSNP228	acgttggatgAGCAAAAGCCTTTCATGCAG	acgttggatgCAACATCACCCCCAGAGTTC	ACAGAATCTGGCATCACCATC	T	C	204	60.52	CaChr5	34510155	LUG
CaTFSNP229	acgttggatgGCAGATTCACCTCCCATCTC	acgttggatgAAATCTCCGTCTCGCAAATG	GTATCAGAAGTAGCACATGGAATCTC	A	T	201	59.62	CaChr5	34684944	bZIP
CaTFSNP230	acgttggatgTCGGACTTGAAATGGACCTT	acgttggatgGTCTCGGTTGCTGGTTTTGT	TCTCACAGGTTTCAGTAAATAATCACA	G	A	200	59.53	CaChr5	35117329	ARR-B
CaTFSNP231	acgttggatgCCACAGTAGAGCACGGTGAA	acgttggatgGTGCGTTCTCATTCAACTCG	GGTCCAAATCGCTGCCTAC	T	C	192	59.9	CaChr5	35122407	PHD
CaTFSNP232	acgttggatgAATCGGAAGACGTTCGTGAC	acgttggatgCTGCACCACCTGTGATTCC	CTGCGAAGTCTGCGAACA	A	T	199	60.12	CaChr5	35389971	C2C2-CO-like
CaTFSNP233	acgttggatgTCGTTCACCCAATCTTTTGC	acgttggatgCGTGTATGATGTATCATTACCAAAG	TAGCTAGGGCCTAGGGGG	A	T	105	61.01	CaChr5	36046998	HB
CaTFSNP234	acgttggatgCTCCCCCAACAATTTCTCAA	acgttggatgTGGAAATCAAACTCCGGTTC	AACAACAAGAACAAGTGGATGATG	T	C	199	59.9	CaChr5	36211837	BSD
CaTFSNP235	acgttggatgTGCAGCTGACTAAGACATGAAA	acgttggatgTTTGCCACAACAATAAATGACA	AAGCAGTAGAGCTAACATAAAATAT	A	T	120	58.73	CaChr5	36283170	LIM
CaTFSNP236	acgttggatgCAAGGAGATCGACGGTGAAC	acgttggatgCCGATGCAGTAGGTGGAAGT	CTGGATACCTCCACCACCAC	T	C	206	60.66	CaChr5	37147512	zf-HD
CaTFSNP237	acgttggatgGCCTTCCCACACTTTCTCTCT	acgttggatgGGTGAAACCCTAACCCTAACA	CTTCCCACACTTTCTCTCTCTAC	A	C	103	59.87	CaChr5	37485160	C2C2-GATA
CaTFSNP238	acgttggatgTCTCGTTGACCAATGCTGTT	acgttggatgAAGGAAAGAGAAAAGAGCCAAAA	TCTCGTTGACCAATGCTGTT	G	A	201	59.29	CaChr5	37814550	Jumonji
CaTFSNP239	acgttggatgTTTCGAAACATTTCGTGCAA	acgttggatgTCGAAACACGAAGAGTGCTG	CTAATCAAATCACCATAAACATCAGAATA	T	C	197	60.23	CaChr5	37985131	MYB-related
CaTFSNP240	acgttggatgTTTGGTCTGCATTTTCTGTCA	acgttggatgTTCACATGGATTCAGCAACAA	TCTGTCATTTTCGGTTCTTCCT	T	C	205	59.31	CaChr5	38149806	bHLH
CaTFSNP241	acgttggatgTGGGTGGAACTTTCATGTCA	acgttggatgAGAAAGCATTTGTCGCAGGT	ATGTAGAAGAGGCGGTGGC	G	A	203	59.94	CaChr5	38467232	GNAT

SNP IDs*	1st Pre-PCR primer sequence	2nd Pre-PCR primer sequence	Unextended Primer sequence	SNP allele (ICC 4958)	SNP allele (ICC 17160)	Size (bp) of alleles amplified	Actual annealing temperature (0C)	Chromosomes	Physical positions (bp)	Transcription factors (TFs)
CaTFSNP242	acgttggatgGCTTACGTGGATGCAGGTTT	acgttggatgTCTAAAGCTGATGGGCCAAA	CTAAAGGATCAAAATAAATGGGTTGT	T	C	202	60.14	CaChr5	40243059	FAR1
CaTFSNP243	acgttggatgTTTGGCTATGTTCCGTGTGT	acgttggatgTGCTTGTATCAAAGTGGCATT	CAGAAGGTGAATTAGACAATGATGA	T	C	186	59.05	CaChr5	40405468	FHA
CaTFSNP244	acgttggatgAAGAGCTTCATTCTCTTGCTGAA	acgttggatgAAATCGTCGTGCTCGTTTG	AGCTTCATTCTCTTGCTGAAG	A	C	118	59.8	CaChr5	40619677	HB
CaTFSNP245	acgttggatgTGAGCGCGAAATAGTATCCTC	acgttggatgCCCGAAATAGAGGAACAAGC	CTCCGAAACAACTCAACCG	T	C	200	59.47	CaChr5	41313407	SET
CaTFSNP246	acgttggatgAAAGGAGCTGTTCAAGCTCAA	acgttggatgATGCCTATGGGTCCAATTCA	TTTATTCGTCTTCTAATTATTTCTTGTATG	T	C	202	59.25	CaChr5	41478089	SWI/SNF-SWI3
CaTFSNP247	acgttggatgTTCGATTTGCAGAAGTGCTC	acgttggatgTGTTGGAAGGTGACTTCCAG	CCATTCTGTGCGGTTGTCCTTTATTGA	C	T	97	55.6	CaChr5	42527654	CAMTA
CaTFSNP248	acgttggatgTGCTTCACTTCCCCTAGACG	acgttggatgAGGATCTTGAGCCATGTTGG	GCGATTTTCCTAATGGTCTA	A	C	83	60.39	CaChr5	42984383	ARR-B
CaTFSNP249	acgttggatgAGGAAACCTTGCAGCAAATG	acgttggatgTGACCATACTCGTCCCATCA	CGAACCTGCTTAGTCTTGCAT	G	C	201	60.25	CaChr5	43835527	bZIP
CaTFSNP250	acgttggatgTGCATCTTCTTCACCAGAGG	acgttggatgACTTGCAAGGACAGCAGTGA	TTCTTCACCAGAGGCGTC	G	T	117	58.96	CaChr5	44421018	SET
CaTFSNP251	acgttggatgGCTAAACGACTCGCACCAAT	acgttggatgGGGGATCAATTGGTCTTCAG	TGCAGCAGAAAATCCTCCATA	A	T	196	60.28	CaChr5	44560496	HB
CaTFSNP252	acgttggatgGATGGAGTGCCTTCATTGGT	acgttggatgGTGAAGATGCAATTCCCACA	GAAGTTCATCAATCTTCTCAATTTTACTC	G	C	198	59.93	CaChr5	45076994	Sigma70-like
CaTFSNP253	acgttggatgAAGCTGCAGAAAGTCGTGCT	acgttggatgCAGAAGACCACAAGGTGCATT	AGTCTCTTAACCCCAAGATAGAACCT	A	C	210	60.34	CaChr5	46175167	NAC
CaTFSNP254	acgttggatgACACCGGGTTTCTCATGTTC	acgttggatgCGCATCAAAAGGCATTGTGG	GGTTGTTAACAGAAATGGAGGGACC	T	C	97	53.3	CaChr5	46513987	bZIP
CaTFSNP255	acgttggatgTTTGTTTTTGCGGGATGAAT	acgttggatgGCCTCATCAGCTTTTGGTTC	GTTTTGAAAAAGAAAAACCCACATA	G	A	175	60.3	CaChr5	47223799	C3H
CaTFSNP256	acgttggatgGTGCAGCAATTGTACAACCG	acgttggatgGTTGACCATTCACACATCGG	AAATTGGGTGGACAAGAATTTCCTACT	A	G	94	54.7	CaChr5	47734066	NAC
CaTFSNP257	acgttggatgTCACTTCCATGACATGAATC	acgttggatgGTAGGTTTCTTGGTGATCCC	TTTCTTTTAAATTTGGTGATGA	C	G	119	45	CaChr6	789313	GRAS
CaTFSNP258	acgttggatgTTGCTTTCCAAGCCAAGAAT	acgttggatgTGAAGGAGGAGCCATAAGGA	CATAACCACAAAATCTATAAGTTTCCATC	A	C	219	59.82	CaChr6	1609229	LIM
CaTFSNP259	acgttggatgCTTCGGATGCCAATTTGATT	acgttggatgGTCTGCTCCTCGACCAATTC	CATCTTCCCTCAAACTCATCC	G	A	205	59.9	CaChr6	1842423	GeBP
CaTFSNP260	acgttggatgTCAGCAGCAGAGCGTTTATG	acgttggatgAAGGGTTCAGCTGCTCAATG	GCCCTTCTACAACAAAGTCTCTAATC	G	A	188	60.3	CaChr6	2925948	Tify
CaTFSNP261	acgttggatgCAAGTCCAAAGCTGGCAAGT	acgttggatgTTCCCTGATAATGCCAATCA	CAAAGCTGGCAAGTTATAGATGAT	G	A	205	60.43	CaChr6	5506963	bHLH
CaTFSNP262	acgttggatgCCTGACTGGGATATCTTTGC	acgttggatgCTATTCATCGCTGGAAGGAG	CTGAAAGACTCACTCTGAGCA	T	C	116	47.3	CaChr6	5688576	LIM
CaTFSNP263	acgttggatgCCAACTTGGAATGGAGGAGA	acgttggatgAAGCTTCTGGAAGGTGTTGG	CCACAATCACAACCAACACC	G	A	184	60.04	CaChr6	5789222	SBP
CaTFSNP264	acgttggatgGGAGATTGGGTTTTGTGTCG	acgttggatgTGATGATCTGGTGCATTTGAA	GAACTTGAACATGAAGAGGACGTAG	T	C	203	60.35	CaChr6	6015415	NAC
CaTFSNP265	acgttggatgTTTTGGTAGCGTCCAGGAGT	acgttggatgTCCAAGACACGGTCCAAAAT	CAGAATATGGATGGCAGAATGA	G	A	190	59.73	CaChr6	6142063	ARF
CaTFSNP266	acgttggatgGAGCCCAAGATTGGTTGAAG	acgttggatgTAGAAGGGTTGGCAAGCAAT	CCTAAGTTGGATACCATACTCACATC	G	A	209	59.67	CaChr6	6295375	C3H
CaTFSNP267	acgttggatgGCATCTTGCGAGTCTGAGAA	acgttggatgGTTGGAAGGGATGGACTCAA	AACATTAACCTCCTCTGGCTTTAG	T	C	206	59.27	CaChr6	6913579	SNF2
CaTFSNP268	acgttggatgGTCCTTTCGCACCTATTTCG	acgttggatgTTCCATTTCCTCTGCGATTC	TTCCATTTCCTCTGCGATTC	A	T	210	59.71	CaChr6	7275013	MYB
CaTFSNP269	acgttggatgGGCCAGACACCAGAAACAAC	acgttggatgGCAAATCAAATCCAAACAACAA	AGGAAGAACGAGAGAAGAGTTTTTT	A	C	194	60.55	CaChr6	10032665	zf-HD
CaTFSNP270	acgttggatgTCATTTCTGAGTGAACTTGG	acgttggatgGAGCCTGATGGAAAAGTGTC	GTGTCTAGCATTACTGATG	C	T	118	45.2	CaChr6	10748091	mTERF
CaTFSNP271	acgttggatgTGCGATCTCTTCAATTCTGC	acgttggatgAAACAGGGAACCACTTGTCG	CATTAATATCGTATTCGTAACCAGATG	G	A	212	59.1	CaChr6	10864322	CAMTA
CaTFSNP272	acgttggatgGATGAGGGCGAAAATCAAGA	acgttggatgTGCAGAAAAATGCTGCTAAGTC	CCATCAGATCATATAGATAGCAAAAGAA	G	C	186	60.16	CaChr6	13242976	C3H
CaTFSNP273	acgttggatgCCGAGTTGCAAGAGAAAAGAG	acgttggatgCCATGTGATGAGACGTTGCT	CAGAAAATCAGAGTTGAAGAGTT	G	A	101	59.25	CaChr6	13996302	HMG
CaTFSNP274	acgttggatgCTGCAGATTTCTCCACGACA	acgttggatgAATGGTCCACCCATGGATAA	GTTCAGGGGGTTAAATCCAA	G	A	211	59.98	CaChr6	14815110	CAMTA
CaTFSNP275	acgttggatgTAAGCTCATTGAGCTCCGTC	acgttggatgCAACGATGTTGTCGCTAACC	TCACAATACGTGACCTT	C	T	100	45.4	CaChr6	15273685	Bromodomain
CaTFSNP276	acgttggatgGGAATTTGTGTCCCCTGAAA	acgttggatgTGCATGTAAGCTGACGTGTTT	ATCTACTTTTGCTCTATGGCCTTC	T	C	164	59.77	CaChr6	15848050	Jumonji
CaTFSNP277	acgttggatgCGATACAGAACCCACACGAC	acgttggatgGCATCCCAAGTGAAGCTCAT	AAGATCTTACTTCTGCTTTAAGTCTTTG	A	C	203	59.01	CaChr6	18443828	bZIP
CaTFSNP278	acgttggatgGGGAACTTGGATATGGCAGA	acgttggatgTGAGAGGGCTCATCATGACA	GAATTTTGGGGCATTTGGA	A	T	207	59.89	CaChr6	18949559	mTERF
CaTFSNP279	acgttggatgTCCACCGGTTGAGGTTTTAC	acgttggatgCCAATCTTATCTGATGGCTC	CCCCCATCTGATGGCTCAACCTT	C	T	109	48.8	CaChr6	20519235	NAC
CaTFSNP280	acgttggatgCTTCTCCGCCAGTTTCTGAC	acgttggatgAGAGATGGGGCTTGAAGGTC	CATCAGCAGGCTGAACC	A	T	101	59.99	CaChr6	21437289	Bromodomain
CaTFSNP281	acgttggatgACCCCAAAAGGGTCAAAAGA	acgttggatgGCGATTCTTTGCTTGAGAGC	TTGAGCCGATTGTCTATTTGC	G	A	205	60.69	CaChr6	21497337	bZIP
CaTFSNP282	acgttggatgTCGTCAATTGCAGATGTGGT	acgttggatgATATGCCATCATCCCATGCT	TGGAAGGTGTTGAGCAAATAGAA	G	A	211	60.12	CaChr6	21675697	HB
CaTFSNP283	acgttggatgGGGGTCCAATCCACCTTTAC	acgttggatgGGAGACGTGTTGCCAGATTT	CCTTGATGGTTCTTAGATTGATGATT	G	C	200	60.42	CaChr6	21716470	G2-like
CaTFSNP284	acgttggatgTGCATCCGGTTTTCTTTCTC	acgttggatgCCACCAAACCCGATGTTAAA	GAGTTTCTGAATCAGAGATTTTCT	T	C	109	60.19	CaChr6	21777225	AP2-EREBP
CaTFSNP285	acgttggatgACACACAATGTTGCGATTGG	acgttggatgTGGGAGGCCTAACTGAGATG	ATTGGAGGGGAAGGTTCTAG	T	C	100	60.44	CaChr6	22228859	E2F-DP
CaTFSNP286	acgttggatgTGAATGACTGCGCCAAATAG	acgttggatgTGGGTAACTCCTTTGGCAAC	ACTCTGACTCTCTGGCGACAA	G	A	205	59.83	CaChr6	22711105	ARF
CaTFSNP287	acgttggatgTGTCCCTTCCCATGTTGTTT	acgttggatgTGAAGCTCAGGCACATTGTC	TGTTGACACAGACAGAGAAACCAT	T	C	202	60.21	CaChr6	23284891	NAC
CaTFSNP288	acgttggatgTGATCGGGTCTATTGCCTTC	acgttggatgCTCTCAAACTCACAACCGTC	ACCTTCCCCATTAGGCGAC	A	G	116	52.7	CaChr6	24586525	bZIP
CaTFSNP289	acgttggatgTTGCAATTTGAGAGGGGTGT	acgttggatgATTCTTCCTCCCCCTCGAC	CTTCCTCCCCCTCGACTC	A	T	183	60.5	CaChr6	26357644	GRAS
CaTFSNP290	acgttggatgGGTGGCATGTTTCCTGAAGT	acgttggatgTCAGGATTCAACCCTCGAAC	GGAGAGAACGCTTGCCATC	G	T	199	59.97	CaChr6	27861705	C3H
CaTFSNP291	acgttggatgCTCGGCCATACAATTGAACA	acgttggatgATGGAGCATCATCTGGGAAC	TCTTACAGATATGTAGTGGAACTGATGA	G	A	199	59.54	CaChr6	28103045	HB
CaTFSNP292	acgttggatgTTCAGATCATCAAAATCGTGCT	acgttggatgTCTGTTTTGTGCCAAACGTC	ACAGAATCAATCAACAGCTAAATCAT	A	C	190	59.71	CaChr6	30942481	bZIP
CaTFSNP293	acgttggatgATGGCACCACTTTGGAAGTC	acgttggatgCCCATTGTGCTTCTCAGGAC	GGAAGTCCCTGATCCTGA	T	C	88	59.97	CaChr6	31477570	E2F-DP
CaTFSNP294	acgttggatgTAACCGGCGTTCCTCGATTG	acgttggatgGGGCTGGATGTTTCAAAACC	GGTAAAAACCAGGGGAGGC	G	T	113	48.8	CaChr6	31478512	E2F-DP
CaTFSNP295	acgttggatgCATCGGGTTCCTTCTGCTTA	acgttggatgTCCATTAGACTTTTCATGAGTTGC	CATTAGACTTTTCATGAGTTGCTTC	T	C	198	60.21	CaChr6	32082602	C2C2-YABBY
CaTFSNP296	acgttggatgGGCGATCCTAATTGATCACG	acgttggatgGGATCTTGGATTGGCTGCTA	GATGGAGAAGCCGGATC	G	A	84	60.44	CaChr6	32326867	WRKY
CaTFSNP297	acgttggatgTTCACCACCATCAAACAGCC	acgttggatgGGTGGTTTTTTTGGTGAATG	GGTGAATGAAGAGAGAAATTGTTG	G	T	101	50.6	CaChr6	37388155	OFP
CaTFSNP298	acgttggatgCCATTCCAAGGTCAAGGCTA	acgttggatgGCTGGTTGCCCAGTTAGAAA	GTACATCATGATTGTGTTTACCCTC	G	A	205	60.07	CaChr6	45709378	WRKY
CaTFSNP299	acgttggatgGAACCGGTTACTTATCAATGC	acgttggatgAGGTTCGATGTAGAACCTGC	GGTGGAGAACCTGCTAGAACCGTA	T	A	97	50.5	CaChr6	45713690	GRAS
CaTFSNP300	acgttggatgTCTCCAATGGAGGCAACATC	acgttggatgGCTGTTGAGTTGAGAGAAGC	AGAGAAGCTGAACAAGG	T	A	103	46.2	CaChr6	53173286	WRKY
CaTFSNP301	acgttggatgATTGGCAGCCATTGAGAAAT	acgttggatgAATGCTTCTTGGGTGAGTGG	AAAATTCGTGCCAAGCGCGG	T	C	94	59.53	CaChr6	53654154	bHLH
CaTFSNP302	acgttggatgTGGAAAATGGAAAACACCTTG	acgttggatgCCCAACCAACAGTACCAAGA	GCCTCATAATCAATTTAGAGACACTGA	G	A	213	59.82	CaChr6	54359260	GRAS
CaTFSNP303	acgttggatgGTTGGAATGAGCATGGGAAG	acgttggatgCCAATACCAAAACGTTGAGC	GCACTTAATGGAAATGGTTCAG	G	A	182	60.46	CaChr6	55333022	HB
CaTFSNP304	acgttggatgCAACATCTTCTCTTTGAAGC	acgttggatgAACACTCTGTTTTTGAAGGG	GAGGGGTAAAATCGAATAAGAAGGGT	C	A	99	45.9	CaChr6	55637657	ULT
CaTFSNP305	acgttggatgAAGCTTCTTCCAGCCTTTGG	acgttggatgCCATTGACCTGTTGAGCTTG	CTTCTTCCAGCCTTTGGA	T	C	117	60.87	CaChr6	57024461	MADS
CaTFSNP306	acgttggatgCCATTGTGGAGTTTGGGATT	acgttggatgTGGTACTGTGGGATCTGCTG	CAAGACCATGATAACACGACTCA	G	A	200	59.65	CaChr6	57532005	FAR1
CaTFSNP307	acgttggatgGCAAGGAAAAGAACCGTGAC	acgttggatgCTCCCATCCGAGATTTGAAG	CGATTAATGCAGCAAAAGAGTT	T	C	206	59.72	CaChr6	57720386	ABI3VP1
CaTFSNP308	acgttggatgGTCAAAACGATGGCAGGTG	acgttggatgCATGATGAATCCCCAAGGAC	AGACATTAATGGGTTTGGAAGTGT	G	A	212	60.1	CaChr6	58112367	MYB-related
CaTFSNP309	acgttggatgTTTGCCATGGAAGTGATTTTC	acgttggatgGGAAAAACCCAACTCTCTCCA	CACTAAGACTAAGAAGGATAATTTGTGC	A	T	211	59.93	CaChr6	58242882	mTERF
CaTFSNP310	acgttggatgTGTGTGATATTGAGGCATTGG	acgttggatgTTTTTACATGAAGAACTAGGTCCA	TTTTACATGAAGAACTAGGTCCAT	G	T	100	59.41	CaChr6	58555767	bZIP
CaTFSNP311	acgttggatgACAGCCACCGTAAGGTTTTG	acgttggatgCATTTGAACCCGGTTCAGAC	TCAGACCAGACGCAG	C	A	100	47.7	CaChr6	58810441	C2C2-GATA
CaTFSNP312	acgttggatgGCTTCTTCCCATTGCTAACG	acgttggatgTGAACTCTGAAACACACTCTTGC	CTTTAGCTTCCTTGGAAATCTT	T	C	110	59.85	CaChr7	82458	CCAAT
CaTFSNP313	acgttggatgTCTGGAGGACCAACACCAAT	acgttggatgCCGGTTGACTCGCTAGTTATG	TTTTGAGGAATACTTTAACCTGTG	A	C	119	60.36	CaChr7	217223	GRAS
CaTFSNP314	acgttggatgTGAGCTTTCCGTAGGTTGGT	acgttggatgATCAACGGTGGATGATGGAT	CGTTTGCAAGGTTGTGTAGTTAGT	G	A	211	59.73	CaChr7	888424	WRKY
CaTFSNP315	acgttggatgTTTAGAGGCGAGGTTGGTGT	acgttggatgACCACGACACATTCCATCAA	ATTCCGATGAAACCCCTCTT	G	A	209	59.73	CaChr7	1270983	bHLH
CaTFSNP316	acgttggatgAGCGTATTTCGGGGAAAAAG	acgttggatgCCTTTGGCTTCATCCCTACA	ACCCAACTACAATTCGGTCTTC	G	A	207	60.43	CaChr7	1799554	GNAT
CaTFSNP317	acgttggatgACACCTTTTCCGGTGAGTTG	acgttggatgGGTCAAATCCAAATCCCAAA	GGTCAAATCCAAATCCCAAAC	G	T	203	60.01	CaChr7	2712407	WRKY
CaTFSNP318	acgttggatgTAAACCGTTGATGGCGACTT	acgttggatgAGAAACGGCACGGTTTGATA	TTGGTGGTGGGTTTTTATTAG	G	A	109	60.5	CaChr7	3328637	Tify
CaTFSNP319	acgttggatgACCATGGAAACAGTGTAGGC	acgttggatgCGCGTGAGATTAAATTTTTCTTAGAT	CAACTTTAACTATCACAAGCTTTACA	T	C	96	57.51	CaChr7	3353684	ARF
CaTFSNP320	acgttggatgATCTTGGCTTCCGCAGAATC	acgttggatgAGGAGGCTGAATCACTCAAG	AAGTTCACACAAAAGAATCTTAT	C	T	96	47	CaChr7	3794570	SNF2
CaTFSNP321	acgttggatgCTCTCTTTCTCACTCATGCTCTT	acgttggatgTCAGAAATGAAAGATCGAGCA	CAGTCACTTCATTAATCATTCTCTT	T	C	120	57.12	CaChr7	4598134	C2C2-GATA
CaTFSNP322	acgttggatgCAGCATCTCTGCTTTGTCCA	acgttggatgCTGCCGGTTTTCCAAGTTAC	CAGCATGCAAATCCTTTACACA	T	C	201	60.14	CaChr7	4624953	ARR-B

SNP IDs*	1st Pre-PCR primer sequence	2nd Pre-PCR primer sequence	Unextended Primer sequence	SNP allele (ICC 4958)	SNP allele (ICC 17160)	Size (bp) of alleles amplified	Actual annealing temperature (0C)	Chromosomes	Physical positions (bp)	Transcription factors (TFs)
CaTFSNP323	acgttggatgCCACCTTCGATCCACAATCT	acgttggatgGGGTCGCAAGAGAGTATCCA	TCCCAAAAAATTACTCTCCACAA	T	C	211	59.93	CaChr7	5280205	mTERF
CaTFSNP324	acgttggatgAACCAAGTAAAGGTTTGG	acgttggatgAGAGAAGAAGGAGAAGGAT	AAAGAGAGAGAGAATATATAT	T	C	82	50.01	CaChr7	6812937	C2C2-YABBY
CaTFSNP325	acgttggatgTATCGTGGAATTCTGAAGCC	acgttggatgCTATTGAAATCGGATCTGAG	CCTCCATCCGCAAGATACTGATC	T	C	114	46.4	CaChr7	7863835	bHLH
CaTFSNP326	acgttggatgTGGTGGTGAAAACCAACAAC	acgttggatgACAACCACAAGCAGCACAAG	GCTGTTGATGGATGTTTGGA	G	A	191	59.29	CaChr7	8553174	zf-HD
CaTFSNP327	acgttggatgAAAGTTGATTGTGCAGTTTGACA	acgttggatgTCGAAGAGAATTCATGACCAAA	TTGCTAAAATTTTGTCATGTAGTTCAA	G	C	190	59.71	CaChr7	10016133	MYB-related
CaTFSNP328	acgttggatgGTTGCCGTGAAGAGGAAGAG	acgttggatgAAACCCAGTCCAAGTTGACG	TTTGATGTGAACTGTTTTAAAGA	G	T	119	59.99	CaChr7	10114849	AP2-EREBP
CaTFSNP329	acgttggatgTTGAGAAGCTCACAAAAGACTTG	acgttggatgTCGGCGTTTCACTTTCATAA	CAACAACAGTCTTGTTTTTCT	G	A	105	58.82	CaChr7	10251662	C2C2-GATA
CaTFSNP330	acgttggatgCAAGAAGTGATGGTGCAAGG	acgttggatgGCATGACTGCAAATCCTCCT	GACAAAATTAACAAAGCCCGAA	G	A	215	59.29	CaChr7	10485720	SRS
CaTFSNP331	acgttggatgGCCAAGGACTTGCCAACTAT	acgttggatgTGGTTTGCTGAGGAACTCAA	TTGAACACCATGATGAAGTG	T	C	99	59.2	CaChr7	10686471	ARR-B
CaTFSNP332	acgttggatgCTGAAGCAAACAGAGGTGGA	acgttggatgCACCACCACCACTAACACCA	ACCACCACTAACACCACCAAG	G	A	199	59.01	CaChr7	11192772	HB
CaTFSNP333	acgttggatgTGATTCTTTGGCTGAGAACG	acgttggatgCCGGTTGCAATTCATTCTTT	GGTTCGTATGCGGTTCG	A	T	92	59	CaChr7	11324293	bZIP
CaTFSNP334	acgttggatgTGAGAACTTTCCGATCACCG	acgttggatgTCGATGACGTCGGAAGAATC	GCCGACGGTGTGTTTGGAG	A	G	114	50.7	CaChr7	11546194	LOB
CaTFSNP335	acgttggatgGAGGTGGGTTATCCAGATTC	acgttggatgCTTCAAGCTTCCATCAGTTC	GTTCCATTTTTTGTTTGATCACT	A	G	86	48.9	CaChr7	14470793	FHA
CaTFSNP336	acgttggatgAATTCTTCCCAACTTGCCGC	acgttggatgAAACCAACAAGGCAGTGGGC	GGCTGCTTGGACTCG	G	A	102	49.8	CaChr7	17264124	MYB-related
CaTFSNP337	acgttggatgAATTGGTGGGTGGAAACCTT	acgttggatgTGCCCCTCAATTCTCAAAAG	GAATAACCCTTACCTCGATCCC	T	C	211	60.46	CaChr7	17317735	SET
CaTFSNP338	acgttggatgTTTGGCTGTCAAAAGCAAGAT	acgttggatgGCTGTGCAGGGTGTTCATTA	GAATGAGGGATCATATAGCATAAACC	G	A	203	59.87	CaChr7	17780711	bZIP
CaTFSNP339	acgttggatgGGTCAACCAGAAAAGATTCCA	acgttggatgCAGCAGCAAGTGCTCTCATC	CATCAATGATTCAAGAAATGAA	G	A	103	59.02	CaChr7	18389220	Jumonji
CaTFSNP340	acgttggatgCCTCACTTGTACAATCCAAC	acgttggatgGAGGGTGATAGAGGAGAATC	GTGGTGATGATGGTGT	C	T	102	46	CaChr7	20367403	bHLH
CaTFSNP341	acgttggatgCCAACAAAGAAAGCCTGAGAA	acgttggatgCAACAGGACTGCTTCAGTTTG	AAACATGTGGAGAGAGATTAACC	G	A	120	59.48	CaChr7	22826866	bHLH
CaTFSNP342	acgttggatgGGAAAAGAGGACCATAATGC	acgttggatgACAACACACACCACACACAG	AATTAAGCAGTCACGCA	C	A	109	46.9	CaChr7	27161185	Alfin-like
CaTFSNP343	acgttggatgATCCAACAAGTGCACCTATC	acgttggatgTGACTTGGTTTTCTCTAGGG	GGGATTTCTAAGGCAGTCTTTGG	C	T	116	47.6	CaChr7	33299885	Tify
CaTFSNP344	acgttggatgTTCCCAATTTCATTTACATTTCA	acgttggatgCGAACGATGATAGCGAGGAC	ACTAAACGTTACTCTCTTCTTCTTCTTCTT	G	C	99	58.4	CaChr7	39769428	C3H
CaTFSNP345	acgttggatgAGAGAGCACTCATGCCTATC	acgttggatgCCTCTTACTTTCCTGTTGCC	TGGCGGTCAGATCTTTTGA	T	A	104	52.1	CaChr7	42243517	RB
CaTFSNP346	acgttggatgTGTATGCTCGCTTGTTCTGC	acgttggatgACCATGCTCTCCAAAAGTCG	ACGGGAATTACAGCTTTCGAT	T	C	206	60.17	CaChr7	42245921	RB
CaTFSNP347	acgttggatgTGGTAGCGCAAATTCAAAGA	acgttggatgCCCGAGGGGACTACTAGGAA	GCGCAAATTCAAAGATGGG	T	C	211	59.44	CaChr7	42246393	RB
CaTFSNP348	acgttggatgCGGAGTGAGTCGGAATTTTC	acgttggatgTGACTCACTCGGAGTCAAAG	CTGAGGAATTACCAAAGGAGAACATTGT	G	T	96	53.8	CaChr7	43044483	CCAAT
CaTFSNP349	acgttggatgGTTCTTCAATTCAGCATAAG	acgttggatgCTGTGTTGGTGCCTATTTGG	TTGTTATGGAAGCAACTATTTGA	G	A	106	49.1	CaChr7	47237682	HB
CaTFSNP350	acgttggatgGCCTTGGCTTGTGTAATTCC	acgttggatgACCAAGTTGATGGAAAGCTG	GGGAAAGCTGGTACCC	T	C	101	45.8	CaChr8	156866	SNF2
CaTFSNP351	acgttggatgAATTTCCCTCAAGGTGGTGA	acgttggatgTAGTCCGCAAGAAGGACCAC	CAAGGTGGTGATAGTTTGGTTTT	G	A	234	59.38	CaChr8	498219	FHA
CaTFSNP352	acgttggatgCGTGGGATCAATTGAAGAGG	acgttggatgTGCAAGGCGGTTAATTTCTC	AGGACAGGAGAGAAATGTTGGA	G	A	200	60.45	CaChr8	510120	SNF2
CaTFSNP353	acgttggatgGCAAACGTGGCAATACCTCT	acgttggatgAGCAAAGAGGATGGCATCAA	TGGCAATACCTCTACCCCAATA	G	A	200	60.14	CaChr8	666619	GNAT
CaTFSNP354	acgttggatgTCAATGGTAAACCCGAAAGG	acgttggatgGTTCGTCACTTTGGCCATTT	GCAGATTCATACCTGATTGGAAA	G	A	208	59.79	CaChr8	891777	GNAT
CaTFSNP355	acgttggatgGTTGCCCTGACCATGTTCTT	acgttggatgTGGGTTCTTTTGGAAAGAGG	AAGAAACCCTTCTTCACCTTCTTC	T	C	203	59.97	CaChr8	1261904	C3H
CaTFSNP356	acgttggatgGGATAGGGAACAATTCACCG	acgttggatgTTGCCAAAGAACAACCTTCG	CGAACTCTGTTTCCTCTG	C	T	98	47.4	CaChr8	1434072	AP2-EREBP
CaTFSNP357	acgttggatgCCGGAATTGAGGCTACTAAGG	acgttggatgCGGCAATTAGCCTTCTTCAG	CTTGAATTTTCAAAGATAAACCTTCTTC	T	C	209	60.1	CaChr8	1753840	Jumonji
CaTFSNP358	acgttggatgGAATTGAAGATGTAAGGGAGGAAA	acgttggatgCATGTTGTTGCCACCAATTC	AGAAGAGCTGGTAAATTGCC	A	T	99	59.85	CaChr8	2011749	HB
CaTFSNP359	acgttggatgCGCTTTTGATTTTGTCACTCC	acgttggatgTGCGTATTGACGTGGCTTTA	CTTCTATCTCTTTATTTTTTTATTTCTGT	A	C	101	59.74	CaChr8	2580625	AP2-EREBP
CaTFSNP360	acgttggatgAGTGTTCCACCCATTTCGAC	acgttggatgGCAAAACCTGAAGACTGCAA	GATTTGCACTATCTAATCT	G	C	76	59.83	CaChr8	2664948	C2C2-GATA
CaTFSNP361	acgttggatgTGTGCGTGTCTATTTAGCATTG	acgttggatgTTGATGGAGGAATTAGTATGACAGA	TTGTCATAGAAAATGTCCTCAATAT	A	C	85	58.91	CaChr8	2704215	Tify
CaTFSNP362	acgttggatgGGTTTGGCATCCTACACAAG	acgttggatgCCCTTCATTCAGAAGAAGAG	GGTTTGATTTGATGTTGGTAAGGAGTA	C	T	98	51	CaChr8	2974401	ARR-B
CaTFSNP363	acgttggatgTCGTTATGGAGAAATTAAACACATC	acgttggatgTTTGGTTGGCATTGTTGAAA	CGTTATGGAGAAATTAAACACATC	G	T	112	58.56	CaChr8	3154021	bHLH
CaTFSNP364	acgttggatgAAACATCCTGTGCACCCTGT	acgttggatgACTGGGACCAACCATTTCAG	TATGAGGATGATGCCGATGA	T	C	195	60.43	CaChr8	3396641	BSD
CaTFSNP365	acgttggatgGAGAGAAAAGAAGGAATAAACAAATG	acgttggatgGCGGATGTGTTTGGAGAGAC	GAGAAAAGAAGGAATAAACAAATG	A	T	115	58.13	CaChr8	3456812	GNAT
CaTFSNP366	acgttggatgTGAAGCGTTAGCGGTTAATTG	acgttggatgCCGATCAACGACGATATCAA	CGGTTAATTGTGTTAACGCTTT	G	A	96	60.26	CaChr8	3521637	GRAS
CaTFSNP367	acgttggatgCCATTGAAGACAAGGGCGTA	acgttggatgCCTCCTCTTCCCAATTTTCC	AAACTCTAGCTTTAACTCACTTTCCATT	A	T	208	61.02	CaChr8	3682384	LOB
CaTFSNP368	acgttggatgGGAACCAAAAGGCTTCATCA	acgttggatgGGTGGCTCTGGTGATTTTGT	ATCCCACTCACAATCCGAA	G	T	204	60.05	CaChr8	3998501	AP2-EREBP
CaTFSNP369	acgttggatgTATGAGCCAGATAGAGCTCC	acgttggatgCTTCTTTGCACGACCACTTG	CCACTTGAAGGAGCA	C	T	116	45.2	CaChr8	4056195	SET
CaTFSNP370	acgttggatgGCCTCATCACGACTCAAACA	acgttggatgTGATGATGCACTTGAGCGTA	GAAAGCCGGCTTCATCTC	G	C	102	59.84	CaChr8	4176714	FHA
CaTFSNP371	acgttggatgTCATCATCAAATTCGCCTTG	acgttggatgCATATGCCAACGACTTCACG	GCGTCGTCACGCTGAGT	G	A	205	59.62	CaChr8	4421071	LOB
CaTFSNP372	acgttggatgCATTGATCTCTGTGGGGACA	acgttggatgACGACCAAGCAATTCAATCC	GTGGGGACATGTCTGAATC	G	A	101	59.47	CaChr8	4623320	HB
CaTFSNP373	acgttggatgGCATACTCTTCCTCAGACTC	acgttggatgCTATGGTGAGTTCTCAAGCG	CAAGAGGAGTTTAGATGTGTTCGTGT	A	T	93	50.6	CaChr8	5413114	SRS
CaTFSNP374	acgttggatgGCAGACTTGGAATGGCTCTC	acgttggatgGGTCTGAACCGGAGTTTTGA	GAGAATAAGCCCAAAAGCCC	G	T	198	59.96	CaChr8	5508790	C2C2-GATA
CaTFSNP375	acgttggatgCCCAAGTCTCCAAGTGGTGT	acgttggatgCAGTGGAGCCATTACAGCAA	TCATGCCCAATGTTGCTG	T	C	206	60	CaChr8	5564404	C3H
CaTFSNP376	acgttggatgAGTTACTCCTAAGCTTCTCC	acgttggatgGTCTGAAATCAATGATGACG	TGAAGATGATGATGAGTAAAGTTGGC	G	T	109	53.7	CaChr8	6618347	HMG
CaTFSNP377	acgttggatgAAGGAATTTTTCCAGCACCA	acgttggatgTGCCCTACTCTCTTCCCTCA	CTCCCTCTCAAGCTTTTCCAT	G	A	195	59.55	CaChr8	7520805	Trihelix
CaTFSNP378	acgttggatgTTGAGCTGCATGACGACAAT	acgttggatgTGCTAGAACATTCCCCTCCA	CCACAAAGTGATACTGTCACAC	A	T	108	60.42	CaChr8	7772710	E2F-DP
CaTFSNP379	acgttggatgGCCACCATCGTTGGATTTAG	acgttggatgAGGGACAGGAATCATTTGTG	AGGAACAAGGTGGAATTTGA	G	A	111	45.3	CaChr8	8362560	HB
CaTFSNP380	acgttggatgTCCAAGAGTGTTGCATTCAGA	acgttggatgGCTTCGTTGACTCGGTTTTT	TGATTTCTTGCACATATAGTATGG	T	C	99	59.43	CaChr8	11675561	Tify
CaTFSNP381	acgttggatgAAAACCCCTTCATCCGTACC	acgttggatgAACTGATGGAGATGTTTCTG	ATGTTTCTGATTTGTTTGGAA	G	A	108	46.9	CaChr8	12525828	MYB-related
CaTFSNP382	acgttggatgTTGGACCCAAGAAGTTTTGC	acgttggatgCAGTTGCACAAGAAGAGAAGGA	CTTCTTTAGCATCAAAACTTCCAAC	T	C	201	60.09	CaChr8	13906178	C2C2-CO-like
CaTFSNP383	acgttggatgAAAGACCGAACTTTCGCCTC	acgttggatgTGGTCGAATCGGCTTATAGG	GGGTTATATTAATTTCGGTGTTTC	A	G	111	46.1	CaChr8	14719923	SWI/SNF-SWI3
CaTFSNP384	acgttggatgATCTCAGCCATTGGATCTTC	acgttggatgGATACGAGCTAATCCAACGG	CCCAATCCAACGGTCCACG	T	A	86	49.5	CaChr8	16090020	C3H

*CaTFSNP *Cicer arietinum* Transcription Factor gene-derived SNP

Supplementary Table S5: Detailed characteristics of an inter-specific genetic linkage map of chickpea

**Linkage groups (LGs)**	**Genomic and genic SSR + SNP markers mapped**	**Map length covered (cM)**	**Average inter-marker distance (cM)**
LG1	41 + 42 = 83	136.70	1.647
LG2	47 + 36 = 83	88.66	1.068
LG3	58 + 45 = 103	114.78	1.114
LG4	90 + 72 = 162	157.80	0.974
LG5	62 + 50 = 112	117.28	1.047
LG6	80 + 53= 133	152.58	1.147
LG7	60 + 36 = 96	110.05	1.146
LG8	30 + 32 = 62	71.57	1.154
**Total**	**468 + 366 = 834**	**949.42**	**1.138**

Supplementary Table S6: Statistical measures estimated for three yield-contributing quantitative traits in parental accessions and RIL mapping individuals

**Traits**	**Years***	**Parental genotypes**		**RIL mapping population**			**Heritability (%)**
		**ICC 4958**	**ICC 17160**	**Mean ± S.D.**	**Range**	**Coefficient of variation (CV)**	
Number of branches per plant (NB)	2011-12	22.5 ± 0.62	19.0 ± 0.46	20.3 ± 2.8	14.7-31.0	0.136	72
	2012-13	20.7 ± 1.12	17.8 ± 0.89	19.6 ± 2.3	13.8-30.4	0.129	71
Number of pods per plant (NP)	2011-12	102.0 ± 0.57	61.0 ± 0.76	76.5 ± 15.6	48.5-142.2	0.204	76
	2012-13	101.6 ± 0.88	59.7 ± 1.02	73.7 ± 13.9	45.7-141.8	0.198	79
100-seed weight (g) (SW)	2011-12	31.5 ± 0.27	15.7 ± 0.52	19.3 ± 2.9	14.0-32.0	0.151	86
	2012-13	33.8 ± 0.38	16.9 ± 0.66	21.4 ± 3.4	15.2-33.5	0.163	88

*One-way ANOVA probabilities (F-Prob) at $p \leq 0.001$ showing significant trait variation in RILs across two years

Supplementary Table S7: Pearson's correlation coefficients measured among three quantitative agronomic traits in RILs

**Traits**	**Number of branches per plant (NB)**	**Number of pods per plant (NP)**	**100-seed weight (SW)**
**Number of branches per plant (NB)**			
**Number of pods per plant (NP)**	0.86**		
**100-seed weight (SW)**	0.32**	-0.27**	

**Significance at $p < 0.001$

*Significance at $p < 0.05$

Supplementary Table S8: Structural and functional annotation of genomic region (239.1 kb) harbouring one identified strong SW trait-associated robust QTL (*qSW4.2*) resequenced through Illumina MiSeq NGS platform

Marker IDs	Chromosomes	Physical position (bp)	SNPs/SSRs	Genes/intergenic	Putative function
CaSSR248	Ca-Chr4	48145725	(ATA)6		Thioredoxin
CaSNP1	Ca-Chr4		A/G	Intergenic	
CaSNP2	Ca-Chr4		A/G	Intergenic	
CaSNP3	Ca-Chr4		A/C	Intergenic	
CaSNP4	Ca-Chr4		A/G	Intergenic	
CaSNP5	Ca-Chr4		T/C	Intergenic	
CaSNP6	Ca-Chr4		A/G	Intergenic	
CaSNP7	Ca-Chr4		A/G	Intergenic	
CaSNP8	Ca-Chr4		A/C	Intergenic	
CaSNP9	Ca-Chr4		T/C	Intergenic	
CaSNP10	Ca-Chr4		T/C	Intergenic	
CaSNP11	Ca-Chr4		A/G	Intergenic	
CaSNP12	Ca-Chr4		G/T	Intergenic	
CaSNP13	Ca-Chr4		G/A	Ca_10771	GTP-binding protein
CaSNP14	Ca-Chr4		A/G	Ca_10771	GTP-binding protein
CaSNP15	Ca-Chr4		T/C	Ca_10771	GTP-binding protein
CaSNP16	Ca-Chr4		T/C	Ca_10771	GTP-binding protein
CaSNP17	Ca-Chr4		T/C	Ca_10771	GTP-binding protein
CaSNP18	Ca-Chr4		A/G	Ca_10771	GTP-binding protein
CaSNP19	Ca-Chr4		A/C	Ca_10771	GTP-binding protein
CaSNP20	Ca-Chr4		A/T	Ca_10771	GTP-binding protein
CaSNP21	Ca-Chr4		A/G	Ca_10771	GTP-binding protein
CaSNP22	Ca-Chr4		T/G	Ca_10771	GTP-binding protein
CaSNP23	Ca-Chr4		T/G	Ca_10771	GTP-binding protein
CaSNP24	Ca-Chr4		A/G	Ca_10771	GTP-binding protein
CaSNP25	Ca-Chr4		A/G	Ca_10771	GTP-binding protein
CaSNP26	Ca-Chr4		T/C	Ca_10771	GTP-binding protein
CaSNP27	Ca-Chr4		T/G	Ca_10771	GTP-binding protein
CaSNP28	Ca-Chr4		T/C	Ca_10770	ERF
CaSNP29	Ca-Chr4		T/C	Ca_10770	ERF
CaSNP30	Ca-Chr4		T/G	Ca_10770	ERF
CaSNP31	Ca-Chr4		C/G	Ca_10770	ERF
CaSNP32	Ca-Chr4		A/G	Ca_10770	ERF
CaSNP33	Ca-Chr4		T/G	Ca_10770	ERF
CaSNP34	Ca-Chr4		T/C	Ca_10770	ERF
CaSNP35	Ca-Chr4		T/C	Ca_10770	ERF
CaSNP36	Ca-Chr4		T/C	Ca_10770	ERF
CaSNP37	Ca-Chr4		T/G	Ca_10770	ERF
CaSNP38	Ca-Chr4		C/A	Ca_10770	ERF
CaSSR1	Ca-Chr4		(TTA)6	Intergenic	
CaSSR2	Ca-Chr4		(TTA)5	Intergenic	
CaSSR3	Ca-Chr4		(AT)6	Intergenic	
CaSSR4	Ca-Chr4		(ATT)23	Intergenic	
CaSNP39	Ca-Chr4		A/C	Intergenic	
CaSNP40	Ca-Chr4		T/C	Intergenic	
CaSNP41	Ca-Chr4		T/G	Intergenic	
CaSNP42	Ca-Chr4		T/G	Intergenic	
CaSNP43	Ca-Chr4		G/C	Intergenic	
CaSNP44	Ca-Chr4		T/C	Intergenic	
CaSNP45	Ca-Chr4		T/C	Intergenic	
CaSNP46	Ca-Chr4		T/C	Intergenic	
CaSNP47	Ca-Chr4		T/C	Intergenic	
CaSNP48	Ca-Chr4		G/A	Intergenic	
CaSSR5	Ca-Chr4		(TA)7	Intergenic	
CaSSR6	Ca-Chr4		(TAT)5	Intergenic	
CaSSR7	Ca-Chr4		(AT)8	Intergenic	
CaSSR8	Ca-Chr4		(ATT)17	Intergenic	
CaSSR9	Ca-Chr4		(TA)6	Intergenic	
CaSSR10	Ca-Chr4		(AG)7	Ca_10767	DUF702
CaSSR11	Ca-Chr4		(TC)10	Intergenic	
CaSSR12	Ca-Chr4		(AT)12	Intergenic	
CaSSR13	Ca-Chr4		(AT)13	Intergenic	
CaSSR14	Ca-Chr4		(ATT)6	Intergenic	
CaSNP49	Ca-Chr4		A/G	Ca_10763	Med11
CaSNP50	Ca-Chr4		A/T	Ca_10763	Med11
CaSNP51	Ca-Chr4		A/G	Ca_10763	Med11
CaSNP52	Ca-Chr4		A/G	Ca_10763	Med11
CaSSR15	Ca-Chr4		(TTA)7	Intergenic	
CaSSR16	Ca-Chr4		(TAT)17	Intergenic	
CaSNP53	Ca-Chr4		A/G	Intergenic	

Marker IDs	Chromosomes	Physical position (bp)	SNPs/SSRs	Genes/intergenic	Putative function
CaSNP54	Ca-Chr4		A/G	Intergenic	
CaSNP55	Ca-Chr4		A/G	Intergenic	
CaSNP56	Ca-Chr4		A/G	Intergenic	
CaSNP57	Ca-Chr4		T/A	Intergenic	
CaSNP58	Ca-Chr4		A/G		
CaSNP59	Ca-Chr4		T/G		
CaSNP60	Ca-Chr4		T/C		
CaSNP61	Ca-Chr4		A/G		
CaSNP62	Ca-Chr4		A/G		
CaSNP63	Ca-Chr4		T/G		
CaSNP64	Ca-Chr4		A/T		
CaSNP65	Ca-Chr4		T/C		
CaSNP66	Ca-Chr4		T/C		
CaSNP67	Ca-Chr4		T/C		
CaSNP68	Ca-Chr4		A/G		
CaSNP69	Ca-Chr4		A/C		
CaSNP70	Ca-Chr4		A/G		
CaSNP71	Ca-Chr4		A/G		
CaSNP72	Ca-Chr4		A/G		
CaSNP73	Ca-Chr4		A/G		
CaSNP74	Ca-Chr4		A/C		
CaSNP75	Ca-Chr4		A/G		
CaSNP76	Ca-Chr4		A/G		
CaSNP77	Ca-Chr4		A/G		
CaSNP78	Ca-Chr4		C/G		
CaSNP79	Ca-Chr4		A/C		
CaSNP80	Ca-Chr4		T/C		
CaSNP81	Ca-Chr4		T/C		
CaSNP82	Ca-Chr4		A/G		
CaSNP83	Ca-Chr4		A/G		
CaSNP84	Ca-Chr4		A/G		
CaSNP85	Ca-Chr4		T/G		
CaSNP86	Ca-Chr4		A/G		
CaSNP87	Ca-Chr4		T/C		
CaSNP88	Ca-Chr4		A/G	Ca_10760	Amine oxidase
CaSNP89	Ca-Chr4		T/C		
CaSNP90	Ca-Chr4		T/C		
CaSNP91	Ca-Chr4		T/C		
CaSNP92	Ca-Chr4		T/C		
CaSNP93	Ca-Chr4		A/G		
CaSNP94	Ca-Chr4		A/G		
CaSNP95	Ca-Chr4		A/G		
CaSNP96	Ca-Chr4		T/G		
CaSNP97	Ca-Chr4		T/C		
CaSNP98	Ca-Chr4		A/G		
CaSNP99	Ca-Chr4		A/C		
CaSNP100	Ca-Chr4		A/G		
CaSNP101	Ca-Chr4		A/G		
CaSNP102	Ca-Chr4		T/C		
CaSNP103	Ca-Chr4		T/C		
CaSNP104	Ca-Chr4		A/G		
CaSNP105	Ca-Chr4		A/G		
CaSNP106	Ca-Chr4		A/G		
CaSNP107	Ca-Chr4		A/G		
CaSNP108	Ca-Chr4		A/G		
CaSNP109	Ca-Chr4		T/C		
CaSNP110	Ca-Chr4		A/G		
CaSNP111	Ca-Chr4		A/G		
CaSNP112	Ca-Chr4		A/G		
CaSNP113	Ca-Chr4		A/C		
CaSNP114	Ca-Chr4		T/C		
CaSNP115	Ca-Chr4		T/G		
CaSNP116	Ca-Chr4		C/A		
CaSNP117	Ca-Chr4		T/C		
CaSNP118	Ca-Chr4		A/G		
CaSSR17	Ca-Chr4		(CA)6	Intergenic	
CaSSR18	Ca-Chr4		(AT)23	Intergenic	
CaSSR19	Ca-Chr4		(AT)14	Intergenic	
CaSSR20	Ca-Chr4		(AT)14	Intergenic	
CaSSR21	Ca-Chr4		(AT)8	Ca_10759	Glyoxal oxidase
CaSSR22	Ca-Chr4		(AT)12		
CaTFSNP202	Ca-Chr4		G/T	Gene	SNF-SWI3

Marker IDs	Chromosomes	Physical position (bp)	SNPs/SSRs	Genes/intergenic	Putative function
CaSNP119	Ca-Chr4		A/G	Ca_10757	Histone H3
CaSSR23	Ca-Chr4		(GA)9	Intergenic	
CaSNP120	Ca-Chr4		T/C	Intergenic	
CaSSR24	Ca-Chr4		(AT)12	Intergenic	
CaSSR25	Ca-Chr4		(TA)13	Intergenic	
CaSSR26	Ca-Chr4		(TTTA)5	Intergenic	
CaSNP121	Ca-Chr4	48268357	T/C	Ca_10755	bZIP
CaSNP122	Ca-Chr4		T/C		
CaSNP123	Ca-Chr4		T/C		
CaSNP124	Ca-Chr4		A/G		
CaSNP125	Ca-Chr4		A/G		
CaSNP126	Ca-Chr4		T/C		
CaSNP127	Ca-Chr4		A/G		
CaSNP128	Ca-Chr4		A/G		
CaSNP129	Ca-Chr4		A/G		
CaSNP130	Ca-Chr4		T/C		
CaSNP131	Ca-Chr4		T/C		
CaSNP132	Ca-Chr4		A/G		
CaSNP133	Ca-Chr4		A/G	Intergenic	
CaSNP134	Ca-Chr4			Ca_10766	Zinc finger (B-box)
CaSSR27	Ca-Chr4		(TA)6	Intergenic	
CaSNP135	Ca-Chr4		T/G	Intergenic	
CaSNP136	Ca-Chr4		(AT)13	Intergenic	
CaSSR28	Ca-Chr4		(AT)8	Intergenic	
CaTFSNP175	Ca-Chr4		G/A	Gene	ABI3VP1
CaSNP137	Ca-Chr4		T/C		
CaSNP138	Ca-Chr4		A/G		
CaSNP139	Ca-Chr4		G/A		
CaSNP140	Ca-Chr4		C/G		
CaSNP141	Ca-Chr4		T/C		
CaSNP142	Ca-Chr4		T/C		
CaSNP143	Ca-Chr4		T/C		
CaSNP144	Ca-Chr4		T/C		
CaSNP145	Ca-Chr4		C/G		
CaSNP146	Ca-Chr4		T/C		
CaSNP147	Ca-Chr4		T/C		
CaSNP148	Ca-Chr4		T/G		
CaSNP149	Ca-Chr4		T/C		
CaSNP150	Ca-Chr4		A/G		
CaSNP151	Ca-Chr4		T/C		
CaSNP152	Ca-Chr4		T/C	Intergenic	
CaSNP153	Ca-Chr4		T/C	Intergenic	
CaSNP154	Ca-Chr4		G/A	Intergenic	
CaSNP155	Ca-Chr4		C/G	Intergenic	
CaSNP156	Ca-Chr4		G/A	Intergenic	
CaSNP157	Ca-Chr4		A/G	Intergenic	
CaSNP158	Ca-Chr4		A/T	Intergenic	
CaSNP159	Ca-Chr4		T/C	Intergenic	
CaSNP160	Ca-Chr4		T/C	Intergenic	
CaSNP161	Ca-Chr4		T/C	Intergenic	
CaSNP162	Ca-Chr4		T/C	Intergenic	
CaSNP163	Ca-Chr4		A/G	Intergenic	
CaSNP164	Ca-Chr4		T/C	Intergenic	
CaSNP165	Ca-Chr4		T/C	Intergenic	
CaSNP166	Ca-Chr4		C/T	Intergenic	
CaSNP167	Ca-Chr4		A/G	Intergenic	
CaSNP168	Ca-Chr4		T/G	Intergenic	
CaSNP169	Ca-Chr4		G/T	Intergenic	
CaSNP170	Ca-Chr4		C/T	Intergenic	
CaSNP171	Ca-Chr4		A/G	Intergenic	
CaSNP172	Ca-Chr4		T/C	Intergenic	
CaSNP173	Ca-Chr4		T/C	Intergenic	
CaSNP174	Ca-Chr4		A/G	Intergenic	
CaSNP175	Ca-Chr4		T/C	Intergenic	
CaSNP176	Ca-Chr4		G/A	Intergenic	
CaSNP177	Ca-Chr4		C/T	Intergenic	
CaSNP178	Ca-Chr4		C/T	Intergenic	
CaSNP179	Ca-Chr4		C/A	Intergenic	
CaSNP180	Ca-Chr4		C/G	Intergenic	
CaSNP181	Ca-Chr4		T/C	Intergenic	
CaSNP182	Ca-Chr4		C/G	Intergenic	
CaSNP183	Ca-Chr4		T/C		

Marker IDs	Chromosomes	Physical position (bp)	SNPs/SSRs	Genes/intergenic	Putative function
CaSNP184	Ca-Chr4		T/C	Ca_10754	Cytochrome P450
CaSNP185	Ca-Chr4		A/G		
CaSNP186	Ca-Chr4		T/G		
CaSNP187	Ca-Chr4		C/T		
CaSNP188	Ca-Chr4		T/G		
CaSNP189	Ca-Chr4		T/A		
CaSNP190	Ca-Chr4		A/G		
CaSNP191	Ca-Chr4		A/G		
CaSNP192	Ca-Chr4		A/G		
CaSNP193	Ca-Chr4		A/G	Ca_10753	Ribosomal protein L13
CaSNP194	Ca-Chr4	48331023	A/G		
CaSNP195	Ca-Chr4		T/C	Intergenic	
CaSSR29	Ca-Chr4		(AAT)5	Intergenic	
CaSSR30	Ca-Chr4		(TTG)5	Intergenic	
CaSSR31	Ca-Chr4		(AT)8	Intergenic	
CaSNP196	Ca-Chr4		T/G	Intergenic	
CaSNP197	Ca-Chr4		A/G	Intergenic	
CaSNP198	Ca-Chr4		T/C	Intergenic	
CaSNP199	Ca-Chr4		A/G	Intergenic	
CaSNP200	Ca-Chr4		A/G	Intergenic	
CaSNP201	Ca-Chr4		A/G	Intergenic	
CaSNP202	Ca-Chr4		A/G	Intergenic	
CaSSR32	Ca-Chr4		(AT)8	Intergenic	
CaSNP203	Ca-Chr4		T/G	Intergenic	
CaSNP204	Ca-Chr4		T/C	Intergenic	
CaSNP205	Ca-Chr4		T/C	Intergenic	
CaSNP206	Ca-Chr4		T/C	Intergenic	
CaSNP207	Ca-Chr4		A/G	Intergenic	
CaSNP208	Ca-Chr4		A/G	Intergenic	
CaSNP209	Ca-Chr4		T/G	Intergenic	
CaSNP210	Ca-Chr4		A/G	Intergenic	
CaSNP211	Ca-Chr4		A/G	Intergenic	
CaSNP212	Ca-Chr4		A/G	Intergenic	
CaSNP213	Ca-Chr4		A/G	Intergenic	
CaSNP214	Ca-Chr4		A/G	Intergenic	
CaSNP215	Ca-Chr4		T/G	Intergenic	
CaSNP216	Ca-Chr4		T/C	Intergenic	
CaSNP217	Ca-Chr4		A/G	Intergenic	
CaSNP218	Ca-Chr4		A/C	Intergenic	
CaSNP219	Ca-Chr4		T/C	Intergenic	
CaSNP220	Ca-Chr4		T/G	Intergenic	
CaSNP221	Ca-Chr4		T/C	Intergenic	
CaSNP222	Ca-Chr4		T/C	Intergenic	
CaSNP223	Ca-Chr4		A/C	Intergenic	
CaSSR33	Ca-Chr4		(GATAGA)5	Ca_10750	Unknown expressed protein
CaSNP224	Ca-Chr4		A/G		
CaSNP225	Ca-Chr4		T/C		
CaSNP226	Ca-Chr4		A/G		
CaSNP227	Ca-Chr4		T/C		
CaSNP228	Ca-Chr4		T/C		
CaSNP229	Ca-Chr4		T/C		
CaSNP230	Ca-Chr4		T/C		
CaSNP231	Ca-Chr4		A/G		
CaSNP232	Ca-Chr4		A/G		
CaSNP233	Ca-Chr4		C/T		
CaSNP234	Ca-Chr4		T/G		
CaSNP235	Ca-Chr4		T/C		
CaSNP236	Ca-Chr4		A/G		
CaSNP237	Ca-Chr4		A/G		
CaSNP238	Ca-Chr4		T/C		
CaSNP239	Ca-Chr4		A/G		
CaSNP240	Ca-Chr4		A/G		
CaSNP241	Ca-Chr4		A/G		
CaSNP242	Ca-Chr4		A/C		
CaSNP243	Ca-Chr4		T/C		
CaSNP244	Ca-Chr4		T/C		
CaSNP245	Ca-Chr4		T/C		
CaSNP246	Ca-Chr4		A/T		
CaSNP247	Ca-Chr4		T/C		
CaSNP248	Ca-Chr4		T/C		
CaSNP249	Ca-Chr4		A/G		
CaSNP250	Ca-Chr4		T/C		

Marker IDs	Chromosomes	Physical position (bp)	SNPs/SSRs	Genes/intergenic	Putative function
CaSNP251	Ca-Chr4		A/G		
CaSNP252	Ca-Chr4		A/C		
CaSNP253	Ca-Chr4		T/C		
CaSNP254	Ca-Chr4		A/G		
CaSNP255	Ca-Chr4		A/G		
CaSNP256	Ca-Chr4		A/G		
CaSNP257	Ca-Chr4		A/C		
CaSNP258	Ca-Chr4		A/G		
CaSNP259	Ca-Chr4		T/C		
CaSNP260	Ca-Chr4		T/G		
CaSNP261	Ca-Chr4		T/C		
CaSNP262	Ca-Chr4		C/A		
CaSNP263	Ca-Chr4		A/G		
CaSNP264	Ca-Chr4		A/C		
CaSNP265	Ca-Chr4		T/C		
CaSNP266	Ca-Chr4		A/C		
CaSNP267	Ca-Chr4		T/C		
CaSNP268	Ca-Chr4		T/C		
CaSNP269	Ca-Chr4		G/A		
CaSNP270	Ca-Chr4		A/G		
CaSNP271	Ca-Chr4		A/G		
CaSNP272	Ca-Chr4		A/G		
CaSNP273	Ca-Chr4		A/G		
CaSNP274	Ca-Chr4		T/G		
CaSNP275	Ca-Chr4		T/C		
CaSNP276	Ca-Chr4		T/C	Intergenic	
CaSNP277	Ca-Chr4		T/C	Intergenic	
CaSNP278	Ca-Chr4		T/C	Intergenic	
CaSNP279	Ca-Chr4		A/G	Intergenic	
CaSSR34	Ca-Chr4		(TAT)6	Intergenic	
CaSNP280	Ca-Chr4		T/A		
CaSNP281	Ca-Chr4		A/G		
CaSNP282	Ca-Chr4		T/A		
CaSNP283	Ca-Chr4		A/G		
CaSNP284	Ca-Chr4		T/C		
CaSNP285	Ca-Chr4		T/C		
CaSNP286	Ca-Chr4		T/C		
CaSNP287	Ca-Chr4		A/C		
CaSNP288	Ca-Chr4		T/C	Ca_10749	DUF248
CaSNP289	Ca-Chr4		A/G		
CaSNP290	Ca-Chr4		A/G		
CaSNP291	Ca-Chr4		T/G		
CaSNP292	Ca-Chr4		T/A		
CaSNP293	Ca-Chr4		T/C		
CaSNP294	Ca-Chr4		T/C		
CaSNP295	Ca-Chr4		T/A		
CaSNP296	Ca-Chr4		G/C		
CaSNP297	Ca-Chr4	48381171		Ca_10748	AUX/IAA protein
CaTFSNP203	Ca-Chr4	48384848	C/T		ARF

Structurally and functionally annotated 62.7 kb sequenced QTL interval (obtained by QTL region-specific association analysis) are marked with red coloured text
